# Trends in mortality and disability from ischaemic stroke in Europe, 1990-2023

**DOI:** 10.1093/esj/aakag082

**Published:** 2026-07-21

**Authors:** Francesco Brigo, Andrea Zini, Sergiu Groppa, Yaroslav Winter, Piergiorgio Lochner

**Affiliations:** Innovation, Research and Teaching Service, Teaching Hospital of the Paracelsus Medical Private University (PMU), Azienda Sanitaria dell'Alto Adige, Bolzano-Bozen, Italy; Department of Neurology and Stroke Center, Maggiore Hospital, IRCCS Istituto delle Scienze Neurologiche di Bologna, Bologna, Italy; Department of Neurology, Saarland University Medical Center, Homburg, Germany; Department of Neurology, Saarland University, Campus Homburg, Building 90, Kirrberger Straße, Homburg 66421, Germany; Department of Neurology, Saarland University Medical Center, Homburg, Germany; Department of Neurology, Saarland University, Campus Homburg, Building 90, Kirrberger Straße, Homburg 66421, Germany; Department of Neurology, Saarland University Medical Center, Homburg, Germany; Department of Neurology, Saarland University, Campus Homburg, Building 90, Kirrberger Straße, Homburg 66421, Germany

**Keywords:** disability-adjusted life years (DALYs), Global Burden of Disease (GBD), years lived with disability (YLDs), years of life lost (YLLs)

## Abstract

**Introduction:**

The burden of ischaemic stroke in Europe has evolved over recent decades, shaped by changes in prevention, acute care and population ageing. Understanding long-term trends in mortality and disability is essential for guiding policy and service planning.

**Patients and methods:**

Using Global Burden of Disease (GBD) 2023 estimates, we analysed age-standardised disability-adjusted life years (DALYs), years of life lost (YLLs) and years lived with disability (YLDs) for ischaemic stroke across all European countries from 1990 to 2023. We estimated country-specific annual percentage changes (EAPCs), assessed non-linear trajectories, examined shifts in the fatal vs non-fatal composition of DALYs and synthesised trends using mixed-effects models and descriptive meta-analytic pooling.

**Results:**

Disability-adjusted life year rates declined in every European country, with the steepest reductions in Central and Western Europe and more modest improvements in parts of Eastern and Southeastern Europe. Years of life lost declines closely paralleled DALY trends, indicating that falling premature mortality was the main driver of the overall reduction. Years lived with disability trends were smaller and more heterogeneous across countries. Mixed-effects modelling indicated average European EAPCs of −3.40% (95% CI, −3.74 to −3.05) for DALYs, −1.26% (−1.43 to −1.09) for YLLs and −3.86% (−4.26 to −3.46) for YLDs. Meta-analytic pooling of national patterns showed large declines in YLLs (random-effects EAPC −3.81%) relative to YLDs (−0.86%).

**Discussion and conclusion:**

Ischaemic stroke burden has fallen substantially across Europe, driven primarily by reductions in premature mortality. Slower progress in disability outcomes and persistent regional disparities highlight the need to strengthen long-term rehabilitation and address uneven improvements across the continent.

## Graphical Abstract

**Graphical Abstract:**
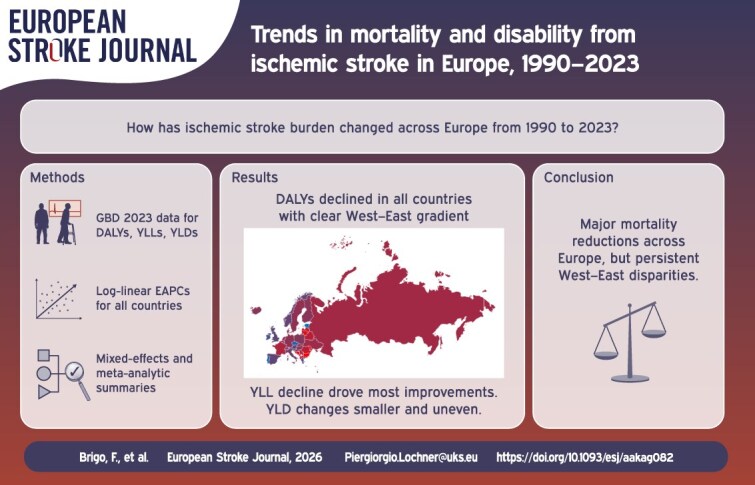

## Introduction

Across Europe, the landscape of ischaemic stroke has been reshaped by profound demographic, clinical and health-system changes over the past 3 decades. Populations have aged, vascular risk profiles have shifted and countries have adopted a wide range of prevention and treatment strategies with varying speed and intensity.^1–4^ These developments have altered not only the incidence and severity of stroke, but also the balance between survival and long-term disability. As a result, the overall burden of ischaemic stroke has evolved in ways that are unlikely to be captured by mortality statistics alone. These dynamics are consistent with long-term projections and historical trends described in European stroke burden analyses.^1^^,^^5^

Although many European countries have reported improvements in acute stroke care and secondary prevention, the extent to which these advances have translated into population-level reductions in disability and premature mortality remains uncertain.^6–8^ Existing international comparisons often focus on single outcomes or limited time windows, making it difficult to understand how the fatal and non-fatal components of stroke burden have changed relative to one another. Moreover, Europe encompasses substantial heterogeneity in socioeconomic conditions, health-system capacity and public-health investment, raising the possibility that long-term trends may diverge markedly across countries.^9–11^ Recent analyses confirm substantial regional disparities, particularly between Western and Eastern Europe, where incidence, mortality and DALY rates remain markedly higher.^12^

The Global Burden of Disease (GBD) 2023 study provides a consistent framework for evaluating these patterns.^13^ Its standardised methodology allows direct comparison of disability-adjusted life years (DALYs), years of life lost (YLLs) and years lived with disability (YLDs)^14^ across all European countries over more than 3 decades. Leveraging these data makes it possible to examine not only whether the burden of ischaemic stroke has declined, but also how the composition of that burden has shifted as survival improves and more people live with chronic sequelae. Comparable methodological approaches and findings have been documented in previous GBD-based ischaemic stroke assessments.^15^

In this study, we used GBD 2023 estimates to characterise long-term trends in ischaemic stroke burden across Europe from 1990 to 2023. We analysed changes in DALYs, YLLs and YLDs; evaluated the presence of non-linear trajectories; quantified shifts in the relative contribution of fatal and non-fatal outcomes and synthesised country-specific trends using mixed-effects modelling and meta-analytic approaches. By integrating these complementary perspectives, our aim was to provide an updated and comprehensive picture of how ischaemic stroke burden has evolved across Europe, and to identify persistent disparities and emerging challenges relevant to prevention, acute care and long-term recovery. The need for such integrated analyses is supported by prior Europe-wide evaluations highlighting major gaps in stroke care pathways and cross-country inequalities.^1^

## Patients and methods

### Data source and study population

We used publicly available estimates from the GBD 2023 study, extracting age-standardised rates of DALYs, YLLs and YLDs for ischaemic stroke for all European countries from 1990 to 2023. For each country, sex-specific annual estimates were retrieved. Only age-standardised rates were analysed to ensure comparability across countries and over time. No individual-level data were accessed. Because GBD estimates are modelled outputs rather than raw observations, all analyses were interpreted as descriptive characterisations of temporal patterns rather than formal inferential tests. Confidence intervals reflect model-based uncertainty rather than sampling variability.

### Data processing and construction of analytic variables

For each country–sex–year combination, we reshaped the dataset to obtain parallel annual series for DALYs, YLLs and YLDs. Log-transformed outcomes were generated to support estimation of temporal trends under a log-linear assumption. To examine changes in the composition of ischaemic stroke burden, we computed the proportional contribution of YLLs and YLDs to total DALYs and derived logit-transformed shares. A completeness audit verified the presence of all expected annual observations for each metric.

### Estimation of annual percentage change (EAPC)

Temporal trends were quantified using the estimated annual percentage change (EAPC).^16^ For each country, sex and outcome, we fitted ordinary least squares regressions of the form.

ln(rate) = β0 + β1(year−1990) + ε,

where the exponentiated slope coefficient provided the EAPC and its 95% CI. To screen for departures from linearity, we fitted an extended model including a quadratic term and assessed its joint significance. This assessment was used solely to flag potential non-linear patterns, was not intended to model them and does not identify structural breakpoints. To avoid over-interpreting statistically significant quadratic terms, we quantified the effect size of non-linearity by estimating, for each country, the difference between the linear slope (β₁) and the slope from a quadratic model (β₁ from a model including year and year^2^). Both slopes were converted into EAPCs, and the difference (ΔEAPC) was used as a measure of curvature magnitude.

### Mixed-effects modelling of the European average trend

To estimate the average European trend while accounting for between-country heterogeneity, we fitted linear mixed-effects models with random intercepts and random slopes for countries. The fixed slope represents the pooled European EAPC. In this framework, the fixed effect represents the continental average trend rather than the mean of national EAPCs while the random effects allow each country to deviate from this pattern in both baseline level and temporal trajectory. This approach leverages all annual observations simultaneously and stabilises estimates for countries with noisier or more variable time series. Country-specific deviations from the European trend were summarised using best linear unbiased predictors (BLUPs). Best linear unbiased predictors represent deviations from the European average and are predictions rather than estimable parameters; they are therefore not interpreted as country-specific EAPCs and are reported without CIs.

### Sensitivity analyses

We evaluated temporal changes in the composition of ischaemic stroke burden by regressing logit-transformed YLL and YLD shares on calendar year for each country and sex, to assess whether the relative contribution of fatal and non-fatal components changed over time. Additional sensitivity analyses included the exclusion of countries with populations < 1 million (ie, Monaco, San Marino, Andorra, Luxembourg, Malta and Iceland) to assess the stability of continental-level estimates. Finally, we compared the change in YLLs and YLDs between the first and last available year using an arithmetic decomposition, which quantified absolute and proportional contributions without applying structural decomposition methods.

### Meta-analysis of country-specific EAPCs

To provide a descriptive summary of country-level EAPCs, we reconstructed standard errors from CIs and performed fixed-effects and DerSimonian–Laird random-effects meta-analyses. Between-country heterogeneity was quantified using the Q statistic and *I*^2^. Because GBD estimates across countries are not statistically independent, pooled estimates were interpreted as descriptive summaries rather than inferential measures. Unlike the mixed-effects model described above, the meta-analysis does not model the underlying time series or assume a shared European trend; instead, it pools national EAPCs based solely on their point estimates and variances. Because European countries do not constitute a random sample and GBD-derived estimates are not statistically independent, pooled values were interpreted as descriptive summaries of the distribution of national EAPCs rather than as alternative estimates of the continental trend.

In addition, we summarised the empirical distribution of country-level EAPCs (median and IQR) to describe the observed heterogeneity without applying any pooling.

### Statistical analysis

All analyses were conducted using Stata 17 (StataCorp LLC, College Station, TX, USA). Data management, regression modelling and graphical outputs followed standard procedures. Statistical significance was defined as *P* < .05, and all tests were 2-sided.

### Ethical considerations

This study used publicly available, aggregated data from the GBD 2023 study.^13^ No individual-level or identifiable information was accessed at any stage of the analysis. In accordance with international guidelines for research using publicly accessible, de-identified datasets, formal ethical approval was not required.

## Results

**Figure 1:**
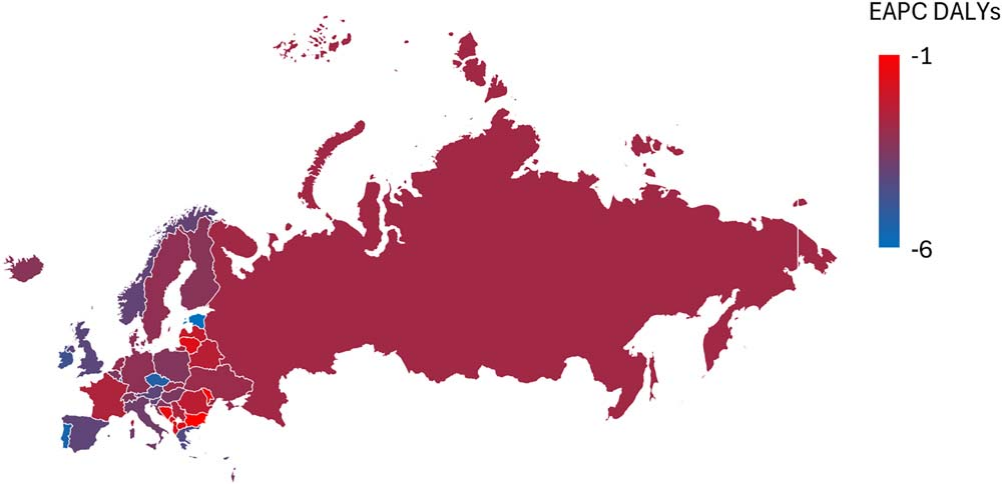
Estimated APC in DALYs due to ischaemic stroke across Europe, 1990–2023. Abbreviations: APC = annual percentage change; DALYs = disability-adjusted life years.

Age-standardised DALY rates for ischaemic stroke declined in all European countries between 1990 and 2023 (Table S1; Figure 1). The magnitude of improvement varied substantially: the steepest annual reductions were observed in Central and Western Europe, where declines often exceeded −4% per year. In contrast, several Eastern and Southeastern European countries experienced more modest improvements. Sex-specific patterns were broadly similar, although in parts of Eastern Europe men showed slightly smaller reductions than women.

Reductions in YLL rates closely mirrored those of DALYs (Figure S1). Years lived with disability trends were smaller and more heterogeneous (Figure S2). While most countries experienced modest decreases, several high-income Western European countries showed near-stable YLD rates. Conversely, countries such as Portugal, Luxembourg and the Czech Republic demonstrated more pronounced improvements in YLDs.

Baseline (1990) and end-period (2023) absolute DALY, YLL and YLD rates for each country (Table S2) show that several Western and Northern European countries, despite exhibiting smaller or stable YLD trends, started the study period with substantially lower disability and mortality levels than many Eastern and Southeastern countries. As a result, their relative improvements appear smaller even though their absolute burden remained among the lowest in Europe.

Although several countries showed statistically significant quadratic terms (*P* < .05), the effect-size analysis indicated that these deviations were small. Differences between linear and quadratic slopes were minor (typically –0.03 to +0.03), and no country showed a reversal of trend direction. On the epidemiological scale, changes in EAPC were modest: generally < 2 percentage points and usually < 1 percentage point (Table S3). Thus, despite statistical significance driven by the long time series, the curvature had negligible impact on long-term trend estimates.

Mixed-effects modelling revealed clear continent-wide declines across all three outcomes. Because this approach estimates the average European slope across all observations, it reflects the overall continental trajectory rather than the mean of national trends. Using this framework, the fixed-effect slope corresponded to an average European EAPC of −3.40% (95% CI, −3.74 to −3.05) for DALYs. For YLLs, the estimated continental trend was more modest, with an average EAPC of −1.26% (95% CI, −1.43 to −1.09), reflecting slower but consistent reductions in premature mortality. In contrast, YLDs showed a steeper average decline, with a mixed-effects EAPC of −3.86% (95% CI, −4.26 to −3.46), indicating substantial improvements in disability outcomes in many countries.

Best linear unbiased predictions of the estimated annual percentage change (BLUP-EAPCs) showed marked heterogeneity in country-specific deviations from the overall European trend, and a clear West–East gradient (Figures S3–SS5; Table S4). Western and Northern European countries generally exhibited faster-than-average declines in DALYs, YLDs and YLLs. In contrast, several Eastern and Southeastern European countries showed positive deviations across outcomes, indicating slower progress or relative increases.

The logit-share analysis showed a consistent and statistically significant shift in the composition of DALYs, with the proportion attributable to YLLs decreasing and the share attributable to YLDs increasing in every country (*P* < .001 in nearly all settings; Table S5). These findings indicate that improvements in survival have progressively increased the relative contribution of long-term disability to the total burden.

Decomposition analyses further confirmed that declines in DALYs were overwhelmingly driven by reductions in YLLs (Table S6). Countries with the largest DALY reductions also showed the largest decreases in YLLs, whereas countries with smaller improvements maintained the same mortality-dominated profile.

Sensitivity analyses excluding countries with populations < 1 million yielded similar results. Among the remaining 38 countries, the mean EAPC for DALYs was −3.29%, closely matching the full-sample estimate.

Meta-analytic pooling of country-specific EAPCs supported the robustness of these findings. This approach summarises the distribution of national trends rather than the overall continental trajectory estimated by the mixed-effects model (see above). For DALYs, the random-effects pooled EAPC was –3.37% (95% CI, –3.44 to –3.29) with substantial heterogeneity (*I*^2^ = 83.6%) (Figure S6). In meta-analytic summaries, YLLs showed even larger declines, with a random-effects pooled EAPC of –3.81% (95% CI, –3.89 to –3.74) and high heterogeneity (*I*^2^ = 78.4%) (Figure S7), highlighting the dominant role of mortality reductions in shaping the overall burden. Years lived with disability showed a much smaller decline, with a pooled estimate of –0.86% (95% CI, –0.88 to –0.85) and no detectable heterogeneity (*I*^2^ = 0%) (Figure S8).

To complement the descriptive meta-analysis, we summarised the variability in trends across Europe by examining the distribution of country-level EAPCs (both sexes). This analysis does not report individual country values but provides a statistical overview of how national trends are distributed. For DALYs, the median EAPC was –3.40% (IQR: –4.18% to –2.66%), indicating substantial heterogeneity in the magnitude of decline. Years lived with disability showed steeper and more variable reductions, with a median of –3.95% (IQR: –4.89% to –2.84%). In contrast, YLLs displayed smaller and more homogeneous declines, with a median of –1.26% (IQR: –1.62% to –0.90%).

## Discussion

This comprehensive assessment of ischaemic stroke burden across Europe reveals substantial but uneven progress over the past 3 decades. All countries experienced declines in age-standardised DALY rates between 1990 and 2023, yet the magnitude of improvement varied markedly. Western, Northern and parts of Central Europe achieved the steepest reductions, whereas several Eastern and Southeastern European countries showed more modest gains. Although these findings should be interpreted as descriptive of population-level temporal patterns rather than as evidence of causal relationships, these regional differences mirror long-standing disparities in cardio- and cerebrovascular risk-factor control, access to acute stroke care and the pace of health-system modernisation across the continent. Similar geographic gradients have been documented in prior European stroke epidemiology studies.^1^^,^^12^ Together, these patterns highlight a persistent and well-documented east–west divide in stroke outcomes across Europe.

Importantly, our study provides the most up-to-date assessment of ischaemic stroke burden in Europe using GBD 2023 data and offers new information not available in the few previous GBD-based analyses focusing on Europe.^1^^,^^12^^,^^17^ Unlike these earlier reports, we analyse YLL and YLD separately, allowing us to disentangle the fatal and non-fatal components of the burden and to characterise how their balance has evolved over time. This level of granularity, combined with the use of multiple complementary analytic approaches, provides a complementary, more detailed and contemporary picture of the changing burden of ischaemic stroke across Europe.

The dominant driver of the overall decline was the reduction in YLLs, reflecting major improvements in survival after ischaemic stroke. This pattern was consistent across countries and robust to multiple analytic approaches, including mixed-effects modelling and descriptive meta-analytic pooling. Hence, the magnitude and consistency of these reductions across countries suggest that the observed decline in mortality-related burden is unlikely to be explained solely by model uncertainty or data artefacts. The magnitude of YLL reductions was substantially larger than that of YLDs, reinforcing the central role of mortality declines in shaping overall DALY trends. The widespread decline in premature mortality likely reflects the combined impact of improved hypertension management, declining smoking prevalence in many countries, expanded availability of reperfusion therapies and strengthened stroke-care pathways.^18^^,^^19^ Evidence from European cohort and health-system studies supports the role of improved acute care and prevention in reducing stroke mortality.^7^^,^^15^^,^^20^

In contrast, trends in YLDs were smaller and more heterogeneous. Several high-income Western European countries exhibited near-stable disability rates, suggesting that gains in survival have not been matched by equivalent improvements in long-term functional outcomes. These near-stable trajectories may partly reflect the increasing survival of older, multimorbid patients who are more likely to experience long-term disability. This divergence may reflect increasing stroke survivorship among older adults with multimorbidity and increasing frailty, limited progress in post-acute rehabilitation capacity or widening gaps in access to community-based recovery services. Prior analyses have highlighted persistent limitations in rehabilitation availability and post-stroke support across Europe.^21^ Conversely, countries such as Portugal, Luxembourg and the Czech Republic showed more pronounced declines in YLDs, indicating that improvements in disability outcomes are achievable but not uniformly distributed.

Baseline values help contextualise these patterns. Several Western and Northern European countries began the 1990s with substantially lower DALY, YLL and YLD rates than many Eastern and Southeastern countries. Consequently, their more modest relative improvements—particularly for YLDs—reflect ceiling effects rather than stagnation. In contrast, countries with higher initial burden had greater scope for improvement, resulting in larger relative declines. This baseline context helps avoid misinterpreting stable YLD trajectories in high performing countries as lack of progress.

The logit-share analysis provides further insight into these dynamics, showing a consistent shift towards a greater proportion of DALYs attributable to YLDs. As mortality declines, the relative weight of long-term disability inevitably increases, underscoring the need for health systems to adapt to a growing population of stroke survivors requiring sustained rehabilitation and chronic care. This shift has important implications for resource allocation, workforce planning and the design of integrated stroke-recovery pathways. Similar transitions towards a disability-heavy burden have been noted in other high-income settings.^15^^,^^22^ This transition underscores the growing importance of long-term recovery pathways as survival improves.

Non-linear temporal patterns observed in several countries suggest that progress has not been uniform over time. Periods of accelerated improvement may correspond to major policy reforms, expansion of stroke units or rapid adoption of evidence-based therapies, whereas periods of stagnation may reflect economic crises, health-system disruptions or plateaus in risk-factor control. Despite these fluctuations, all countries maintained negative overall EAPCs, reinforcing the robustness of the long-term downward trend. Given the long time series (34 years), even modest curvature was statistically detectable. However, these deviations were small in magnitude and did not change the sign of the estimated slopes, indicating a consistently downward long-term trend in ischaemic stroke metrics. Historical analyses of European stroke systems similarly show that improvements often occur in waves linked to policy and technological milestones.^7^

Sensitivity analyses confirmed the stability of the findings. Excluding countries with populations < 1 million did not materially alter the continental trend, and descriptive meta-analytic pooling produced estimates closely aligned with the unweighted mean, indicating that no single country or subgroup disproportionately influenced the results.

The difference between the mixed-effects estimate of the European trend (EAPC −1.26%) and the meta-analytic summary of country-specific EAPCs (EAPC −3.81%) arises because the 2 approaches quantify distinct epidemiological quantities rather than alternative estimates of the same parameter. The mixed-effects model reconstructs a single continental trajectory by drawing on all annual observations and applying partial pooling, which reduces the influence of extreme or unstable national series. This produces a smoothed and conservative estimate of the average European decline in YLLs, reflecting the overall shape of the continental time trend rather than the arithmetic mean of national slopes. The meta-analysis, by contrast, summarises the distribution of country-specific EAPCs without modelling the underlying time series and without imposing a shared European trend. Many countries, particularly in Western and Central Europe, exhibit steep annual reductions in YLLs, and these strong national declines drive the pooled estimate towards a more negative value. The meta-analytic result therefore captures the magnitude of improvement observed across individual countries rather than the continent-wide trajectory estimated by the mixed-effects model. These 2 perspectives are complementary rather than contradictory. The mixed-effects model answers the question of how the European burden is evolving as a whole, whereas the meta-analysis describes how strongly individual countries are improving. Their convergence in direction reinforces the robustness of the findings, while their quantitative differences highlight the value of triangulating multiple analytic frameworks when interpreting geographically structured epidemiological trends.

Our findings of persistent regional disparities in ischaemic stroke burden underscore the critical importance of the Stroke Action Plan for Europe (SAP-E) 2018–2030.^22^ While mortality (YLL) is falling across the continent, the plateauing disability rates (YLD) and the gaps in South-eastern Europe highlight that “homogenising” care is not just a clinical goal but a demographic necessity and a target of equity for European citizens. The SAP-E targets, such as ensuring that 90% of patients are treated in dedicated stroke units and establishing national plans for “life after stroke,” are essential to translate gains in life expectancy into gains in healthy life years.^22^ More specifically, European countries would benefit from systematic screening for “silent” risk factors—particularly hypertension, atrial fibrillation, dyslipidaemia and diabetes—at the primary-care level, in line with recent evidence on combination antihypertensive therapy and updated European Society of Cardiology (ESC) guidelines for atrial fibrillation management,^23^^,^^24^ and management of dyslipidaemia.^25^ In parallel, improving diabetes control through modern cardioprotective agents may further reduce stroke risk.^26^ Although comparable longitudinal SAP-E indicators were not available to formally model their contribution, differences in stroke-unit coverage, reperfusion rates and post-acute care capacity likely account for part of the heterogeneity observed across countries. Their wider adoption in the coming years may help narrow the regional disparities in ischaemic stroke burden.

Beyond prevention, bridging the gap in acute and post-acute care remains essential. As an example, the German Stroke Unit certification model provides a benchmark for high-quality acute care^27^; similarly, the European Stroke Organisation (ESO) Stroke Unit Certification program aims to improve quality and to overcome the wide discrepancies in stroke care both within and between European countries.^28^ The development of a new European aim focused specifically on rehabilitation quality represents a critical step towards harmonising standards across Europe. Establishing minimum educational criteria for “stroke nurses” and “stroke therapists” across the European Union (EU) could further enhance professional mobility and ensure consistent care quality.

This study has several limitations. First, it relies entirely on GBD 2023 estimates,^13^ which are modelled rather than directly measured and therefore depend on the quality and completeness of underlying data sources. Countries with limited vital-registration or hospital-discharge data may have greater uncertainty in their estimates, particularly for YLDs, which rely on disability weights and epidemiological modelling rather than systematic clinical follow-up. Confidence intervals therefore reflect model-based uncertainty rather than sampling variability. Furthermore, the smaller and more heterogeneous changes observed in YLDs may also reflect greater uncertainty in disability estimation compared with mortality data, as well as differences in case ascertainment and long-term follow-up across countries. Second, the use of age-standardised rates facilitates comparability but may obscure important within-country differences by age group or socioeconomic status. Third, the EAPC framework assumes log-linear trends; although we tested for non-linearity, short-term fluctuations or abrupt policy-driven changes may not be fully captured. Furthermore, pooled estimates should be interpreted as descriptive summaries of cross-country patterns rather than formal inferential measures, given the lack of statistical independence between GBD-derived estimates. Fourth, the analysis does not incorporate incidence or case-fatality trends directly, limiting the ability to disentangle whether improvements arise from prevention, acute care or post-acute management. This reflects the scope of the study, which was designed to characterise long-term patterns in burden rather than to disentangle the underlying epidemiological drivers. Hence, the absence of harmonised incidence data limits the ability to determine whether the combination of declining DALYs and relatively stable YLDs reflects primarily improved survival among older, multimorbid patients or whether it may also be influenced by other factors, such as rising stroke incidence in younger adults. Furthermore, although our study was not designed to perform forecast analyses with projection models, historical trends up to 2023 remain essential for anticipating future healthcare needs, as they reveal structural patterns—such as declining mortality and stable disability—that directly inform planning for long-term care and rehabilitation capacity. Finally, GBD estimates do not reflect uncertainty in health-system performance indicators, risk-factor trajectories or rehabilitation capacity, which may contribute to the observed regional differences.

Despite these limitations, our study has notable strengths. It provides the most up-to-date and comprehensive assessment of ischaemic stroke burden across all European countries using a standardised and internally consistent data source. By jointly analysing DALYs, YLLs and YLDs over more than 3 decades, the study captures both fatal and non-fatal components of the burden and their evolving balance. The use of mixed-effects modelling allowed us to estimate a continental average trend while simultaneously accounting for country-specific baseline levels and temporal trajectories, an approach well suited to structured geographic data where countries are not statistically independent. In parallel, the descriptive meta-analysis of country-specific EAPCs provided a complementary summary of national trends, offering an additional perspective on the distribution of changes across Europe. The use of multiple complementary analytic approaches therefore offers a robust and multidimensional characterisation of long-term trends. The inclusion of sensitivity analyses, such as the exclusion of small-population countries and the evaluation of non-linear trajectories, further strengthens the reliability of the findings. This triangulation across multiple analytic approaches strengthens confidence in the robustness of the observed continental trends. Together, these features provide a detailed and nuanced picture of how ischaemic stroke burden has changed across Europe and highlight persistent regional disparities that warrant targeted policy attention.

Importantly, this study provides detailed, country-specific metrics for DALYs, YLDs and YLLs across all European nations, offering a granular evidence base that can directly support national and regional public-health planning, policy prioritisation and performance assessment activities.

## Conclusion

Based on modelled estimates from the GBD 2023 Study, across Europe, the burden of ischaemic stroke has declined substantially over the past 3 decades, driven primarily by marked reductions in premature mortality. These gains were widespread but uneven, with Western and Northern Europe achieving the most rapid improvements and several Eastern and Southeastern countries showing slower progress. Disability-related outcomes improved more modestly and in many settings remained relatively stable, underscoring the growing need to strengthen long-term rehabilitation and chronic care for stroke survivors. While strengthening rehabilitation services is essential, acute stroke care remains a key determinant of both mortality and disability. Healthcare priorities differ substantially across European regions, and policies should be tailored to each country’s specific needs, including risk-factor control, acute care capacity and long-term recovery pathways.

The increasing proportion of DALYs attributable to YLDs likely reflects the success of prevention and acute care in reducing fatal events, while highlighting the rising importance of post-stroke disability as a public health challenge. Persistent regional disparities, non-linear trajectories in several countries and the limited decline in YLDs indicate that further progress will require targeted investment in both prevention and recovery pathways. Sustained efforts to improve risk-factor control, expand access to high-quality acute care and strengthen rehabilitation services will be essential to reduce the long-term burden of ischaemic stroke across Europe.

## Supplementary Material

Supplementary_material_aakag082

## Data Availability

All data used in this study were obtained from the GBD 2023 study, produced by the Institute for Health Metrics and Evaluation (IHME). The GBD 2023 estimates are publicly accessible through the Global Health Data Exchange (GHDx) and can be freely downloaded for non-commercial use via the GBD Results Tool and associated data resources.
