# Supplementary material for: Trends in mortality and disability from ischaemic stroke in Europe, 1990-2023
Source: Eur Stroke J. 2026 Jul 21;11(7):aakag082. doi: 10.1093/esj/aakag082 (PMC13387428; doi:10.1093/esj/aakag082)
Supplement: Supplementary_material_aakag082 [file supplementary_material_aakag082.zip › Supplementary Table 3.docx]

**Supplementary Table 3.** Effect Size of Non‑Linearity in Ischemic Stroke Trends: Difference Between Linear and Quadratic Slopes and EAPCs by Country, Sex, and Outcome (Europe, 1990–2023)

*β,lin is the slope of the log‑linear model; β,quad is the slope of the quadratic model including year²; diff_β is the difference between the two slopes (β,quad − β,lin). EAPC,lin and EAPC,quad are the Estimated Annual Percentage Changes derived from the linear and quadratic models, respectively; diff_EAPC quantifies the effect size of non‑linearity on the epidemiological scale. r² is the coefficient of determination of the linear model, and p‑value refers to the significance of the quadratic term. Positive diff_EAPC values indicate that the quadratic model yields a less negative trend than the linear model; negative values indicate a steeper decline.*

| **Measure** | **Sex** | **Country** | **β,lin** | **β,quad** | **diff_β** | **EAPC,lin** | **EAPC,quad** | **diff_EAPC** | **r2** | **p-value** |
| --- | --- | --- | --- | --- | --- | --- | --- | --- | --- | --- |
| DALYs | Both | Albania | -0,019 | -0,011 | 0,008 | -1,839 | -1,084 | 0,755 | 0,96 | 0,005 |
| DALYs | Both | Andorra | -0,029 | -0,042 | -0,014 | -2,838 | -4,152 | -1,314 | 0,98 | <0,0001 |
| DALYs | Both | Austria | -0,043 | -0,071 | -0,028 | -4,182 | -6,844 | -2,662 | 0,96 | <0,0001 |
| DALYs | Both | Belarus | -0,025 | 0,005 | 0,030 | -2,468 | 0,549 | 3,017 | 0,85 | <0,0001 |
| DALYs | Both | Belgium | -0,036 | -0,050 | -0,014 | -3,575 | -4,904 | -1,329 | 0,99 | <0,0001 |
| DALYs | Both | Bosnia and Herzegovina | -0,015 | -0,003 | 0,012 | -1,522 | -0,307 | 1,216 | 0,93 | <0,0001 |
| DALYs | Both | Bulgaria | -0,011 | -0,008 | 0,003 | -1,072 | -0,773 | 0,298 | 0,96 | 0,077 |
| DALYs | Both | Croatia | -0,035 | -0,012 | 0,023 | -3,434 | -1,203 | 2,231 | 0,97 | <0,0001 |
| DALYs | Both | Cyprus | -0,044 | -0,054 | -0,010 | -4,309 | -5,219 | -0,910 | 0,98 | 0,017 |
| DALYs | Both | Czechia | -0,055 | -0,042 | 0,013 | -5,350 | -4,113 | 1,237 | 0,98 | 0,019 |
| DALYs | Both | Denmark | -0,034 | -0,023 | 0,011 | -3,311 | -2,264 | 1,047 | 0,97 | 0,008 |
| DALYs | Both | Estonia | -0,062 | -0,056 | 0,006 | -6,043 | -5,434 | 0,609 | 0,93 | 0,614 |
| DALYs | Both | Finland | -0,034 | -0,047 | -0,013 | -3,371 | -4,598 | -1,227 | 0,99 | <0,0001 |
| DALYs | Both | France | -0,027 | -0,039 | -0,012 | -2,648 | -3,816 | -1,168 | 0,98 | <0,0001 |
| DALYs | Both | Germany | -0,033 | -0,063 | -0,030 | -3,253 | -6,112 | -2,859 | 0,94 | <0,0001 |
| DALYs | Both | Greece | -0,045 | -0,044 | 0,001 | -4,403 | -4,332 | 0,070 | 0,97 | 0,901 |
| DALYs | Both | Hungary | -0,037 | -0,040 | -0,004 | -3,587 | -3,938 | -0,351 | 0,97 | 0,387 |
| DALYs | Both | Iceland | -0,034 | -0,033 | 0,002 | -3,367 | -3,208 | 0,159 | 0,98 | 0,596 |
| DALYs | Both | Ireland | -0,049 | -0,053 | -0,004 | -4,766 | -5,146 | -0,379 | 0,99 | 0,323 |
| DALYs | Both | Israel | -0,037 | -0,039 | -0,002 | -3,652 | -3,860 | -0,208 | 0,99 | 0,516 |
| DALYs | Both | Italy | -0,039 | -0,064 | -0,025 | -3,825 | -6,172 | -2,347 | 0,97 | <0,0001 |
| DALYs | Both | Latvia | -0,022 | -0,017 | 0,005 | -2,203 | -1,718 | 0,485 | 0,92 | 0,325 |
| DALYs | Both | Lithuania | -0,017 | 0,008 | 0,025 | -1,720 | 0,788 | 2,507 | 0,84 | <0,0001 |
| DALYs | Both | Luxembourg | -0,053 | -0,069 | -0,016 | -5,204 | -6,667 | -1,463 | 0,99 | <0,0001 |
| DALYs | Both | Malta | -0,045 | -0,038 | 0,007 | -4,399 | -3,742 | 0,657 | 0,99 | 0,040 |
| DALYs | Both | Monaco | -0,046 | -0,008 | 0,038 | -4,484 | -0,813 | 3,672 | 0,95 | <0,0001 |
| DALYs | Both | Montenegro | -0,019 | 0,010 | 0,029 | -1,898 | 0,981 | 2,880 | 0,84 | <0,0001 |
| DALYs | Both | Netherlands | -0,031 | -0,035 | -0,004 | -3,089 | -3,432 | -0,343 | 0,96 | 0,438 |
| DALYs | Both | North Macedonia | -0,018 | 0,007 | 0,025 | -1,754 | 0,701 | 2,455 | 0,87 | <0,0001 |
| DALYs | Both | Norway | -0,042 | -0,041 | 0,001 | -4,100 | -3,976 | 0,124 | 0,99 | 0,693 |
| DALYs | Both | Poland | -0,036 | -0,039 | -0,003 | -3,491 | -3,779 | -0,288 | 0,99 | 0,196 |
| DALYs | Both | Portugal | -0,057 | -0,069 | -0,012 | -5,555 | -6,694 | -1,140 | 0,98 | 0,018 |
| DALYs | Both | Republic of Moldova | -0,012 | 0,018 | 0,029 | -1,174 | 1,772 | 2,945 | 0,43 | <0,0001 |
| DALYs | Both | Romania | -0,024 | -0,001 | 0,023 | -2,377 | -0,059 | 2,317 | 0,91 | <0,0001 |
| DALYs | Both | Russian Federation | -0,029 | 0,009 | 0,038 | -2,893 | 0,892 | 3,785 | 0,83 | <0,0001 |
| DALYs | Both | San Marino | -0,040 | -0,049 | -0,009 | -3,935 | -4,782 | -0,846 | 0,99 | 0,012 |
| DALYs | Both | Serbia | -0,027 | -0,011 | 0,016 | -2,695 | -1,142 | 1,553 | 0,96 | <0,0001 |
| DALYs | Both | Slovakia | -0,031 | -0,016 | 0,015 | -3,031 | -1,565 | 1,466 | 0,97 | <0,0001 |
| DALYs | Both | Slovenia | -0,042 | -0,063 | -0,021 | -4,153 | -6,136 | -1,982 | 0,98 | <0,0001 |
| DALYs | Both | Spain | -0,043 | -0,070 | -0,027 | -4,180 | -6,765 | -2,584 | 0,97 | <0,0001 |
| DALYs | Both | Sweden | -0,033 | -0,025 | 0,008 | -3,211 | -2,459 | 0,752 | 0,99 | 0,005 |
| DALYs | Both | Switzerland | -0,036 | -0,053 | -0,017 | -3,518 | -5,180 | -1,661 | 0,98 | <0,0001 |
| DALYs | Both | Ukraine | -0,031 | -0,019 | 0,012 | -3,027 | -1,904 | 1,123 | 0,97 | 0,003 |
| DALYs | Both | United Kingdom | -0,043 | -0,045 | -0,002 | -4,243 | -4,389 | -0,146 | 0,98 | 0,774 |
| DALYs | Female | Albania | -0,018 | -0,010 | 0,008 | -1,754 | -1,000 | 0,754 | 0,95 | 0,008 |
| DALYs | Female | Andorra | -0,029 | -0,045 | -0,016 | -2,861 | -4,442 | -1,581 | 0,97 | <0,0001 |
| DALYs | Female | Austria | -0,043 | -0,073 | -0,029 | -4,250 | -7,018 | -2,768 | 0,96 | <0,0001 |
| DALYs | Female | Belarus | -0,026 | 0,002 | 0,027 | -2,532 | 0,155 | 2,687 | 0,88 | <0,0001 |
| DALYs | Female | Belgium | -0,036 | -0,049 | -0,013 | -3,579 | -4,795 | -1,216 | 0,99 | <0,0001 |
| DALYs | Female | Bosnia and Herzegovina | -0,017 | -0,004 | 0,013 | -1,674 | -0,432 | 1,241 | 0,94 | <0,0001 |
| DALYs | Female | Bulgaria | -0,013 | -0,007 | 0,006 | -1,271 | -0,685 | 0,585 | 0,96 | <0,0001 |
| DALYs | Female | Croatia | -0,037 | -0,014 | 0,023 | -3,592 | -1,378 | 2,214 | 0,97 | <0,0001 |
| DALYs | Female | Cyprus | -0,045 | -0,056 | -0,011 | -4,419 | -5,492 | -1,073 | 0,98 | 0,006 |
| DALYs | Female | Czechia | -0,056 | -0,038 | 0,018 | -5,453 | -3,733 | 1,720 | 0,98 | 0,003 |
| DALYs | Female | Denmark | -0,034 | -0,024 | 0,010 | -3,344 | -2,352 | 0,992 | 0,98 | 0,007 |
| DALYs | Female | Estonia | -0,069 | -0,059 | 0,010 | -6,676 | -5,776 | 0,900 | 0,94 | 0,467 |
| DALYs | Female | Finland | -0,034 | -0,048 | -0,014 | -3,308 | -4,690 | -1,382 | 0,98 | <0,0001 |
| DALYs | Female | France | -0,027 | -0,037 | -0,011 | -2,621 | -3,671 | -1,050 | 0,98 | <0,0001 |
| DALYs | Female | Germany | -0,034 | -0,062 | -0,027 | -3,391 | -5,994 | -2,603 | 0,95 | <0,0001 |
| DALYs | Female | Greece | -0,048 | -0,044 | 0,004 | -4,660 | -4,278 | 0,382 | 0,97 | 0,526 |
| DALYs | Female | Hungary | -0,038 | -0,042 | -0,004 | -3,746 | -4,161 | -0,414 | 0,98 | 0,299 |
| DALYs | Female | Iceland | -0,031 | -0,027 | 0,004 | -3,082 | -2,680 | 0,403 | 0,97 | 0,268 |
| DALYs | Female | Ireland | -0,048 | -0,050 | -0,002 | -4,691 | -4,880 | -0,189 | 0,99 | 0,607 |
| DALYs | Female | Israel | -0,037 | -0,039 | -0,002 | -3,654 | -3,828 | -0,174 | 0,99 | 0,556 |
| DALYs | Female | Italy | -0,039 | -0,062 | -0,023 | -3,780 | -5,983 | -2,203 | 0,97 | <0,0001 |
| DALYs | Female | Latvia | -0,025 | -0,019 | 0,006 | -2,480 | -1,923 | 0,557 | 0,93 | 0,285 |
| DALYs | Female | Lithuania | -0,022 | 0,007 | 0,029 | -2,218 | 0,699 | 2,917 | 0,87 | <0,0001 |
| DALYs | Female | Luxembourg | -0,054 | -0,067 | -0,013 | -5,291 | -6,504 | -1,213 | 0,99 | 0,006 |
| DALYs | Female | Malta | -0,047 | -0,037 | 0,010 | -4,575 | -3,614 | 0,961 | 0,98 | 0,022 |
| DALYs | Female | Monaco | -0,043 | -0,015 | 0,029 | -4,256 | -1,466 | 2,790 | 0,97 | <0,0001 |
| DALYs | Female | Montenegro | -0,019 | 0,009 | 0,029 | -1,900 | 0,954 | 2,854 | 0,84 | <0,0001 |
| DALYs | Female | Netherlands | -0,031 | -0,031 | 0,000 | -3,057 | -3,043 | 0,014 | 0,97 | 0,974 |
| DALYs | Female | North Macedonia | -0,018 | 0,009 | 0,028 | -1,797 | 0,952 | 2,749 | 0,85 | <0,0001 |
| DALYs | Female | Norway | -0,042 | -0,042 | 0,000 | -4,123 | -4,114 | 0,009 | 0,99 | 0,980 |
| DALYs | Female | Poland | -0,040 | -0,042 | -0,002 | -3,889 | -4,113 | -0,224 | 0,99 | 0,355 |
| DALYs | Female | Portugal | -0,058 | -0,064 | -0,006 | -5,661 | -6,225 | -0,564 | 0,99 | 0,224 |
| DALYs | Female | Republic of Moldova | -0,017 | 0,010 | 0,027 | -1,689 | 1,016 | 2,705 | 0,59 | <0,0001 |
| DALYs | Female | Romania | -0,026 | -0,003 | 0,024 | -2,599 | -0,269 | 2,330 | 0,93 | <0,0001 |
| DALYs | Female | Russian Federation | -0,032 | 0,005 | 0,038 | -3,194 | 0,548 | 3,742 | 0,86 | <0,0001 |
| DALYs | Female | San Marino | -0,036 | -0,046 | -0,010 | -3,562 | -4,514 | -0,951 | 0,98 | 0,003 |
| DALYs | Female | Serbia | -0,028 | -0,012 | 0,016 | -2,759 | -1,146 | 1,613 | 0,96 | <0,0001 |
| DALYs | Female | Slovakia | -0,034 | -0,020 | 0,014 | -3,329 | -1,972 | 1,358 | 0,98 | <0,0001 |
| DALYs | Female | Slovenia | -0,044 | -0,061 | -0,017 | -4,302 | -5,906 | -1,604 | 0,99 | <0,0001 |
| DALYs | Female | Spain | -0,047 | -0,073 | -0,025 | -4,610 | -7,008 | -2,398 | 0,98 | <0,0001 |
| DALYs | Female | Sweden | -0,033 | -0,020 | 0,012 | -3,207 | -2,028 | 1,179 | 0,98 | <0,0001 |
| DALYs | Female | Switzerland | -0,035 | -0,052 | -0,016 | -3,479 | -5,046 | -1,566 | 0,98 | <0,0001 |
| DALYs | Female | Ukraine | -0,033 | -0,021 | 0,013 | -3,251 | -2,031 | 1,220 | 0,97 | <0,0001 |
| DALYs | Female | United Kingdom | -0,043 | -0,042 | 0,001 | -4,179 | -4,126 | 0,053 | 0,98 | 0,916 |
| DALYs | Male | Albania | -0,019 | -0,011 | 0,008 | -1,906 | -1,140 | 0,767 | 0,96 | 0,006 |
| DALYs | Male | Andorra | -0,029 | -0,040 | -0,011 | -2,812 | -3,877 | -1,066 | 0,98 | <0,0001 |
| DALYs | Male | Austria | -0,044 | -0,071 | -0,027 | -4,286 | -6,813 | -2,527 | 0,96 | <0,0001 |
| DALYs | Male | Belarus | -0,024 | 0,007 | 0,031 | -2,394 | 0,691 | 3,085 | 0,84 | <0,0001 |
| DALYs | Male | Belgium | -0,038 | -0,052 | -0,015 | -3,685 | -5,096 | -1,411 | 0,99 | <0,0001 |
| DALYs | Male | Bosnia and Herzegovina | -0,014 | -0,002 | 0,012 | -1,391 | -0,181 | 1,210 | 0,90 | <0,0001 |
| DALYs | Male | Bulgaria | -0,009 | -0,008 | 0,001 | -0,896 | -0,797 | 0,099 | 0,93 | 0,608 |
| DALYs | Male | Croatia | -0,035 | -0,011 | 0,024 | -3,406 | -1,066 | 2,340 | 0,96 | <0,0001 |
| DALYs | Male | Cyprus | -0,043 | -0,050 | -0,008 | -4,201 | -4,919 | -0,718 | 0,98 | 0,069 |
| DALYs | Male | Czechia | -0,055 | -0,048 | 0,007 | -5,378 | -4,679 | 0,699 | 0,98 | 0,160 |
| DALYs | Male | Denmark | -0,034 | -0,022 | 0,012 | -3,382 | -2,199 | 1,183 | 0,97 | 0,007 |
| DALYs | Male | Estonia | -0,056 | -0,051 | 0,004 | -5,404 | -4,981 | 0,423 | 0,93 | 0,705 |
| DALYs | Male | Finland | -0,036 | -0,048 | -0,012 | -3,560 | -4,691 | -1,131 | 0,99 | <0,0001 |
| DALYs | Male | France | -0,029 | -0,042 | -0,014 | -2,821 | -4,128 | -1,307 | 0,98 | <0,0001 |
| DALYs | Male | Germany | -0,034 | -0,068 | -0,034 | -3,345 | -6,574 | -3,229 | 0,93 | <0,0001 |
| DALYs | Male | Greece | -0,042 | -0,046 | -0,004 | -4,114 | -4,464 | -0,350 | 0,97 | 0,508 |
| DALYs | Male | Hungary | -0,035 | -0,036 | -0,001 | -3,456 | -3,540 | -0,084 | 0,97 | 0,839 |
| DALYs | Male | Iceland | -0,038 | -0,038 | 0,000 | -3,719 | -3,702 | 0,018 | 0,99 | 0,950 |
| DALYs | Male | Ireland | -0,050 | -0,056 | -0,006 | -4,907 | -5,459 | -0,553 | 0,99 | 0,195 |
| DALYs | Male | Israel | -0,038 | -0,040 | -0,002 | -3,703 | -3,921 | -0,218 | 0,99 | 0,546 |
| DALYs | Male | Italy | -0,041 | -0,066 | -0,025 | -3,994 | -6,345 | -2,351 | 0,97 | <0,0001 |
| DALYs | Male | Latvia | -0,020 | -0,016 | 0,004 | -1,993 | -1,584 | 0,409 | 0,92 | 0,384 |
| DALYs | Male | Lithuania | -0,012 | 0,010 | 0,022 | -1,211 | 0,955 | 2,166 | 0,77 | <0,0001 |
| DALYs | Male | Luxembourg | -0,053 | -0,070 | -0,017 | -5,205 | -6,791 | -1,586 | 0,99 | <0,0001 |
| DALYs | Male | Malta | -0,044 | -0,039 | 0,005 | -4,260 | -3,802 | 0,458 | 0,99 | 0,073 |
| DALYs | Male | Monaco | -0,050 | -0,002 | 0,048 | -4,832 | -0,151 | 4,681 | 0,94 | <0,0001 |
| DALYs | Male | Montenegro | -0,019 | 0,009 | 0,028 | -1,918 | 0,878 | 2,796 | 0,85 | <0,0001 |
| DALYs | Male | Netherlands | -0,033 | -0,042 | -0,009 | -3,251 | -4,091 | -0,840 | 0,96 | 0,089 |
| DALYs | Male | North Macedonia | -0,017 | 0,004 | 0,021 | -1,732 | 0,394 | 2,126 | 0,90 | <0,0001 |
| DALYs | Male | Norway | -0,043 | -0,039 | 0,003 | -4,186 | -3,861 | 0,324 | 0,99 | 0,263 |
| DALYs | Male | Poland | -0,032 | -0,035 | -0,003 | -3,180 | -3,423 | -0,244 | 0,99 | 0,249 |
| DALYs | Male | Portugal | -0,056 | -0,075 | -0,019 | -5,487 | -7,267 | -1,780 | 0,98 | 0,001 |
| DALYs | Male | Republic of Moldova | -0,007 | 0,027 | 0,034 | -0,681 | 2,722 | 3,403 | 0,21 | <0,0001 |
| DALYs | Male | Romania | -0,022 | 0,002 | 0,023 | -2,134 | 0,169 | 2,303 | 0,89 | <0,0001 |
| DALYs | Male | Russian Federation | -0,027 | 0,010 | 0,038 | -2,705 | 1,051 | 3,755 | 0,81 | <0,0001 |
| DALYs | Male | San Marino | -0,045 | -0,053 | -0,008 | -4,390 | -5,151 | -0,760 | 0,99 | 0,040 |
| DALYs | Male | Serbia | -0,027 | -0,012 | 0,016 | -2,667 | -1,146 | 1,521 | 0,96 | <0,0001 |
| DALYs | Male | Slovakia | -0,028 | -0,010 | 0,018 | -2,808 | -1,022 | 1,786 | 0,96 | <0,0001 |
| DALYs | Male | Slovenia | -0,043 | -0,067 | -0,024 | -4,208 | -6,450 | -2,242 | 0,98 | <0,0001 |
| DALYs | Male | Spain | -0,039 | -0,068 | -0,029 | -3,836 | -6,549 | -2,713 | 0,96 | <0,0001 |
| DALYs | Male | Sweden | -0,033 | -0,029 | 0,004 | -3,284 | -2,870 | 0,413 | 0,99 | 0,122 |
| DALYs | Male | Switzerland | -0,037 | -0,054 | -0,017 | -3,672 | -5,293 | -1,621 | 0,98 | <0,0001 |
| DALYs | Male | Ukraine | -0,029 | -0,021 | 0,008 | -2,842 | -2,072 | 0,770 | 0,97 | 0,048 |
| DALYs | Male | United Kingdom | -0,045 | -0,048 | -0,004 | -4,366 | -4,720 | -0,354 | 0,98 | 0,494 |
| YLDs | Both | Albania | -0,010 | -0,004 | 0,006 | -0,982 | -0,372 | 0,610 | 0,94 | <0,0001 |
| YLDs | Both | Andorra | -0,016 | -0,016 | 0,000 | -1,565 | -1,541 | 0,024 | 0,97 | 0,904 |
| YLDs | Both | Austria | -0,002 | 0,018 | 0,020 | -0,205 | 1,825 | 2,030 | 0,10 | <0,0001 |
| YLDs | Both | Belarus | -0,011 | -0,001 | 0,010 | -1,096 | -0,118 | 0,978 | 0,89 | <0,0001 |
| YLDs | Both | Belgium | -0,012 | -0,012 | 0,000 | -1,195 | -1,227 | -0,032 | 0,98 | 0,797 |
| YLDs | Both | Bosnia and Herzegovina | -0,010 | -0,003 | 0,007 | -0,991 | -0,287 | 0,703 | 0,93 | <0,0001 |
| YLDs | Both | Bulgaria | -0,007 | -0,005 | 0,002 | -0,669 | -0,496 | 0,173 | 0,97 | 0,070 |
| YLDs | Both | Croatia | -0,012 | -0,002 | 0,010 | -1,194 | -0,183 | 1,011 | 0,92 | <0,0001 |
| YLDs | Both | Cyprus | -0,013 | -0,027 | -0,014 | -1,293 | -2,690 | -1,397 | 0,87 | <0,0001 |
| YLDs | Both | Czechia | -0,020 | -0,003 | 0,016 | -1,937 | -0,307 | 1,630 | 0,93 | <0,0001 |
| YLDs | Both | Denmark | -0,016 | -0,001 | 0,015 | -1,576 | -0,111 | 1,465 | 0,92 | <0,0001 |
| YLDs | Both | Estonia | -0,017 | -0,006 | 0,011 | -1,731 | -0,621 | 1,110 | 0,94 | <0,0001 |
| YLDs | Both | Finland | -0,013 | 0,007 | 0,020 | -1,254 | 0,698 | 1,953 | 0,81 | <0,0001 |
| YLDs | Both | France | 0,001 | 0,006 | 0,005 | 0,067 | 0,587 | 0,519 | 0,17 | <0,0001 |
| YLDs | Both | Germany | -0,003 | -0,001 | 0,002 | -0,307 | -0,122 | 0,185 | 0,95 | <0,0001 |
| YLDs | Both | Greece | -0,017 | -0,015 | 0,002 | -1,639 | -1,475 | 0,164 | 0,98 | 0,314 |
| YLDs | Both | Hungary | -0,018 | -0,005 | 0,013 | -1,765 | -0,525 | 1,241 | 0,93 | <0,0001 |
| YLDs | Both | Iceland | -0,014 | -0,011 | 0,003 | -1,419 | -1,123 | 0,296 | 0,97 | 0,048 |
| YLDs | Both | Ireland | -0,020 | -0,029 | -0,009 | -1,987 | -2,867 | -0,880 | 0,96 | 0,002 |
| YLDs | Both | Israel | -0,017 | 0,003 | 0,020 | -1,672 | 0,328 | 2,000 | 0,88 | <0,0001 |
| YLDs | Both | Italy | -0,009 | -0,012 | -0,003 | -0,887 | -1,193 | -0,306 | 0,97 | 0,004 |
| YLDs | Both | Latvia | -0,012 | -0,004 | 0,008 | -1,240 | -0,398 | 0,842 | 0,94 | <0,0001 |
| YLDs | Both | Lithuania | -0,008 | 0,008 | 0,016 | -0,832 | 0,770 | 1,603 | 0,34 | 0,013 |
| YLDs | Both | Luxembourg | -0,022 | -0,028 | -0,006 | -2,182 | -2,772 | -0,590 | 0,95 | 0,124 |
| YLDs | Both | Malta | -0,019 | -0,029 | -0,010 | -1,927 | -2,879 | -0,953 | 0,95 | 0,004 |
| YLDs | Both | Monaco | -0,021 | -0,008 | 0,014 | -2,125 | -0,769 | 1,356 | 0,93 | <0,0001 |
| YLDs | Both | Montenegro | -0,012 | -0,003 | 0,008 | -1,149 | -0,309 | 0,840 | 0,92 | <0,0001 |
| YLDs | Both | Netherlands | -0,014 | -0,008 | 0,006 | -1,405 | -0,818 | 0,587 | 0,96 | <0,0001 |
| YLDs | Both | North Macedonia | -0,009 | -0,001 | 0,008 | -0,851 | -0,085 | 0,766 | 0,89 | <0,0001 |
| YLDs | Both | Norway | -0,012 | -0,006 | 0,006 | -1,221 | -0,618 | 0,603 | 0,94 | 0,001 |
| YLDs | Both | Poland | -0,002 | -0,007 | -0,005 | -0,183 | -0,706 | -0,523 | 0,37 | <0,0001 |
| YLDs | Both | Portugal | -0,027 | -0,018 | 0,009 | -2,624 | -1,766 | 0,858 | 0,96 | 0,013 |
| YLDs | Both | Republic of Moldova | -0,004 | 0,004 | 0,008 | -0,365 | 0,413 | 0,778 | 0,71 | <0,0001 |
| YLDs | Both | Romania | -0,015 | -0,005 | 0,010 | -1,449 | -0,453 | 0,996 | 0,93 | <0,0001 |
| YLDs | Both | Russian Federation | -0,007 | 0,001 | 0,008 | -0,680 | 0,131 | 0,812 | 0,83 | <0,0001 |
| YLDs | Both | San Marino | -0,015 | -0,016 | -0,001 | -1,486 | -1,584 | -0,098 | 0,97 | 0,605 |
| YLDs | Both | Serbia | -0,015 | -0,010 | 0,005 | -1,518 | -1,041 | 0,477 | 0,96 | 0,013 |
| YLDs | Both | Slovakia | -0,019 | 0,009 | 0,027 | -1,861 | 0,873 | 2,734 | 0,80 | <0,0001 |
| YLDs | Both | Slovenia | -0,015 | -0,015 | 0,000 | -1,517 | -1,475 | 0,043 | 0,98 | 0,764 |
| YLDs | Both | Spain | -0,010 | -0,010 | -0,001 | -0,951 | -1,040 | -0,089 | 0,98 | 0,264 |
| YLDs | Both | Sweden | -0,006 | -0,017 | -0,011 | -0,591 | -1,689 | -1,097 | 0,76 | <0,0001 |
| YLDs | Both | Switzerland | -0,013 | -0,013 | -0,001 | -1,264 | -1,336 | -0,072 | 0,98 | 0,563 |
| YLDs | Both | Ukraine | -0,012 | -0,003 | 0,010 | -1,229 | -0,285 | 0,944 | 0,91 | <0,0001 |
| YLDs | Both | United Kingdom | -0,016 | -0,012 | 0,004 | -1,576 | -1,180 | 0,395 | 0,97 | 0,064 |
| YLDs | Female | Albania | -0,006 | -0,002 | 0,005 | -0,642 | -0,179 | 0,463 | 0,93 | <0,0001 |
| YLDs | Female | Andorra | -0,015 | -0,013 | 0,002 | -1,496 | -1,319 | 0,177 | 0,97 | 0,360 |
| YLDs | Female | Austria | -0,001 | 0,014 | 0,015 | -0,099 | 1,402 | 1,502 | 0,05 | <0,0001 |
| YLDs | Female | Belarus | -0,011 | -0,004 | 0,007 | -1,078 | -0,354 | 0,724 | 0,93 | <0,0001 |
| YLDs | Female | Belgium | -0,012 | -0,011 | 0,001 | -1,216 | -1,137 | 0,079 | 0,98 | 0,576 |
| YLDs | Female | Bosnia and Herzegovina | -0,009 | -0,001 | 0,009 | -0,923 | -0,053 | 0,870 | 0,90 | <0,0001 |
| YLDs | Female | Bulgaria | -0,006 | -0,002 | 0,004 | -0,637 | -0,215 | 0,422 | 0,92 | 0,002 |
| YLDs | Female | Croatia | -0,014 | -0,006 | 0,008 | -1,397 | -0,602 | 0,794 | 0,95 | <0,0001 |
| YLDs | Female | Cyprus | -0,010 | -0,023 | -0,013 | -0,985 | -2,288 | -1,303 | 0,82 | <0,0001 |
| YLDs | Female | Czechia | -0,019 | 0,000 | 0,019 | -1,868 | 0,045 | 1,913 | 0,90 | <0,0001 |
| YLDs | Female | Denmark | -0,015 | 0,000 | 0,015 | -1,484 | -0,014 | 1,470 | 0,91 | <0,0001 |
| YLDs | Female | Estonia | -0,017 | -0,004 | 0,013 | -1,730 | -0,432 | 1,298 | 0,93 | <0,0001 |
| YLDs | Female | Finland | -0,010 | 0,008 | 0,019 | -1,042 | 0,821 | 1,863 | 0,77 | <0,0001 |
| YLDs | Female | France | 0,000 | 0,006 | 0,006 | -0,013 | 0,609 | 0,622 | 0,01 | <0,0001 |
| YLDs | Female | Germany | -0,006 | -0,006 | 0,000 | -0,594 | -0,574 | 0,020 | 0,98 | 0,664 |
| YLDs | Female | Greece | -0,017 | -0,014 | 0,003 | -1,668 | -1,414 | 0,254 | 0,97 | 0,140 |
| YLDs | Female | Hungary | -0,018 | -0,002 | 0,016 | -1,770 | -0,225 | 1,545 | 0,90 | <0,0001 |
| YLDs | Female | Iceland | -0,012 | -0,011 | 0,001 | -1,225 | -1,099 | 0,127 | 0,98 | 0,346 |
| YLDs | Female | Ireland | -0,019 | -0,023 | -0,005 | -1,846 | -2,287 | -0,441 | 0,97 | 0,096 |
| YLDs | Female | Israel | -0,014 | 0,001 | 0,015 | -1,346 | 0,135 | 1,481 | 0,90 | <0,0001 |
| YLDs | Female | Italy | -0,010 | -0,012 | -0,002 | -1,020 | -1,233 | -0,213 | 0,97 | 0,064 |
| YLDs | Female | Latvia | -0,014 | -0,008 | 0,006 | -1,388 | -0,758 | 0,630 | 0,96 | <0,0001 |
| YLDs | Female | Lithuania | -0,012 | 0,007 | 0,019 | -1,188 | 0,740 | 1,928 | 0,51 | 0,003 |
| YLDs | Female | Luxembourg | -0,022 | -0,026 | -0,004 | -2,178 | -2,540 | -0,362 | 0,95 | 0,340 |
| YLDs | Female | Malta | -0,017 | -0,027 | -0,010 | -1,732 | -2,668 | -0,935 | 0,95 | 0,001 |
| YLDs | Female | Monaco | -0,015 | -0,004 | 0,011 | -1,470 | -0,356 | 1,114 | 0,92 | <0,0001 |
| YLDs | Female | Montenegro | -0,011 | -0,003 | 0,009 | -1,126 | -0,254 | 0,872 | 0,91 | <0,0001 |
| YLDs | Female | Netherlands | -0,014 | -0,002 | 0,012 | -1,408 | -0,215 | 1,193 | 0,92 | <0,0001 |
| YLDs | Female | North Macedonia | -0,011 | 0,001 | 0,012 | -1,108 | 0,078 | 1,186 | 0,87 | <0,0001 |
| YLDs | Female | Norway | -0,013 | -0,007 | 0,005 | -1,276 | -0,733 | 0,543 | 0,94 | 0,005 |
| YLDs | Female | Poland | -0,005 | -0,014 | -0,009 | -0,481 | -1,399 | -0,918 | 0,62 | <0,0001 |
| YLDs | Female | Portugal | -0,030 | -0,010 | 0,020 | -2,965 | -0,990 | 1,975 | 0,93 | <0,0001 |
| YLDs | Female | Republic of Moldova | -0,006 | 0,001 | 0,008 | -0,641 | 0,134 | 0,775 | 0,87 | <0,0001 |
| YLDs | Female | Romania | -0,015 | -0,005 | 0,010 | -1,508 | -0,546 | 0,963 | 0,93 | <0,0001 |
| YLDs | Female | Russian Federation | -0,010 | -0,001 | 0,009 | -0,964 | -0,084 | 0,880 | 0,88 | <0,0001 |
| YLDs | Female | San Marino | -0,011 | -0,011 | 0,000 | -1,123 | -1,090 | 0,033 | 0,97 | 0,826 |
| YLDs | Female | Serbia | -0,015 | -0,008 | 0,007 | -1,460 | -0,777 | 0,683 | 0,95 | 0,001 |
| YLDs | Female | Slovakia | -0,017 | 0,011 | 0,028 | -1,702 | 1,060 | 2,762 | 0,78 | <0,0001 |
| YLDs | Female | Slovenia | -0,015 | -0,015 | -0,001 | -1,449 | -1,499 | -0,050 | 0,98 | 0,726 |
| YLDs | Female | Spain | -0,016 | -0,023 | -0,007 | -1,597 | -2,253 | -0,656 | 0,97 | <0,0001 |
| YLDs | Female | Sweden | -0,005 | -0,013 | -0,008 | -0,508 | -1,329 | -0,821 | 0,80 | <0,0001 |
| YLDs | Female | Switzerland | -0,012 | -0,014 | -0,002 | -1,226 | -1,412 | -0,186 | 0,98 | 0,183 |
| YLDs | Female | Ukraine | -0,012 | -0,002 | 0,010 | -1,192 | -0,217 | 0,975 | 0,90 | <0,0001 |
| YLDs | Female | United Kingdom | -0,014 | -0,011 | 0,003 | -1,427 | -1,122 | 0,305 | 0,97 | 0,096 |
| YLDs | Male | Albania | -0,014 | -0,006 | 0,008 | -1,381 | -0,563 | 0,818 | 0,94 | <0,0001 |
| YLDs | Male | Andorra | -0,016 | -0,017 | -0,001 | -1,608 | -1,726 | -0,118 | 0,97 | 0,565 |
| YLDs | Male | Austria | -0,005 | 0,020 | 0,025 | -0,540 | 2,001 | 2,541 | 0,33 | <0,0001 |
| YLDs | Male | Belarus | -0,012 | 0,000 | 0,012 | -1,149 | 0,033 | 1,182 | 0,86 | <0,0001 |
| YLDs | Male | Belgium | -0,013 | -0,013 | 0,000 | -1,259 | -1,303 | -0,044 | 0,99 | 0,652 |
| YLDs | Male | Bosnia and Herzegovina | -0,011 | -0,005 | 0,006 | -1,111 | -0,501 | 0,610 | 0,95 | <0,0001 |
| YLDs | Male | Bulgaria | -0,007 | -0,007 | 0,000 | -0,699 | -0,727 | -0,028 | 0,98 | 0,737 |
| YLDs | Male | Croatia | -0,011 | 0,003 | 0,014 | -1,110 | 0,252 | 1,362 | 0,87 | <0,0001 |
| YLDs | Male | Cyprus | -0,016 | -0,029 | -0,013 | -1,560 | -2,826 | -1,266 | 0,91 | <0,0001 |
| YLDs | Male | Czechia | -0,021 | -0,009 | 0,012 | -2,104 | -0,905 | 1,199 | 0,96 | <0,0001 |
| YLDs | Male | Denmark | -0,018 | -0,003 | 0,015 | -1,765 | -0,330 | 1,435 | 0,93 | <0,0001 |
| YLDs | Male | Estonia | -0,017 | -0,008 | 0,009 | -1,708 | -0,846 | 0,863 | 0,95 | <0,0001 |
| YLDs | Male | Finland | -0,017 | 0,004 | 0,020 | -1,647 | 0,369 | 2,016 | 0,86 | <0,0001 |
| YLDs | Male | France | 0,001 | 0,005 | 0,005 | 0,065 | 0,523 | 0,458 | 0,16 | <0,0001 |
| YLDs | Male | Germany | -0,002 | 0,002 | 0,004 | -0,165 | 0,250 | 0,416 | 0,64 | <0,0001 |
| YLDs | Male | Greece | -0,016 | -0,015 | 0,001 | -1,610 | -1,484 | 0,126 | 0,98 | 0,418 |
| YLDs | Male | Hungary | -0,017 | -0,007 | 0,010 | -1,704 | -0,718 | 0,986 | 0,94 | <0,0001 |
| YLDs | Male | Iceland | -0,017 | -0,012 | 0,005 | -1,657 | -1,153 | 0,504 | 0,97 | 0,003 |
| YLDs | Male | Ireland | -0,022 | -0,035 | -0,013 | -2,205 | -3,475 | -1,270 | 0,95 | <0,0001 |
| YLDs | Male | Israel | -0,020 | 0,006 | 0,026 | -1,961 | 0,651 | 2,612 | 0,86 | <0,0001 |
| YLDs | Male | Italy | -0,009 | -0,012 | -0,003 | -0,847 | -1,186 | -0,339 | 0,97 | 0,001 |
| YLDs | Male | Latvia | -0,011 | 0,002 | 0,013 | -1,105 | 0,164 | 1,269 | 0,87 | <0,0001 |
| YLDs | Male | Lithuania | -0,003 | 0,008 | 0,011 | -0,316 | 0,820 | 1,137 | 0,07 | 0,088 |
| YLDs | Male | Luxembourg | -0,023 | -0,030 | -0,007 | -2,292 | -2,995 | -0,703 | 0,95 | 0,072 |
| YLDs | Male | Malta | -0,022 | -0,031 | -0,009 | -2,174 | -3,016 | -0,842 | 0,95 | 0,025 |
| YLDs | Male | Monaco | -0,028 | -0,012 | 0,016 | -2,783 | -1,189 | 1,594 | 0,94 | <0,0001 |
| YLDs | Male | Montenegro | -0,012 | -0,002 | 0,010 | -1,236 | -0,239 | 0,996 | 0,91 | <0,0001 |
| YLDs | Male | Netherlands | -0,015 | -0,016 | -0,001 | -1,529 | -1,622 | -0,093 | 0,98 | 0,508 |
| YLDs | Male | North Macedonia | -0,006 | -0,002 | 0,004 | -0,588 | -0,162 | 0,426 | 0,91 | <0,0001 |
| YLDs | Male | Norway | -0,013 | -0,005 | 0,008 | -1,321 | -0,489 | 0,833 | 0,93 | <0,0001 |
| YLDs | Male | Poland | 0,001 | 0,002 | 0,001 | 0,090 | 0,161 | 0,071 | 0,34 | 0,410 |
| YLDs | Male | Portugal | -0,022 | -0,028 | -0,006 | -2,204 | -2,745 | -0,541 | 0,97 | 0,072 |
| YLDs | Male | Republic of Moldova | -0,001 | 0,007 | 0,008 | -0,059 | 0,705 | 0,764 | 0,06 | <0,0001 |
| YLDs | Male | Romania | -0,013 | -0,003 | 0,010 | -1,331 | -0,326 | 1,005 | 0,91 | <0,0001 |
| YLDs | Male | Russian Federation | -0,003 | 0,003 | 0,006 | -0,313 | 0,269 | 0,582 | 0,66 | <0,0001 |
| YLDs | Male | San Marino | -0,019 | -0,020 | -0,002 | -1,861 | -2,026 | -0,165 | 0,97 | 0,475 |
| YLDs | Male | Serbia | -0,016 | -0,012 | 0,004 | -1,589 | -1,218 | 0,371 | 0,97 | 0,069 |
| YLDs | Male | Slovakia | -0,021 | 0,008 | 0,029 | -2,050 | 0,851 | 2,900 | 0,80 | <0,0001 |
| YLDs | Male | Slovenia | -0,018 | -0,015 | 0,002 | -1,760 | -1,535 | 0,225 | 0,98 | 0,151 |
| YLDs | Male | Spain | -0,005 | 0,001 | 0,006 | -0,458 | 0,104 | 0,563 | 0,86 | <0,0001 |
| YLDs | Male | Sweden | -0,008 | -0,021 | -0,013 | -0,790 | -2,072 | -1,281 | 0,80 | <0,0001 |
| YLDs | Male | Switzerland | -0,014 | -0,013 | 0,001 | -1,406 | -1,331 | 0,076 | 0,98 | 0,515 |
| YLDs | Male | Ukraine | -0,015 | -0,007 | 0,008 | -1,457 | -0,679 | 0,778 | 0,94 | 0,001 |
| YLDs | Male | United Kingdom | -0,018 | -0,014 | 0,004 | -1,817 | -1,385 | 0,432 | 0,96 | 0,083 |
| yll | Both | Albania | -0,021 | -0,012 | 0,009 | -2,050 | -1,184 | 0,865 | 0,95 | 0,013 |
| yll | Both | Andorra | -0,034 | -0,052 | -0,018 | -3,363 | -5,078 | -1,715 | 0,98 | <0,0001 |
| yll | Both | Austria | -0,056 | -0,093 | -0,037 | -5,486 | -8,906 | -3,421 | 0,95 | <0,0001 |
| yll | Both | Belarus | -0,026 | 0,006 | 0,033 | -2,614 | 0,634 | 3,248 | 0,85 | <0,0001 |
| yll | Both | Belgium | -0,043 | -0,057 | -0,014 | -4,174 | -5,519 | -1,345 | 0,99 | <0,0001 |
| yll | Both | Bosnia and Herzegovina | -0,016 | -0,003 | 0,013 | -1,582 | -0,300 | 1,282 | 0,93 | <0,0001 |
| yll | Both | Bulgaria | -0,011 | -0,008 | 0,003 | -1,115 | -0,799 | 0,317 | 0,95 | 0,092 |
| yll | Both | Croatia | -0,038 | -0,012 | 0,026 | -3,736 | -1,218 | 2,519 | 0,96 | <0,0001 |
| yll | Both | Cyprus | -0,048 | -0,055 | -0,007 | -4,697 | -5,350 | -0,653 | 0,99 | 0,085 |
| yll | Both | Czechia | -0,061 | -0,046 | 0,015 | -5,894 | -4,475 | 1,419 | 0,98 | 0,021 |
| yll | Both | Denmark | -0,038 | -0,027 | 0,011 | -3,757 | -2,688 | 1,069 | 0,97 | 0,021 |
| yll | Both | Estonia | -0,069 | -0,061 | 0,008 | -6,711 | -5,925 | 0,786 | 0,93 | 0,570 |
| yll | Both | Finland | -0,040 | -0,060 | -0,020 | -3,949 | -5,817 | -1,868 | 0,98 | <0,0001 |
| yll | Both | France | -0,036 | -0,049 | -0,013 | -3,501 | -4,739 | -1,238 | 0,98 | <0,0001 |
| yll | Both | Germany | -0,043 | -0,078 | -0,035 | -4,207 | -7,547 | -3,340 | 0,95 | <0,0001 |
| yll | Both | Greece | -0,050 | -0,048 | 0,002 | -4,865 | -4,645 | 0,220 | 0,97 | 0,731 |
| yll | Both | Hungary | -0,039 | -0,045 | -0,006 | -3,861 | -4,391 | -0,530 | 0,97 | 0,242 |
| yll | Both | Iceland | -0,040 | -0,037 | 0,003 | -3,950 | -3,629 | 0,321 | 0,98 | 0,370 |
| yll | Both | Ireland | -0,055 | -0,054 | 0,001 | -5,390 | -5,302 | 0,088 | 0,99 | 0,839 |
| yll | Both | Israel | -0,046 | -0,055 | -0,009 | -4,506 | -5,351 | -0,845 | 0,98 | 0,066 |
| yll | Both | Italy | -0,045 | -0,071 | -0,026 | -4,382 | -6,843 | -2,461 | 0,97 | <0,0001 |
| yll | Both | Latvia | -0,023 | -0,019 | 0,005 | -2,298 | -1,844 | 0,454 | 0,92 | 0,394 |
| yll | Both | Lithuania | -0,019 | 0,008 | 0,027 | -1,844 | 0,812 | 2,656 | 0,83 | <0,0001 |
| yll | Both | Luxembourg | -0,060 | -0,073 | -0,013 | -5,785 | -7,021 | -1,236 | 0,99 | 0,002 |
| yll | Both | Malta | -0,050 | -0,037 | 0,013 | -4,900 | -3,658 | 1,242 | 0,99 | 0,001 |
| yll | Both | Monaco | -0,051 | -0,005 | 0,046 | -4,972 | -0,538 | 4,434 | 0,95 | <0,0001 |
| yll | Both | Montenegro | -0,020 | 0,011 | 0,031 | -1,972 | 1,111 | 3,083 | 0,83 | <0,0001 |
| yll | Both | Netherlands | -0,036 | -0,041 | -0,005 | -3,550 | -4,065 | -0,515 | 0,96 | 0,353 |
| yll | Both | North Macedonia | -0,019 | 0,008 | 0,026 | -1,835 | 0,780 | 2,615 | 0,87 | <0,0001 |
| yll | Both | Norway | -0,051 | -0,046 | 0,005 | -4,989 | -4,487 | 0,502 | 0,99 | 0,208 |
| yll | Both | Poland | -0,040 | -0,040 | 0,000 | -3,879 | -3,921 | -0,042 | 0,99 | 0,857 |
| yll | Both | Portugal | -0,062 | -0,076 | -0,013 | -6,057 | -7,303 | -1,246 | 0,98 | 0,020 |
| yll | Both | Republic of Moldova | -0,013 | 0,020 | 0,033 | -1,283 | 1,979 | 3,262 | 0,42 | <0,0001 |
| yll | Both | Romania | -0,025 | 0,000 | 0,025 | -2,467 | -0,009 | 2,458 | 0,91 | <0,0001 |
| yll | Both | Russian Federation | -0,031 | 0,010 | 0,041 | -3,054 | 0,978 | 4,032 | 0,83 | <0,0001 |
| yll | Both | San Marino | -0,048 | -0,055 | -0,007 | -4,676 | -5,386 | -0,710 | 0,99 | 0,086 |
| yll | Both | Serbia | -0,028 | -0,011 | 0,017 | -2,772 | -1,138 | 1,635 | 0,96 | <0,0001 |
| yll | Both | Slovakia | -0,033 | -0,020 | 0,013 | -3,249 | -1,962 | 1,287 | 0,98 | <0,0001 |
| yll | Both | Slovenia | -0,047 | -0,069 | -0,022 | -4,557 | -6,625 | -2,068 | 0,98 | <0,0001 |
| yll | Both | Spain | -0,053 | -0,082 | -0,029 | -5,157 | -7,893 | -2,737 | 0,97 | <0,0001 |
| yll | Both | Sweden | -0,042 | -0,023 | 0,019 | -4,108 | -2,288 | 1,820 | 0,98 | <0,0001 |
| yll | Both | Switzerland | -0,042 | -0,061 | -0,019 | -4,157 | -5,941 | -1,784 | 0,98 | <0,0001 |
| yll | Both | Ukraine | -0,033 | -0,020 | 0,012 | -3,217 | -2,007 | 1,210 | 0,97 | 0,002 |
| yll | Both | United Kingdom | -0,051 | -0,050 | 0,000 | -4,942 | -4,918 | 0,024 | 0,98 | 0,970 |
| yll | Female | Albania | -0,021 | -0,011 | 0,010 | -2,056 | -1,107 | 0,949 | 0,94 | 0,010 |
| yll | Female | Andorra | -0,035 | -0,057 | -0,022 | -3,398 | -5,515 | -2,117 | 0,97 | <0,0001 |
| yll | Female | Austria | -0,058 | -0,094 | -0,036 | -5,635 | -8,940 | -3,305 | 0,96 | <0,0001 |
| yll | Female | Belarus | -0,027 | 0,002 | 0,030 | -2,711 | 0,238 | 2,949 | 0,88 | <0,0001 |
| yll | Female | Belgium | -0,043 | -0,056 | -0,013 | -4,193 | -5,422 | -1,229 | 0,99 | <0,0001 |
| yll | Female | Bosnia and Herzegovina | -0,018 | -0,005 | 0,013 | -1,748 | -0,456 | 1,292 | 0,94 | <0,0001 |
| yll | Female | Bulgaria | -0,014 | -0,007 | 0,006 | -1,343 | -0,729 | 0,614 | 0,96 | 0,001 |
| yll | Female | Croatia | -0,039 | -0,014 | 0,026 | -3,871 | -1,369 | 2,502 | 0,96 | <0,0001 |
| yll | Female | Cyprus | -0,049 | -0,058 | -0,009 | -4,795 | -5,632 | -0,837 | 0,99 | 0,030 |
| yll | Female | Czechia | -0,062 | -0,042 | 0,021 | -6,044 | -4,072 | 1,972 | 0,97 | 0,003 |
| yll | Female | Denmark | -0,039 | -0,028 | 0,011 | -3,837 | -2,807 | 1,031 | 0,98 | 0,018 |
| yll | Female | Estonia | -0,079 | -0,065 | 0,014 | -7,592 | -6,301 | 1,291 | 0,94 | 0,373 |
| yll | Female | Finland | -0,040 | -0,061 | -0,021 | -3,934 | -5,954 | -2,020 | 0,97 | <0,0001 |
| yll | Female | France | -0,036 | -0,048 | -0,012 | -3,522 | -4,655 | -1,133 | 0,98 | <0,0001 |
| yll | Female | Germany | -0,044 | -0,076 | -0,032 | -4,351 | -7,336 | -2,985 | 0,96 | <0,0001 |
| yll | Female | Greece | -0,053 | -0,047 | 0,006 | -5,129 | -4,549 | 0,580 | 0,97 | 0,393 |
| yll | Female | Hungary | -0,042 | -0,049 | -0,007 | -4,077 | -4,740 | -0,663 | 0,97 | 0,146 |
| yll | Female | Iceland | -0,037 | -0,030 | 0,007 | -3,630 | -2,987 | 0,642 | 0,97 | 0,142 |
| yll | Female | Ireland | -0,055 | -0,052 | 0,003 | -5,327 | -5,068 | 0,259 | 0,99 | 0,543 |
| yll | Female | Israel | -0,046 | -0,051 | -0,004 | -4,537 | -4,931 | -0,393 | 0,99 | 0,314 |
| yll | Female | Italy | -0,044 | -0,069 | -0,025 | -4,299 | -6,628 | -2,329 | 0,97 | <0,0001 |
| yll | Female | Latvia | -0,026 | -0,021 | 0,006 | -2,594 | -2,038 | 0,555 | 0,93 | 0,325 |
| yll | Female | Lithuania | -0,024 | 0,007 | 0,031 | -2,388 | 0,731 | 3,119 | 0,86 | <0,0001 |
| yll | Female | Luxembourg | -0,061 | -0,071 | -0,010 | -5,903 | -6,859 | -0,956 | 0,99 | 0,057 |
| yll | Female | Malta | -0,052 | -0,036 | 0,017 | -5,105 | -3,498 | 1,607 | 0,98 | 0,002 |
| yll | Female | Monaco | -0,049 | -0,014 | 0,035 | -4,773 | -1,359 | 3,414 | 0,96 | <0,0001 |
| yll | Female | Montenegro | -0,020 | 0,011 | 0,030 | -1,966 | 1,060 | 3,026 | 0,83 | <0,0001 |
| yll | Female | Netherlands | -0,036 | -0,038 | -0,002 | -3,514 | -3,745 | -0,231 | 0,96 | 0,660 |
| yll | Female | North Macedonia | -0,019 | 0,010 | 0,029 | -1,851 | 1,025 | 2,876 | 0,85 | <0,0001 |
| yll | Female | Norway | -0,051 | -0,047 | 0,003 | -4,928 | -4,604 | 0,324 | 0,99 | 0,495 |
| yll | Female | Poland | -0,044 | -0,043 | 0,001 | -4,297 | -4,192 | 0,105 | 0,99 | 0,670 |
| yll | Female | Portugal | -0,064 | -0,072 | -0,009 | -6,158 | -6,982 | -0,824 | 0,99 | 0,114 |
| yll | Female | Republic of Moldova | -0,019 | 0,012 | 0,030 | -1,838 | 1,200 | 3,037 | 0,57 | <0,0001 |
| yll | Female | Romania | -0,027 | -0,002 | 0,025 | -2,701 | -0,226 | 2,475 | 0,93 | <0,0001 |
| yll | Female | Russian Federation | -0,034 | 0,006 | 0,041 | -3,377 | 0,640 | 4,018 | 0,86 | <0,0001 |
| yll | Female | San Marino | -0,044 | -0,053 | -0,009 | -4,312 | -5,208 | -0,896 | 0,98 | 0,024 |
| yll | Female | Serbia | -0,029 | -0,012 | 0,017 | -2,835 | -1,155 | 1,680 | 0,96 | <0,0001 |
| yll | Female | Slovakia | -0,037 | -0,025 | 0,012 | -3,649 | -2,458 | 1,191 | 0,99 | <0,0001 |
| yll | Female | Slovenia | -0,049 | -0,066 | -0,017 | -4,771 | -6,342 | -1,571 | 0,99 | <0,0001 |
| yll | Female | Spain | -0,056 | -0,081 | -0,025 | -5,437 | -7,809 | -2,373 | 0,98 | <0,0001 |
| yll | Female | Sweden | -0,041 | -0,018 | 0,023 | -4,059 | -1,816 | 2,243 | 0,97 | <0,0001 |
| yll | Female | Switzerland | -0,042 | -0,059 | -0,017 | -4,123 | -5,734 | -1,611 | 0,99 | <0,0001 |
| yll | Female | Ukraine | -0,036 | -0,022 | 0,014 | -3,492 | -2,140 | 1,353 | 0,97 | <0,0001 |
| yll | Female | United Kingdom | -0,050 | -0,047 | 0,003 | -4,907 | -4,589 | 0,318 | 0,98 | 0,609 |
| yll | Male | Albania | -0,020 | -0,012 | 0,008 | -2,025 | -1,231 | 0,794 | 0,95 | 0,025 |
| yll | Male | Andorra | -0,034 | -0,048 | -0,014 | -3,337 | -4,663 | -1,326 | 0,98 | <0,0001 |
| yll | Male | Austria | -0,057 | -0,095 | -0,038 | -5,516 | -9,032 | -3,516 | 0,95 | <0,0001 |
| yll | Male | Belarus | -0,025 | 0,008 | 0,033 | -2,512 | 0,764 | 3,276 | 0,83 | <0,0001 |
| yll | Male | Belgium | -0,044 | -0,059 | -0,015 | -4,287 | -5,729 | -1,442 | 0,99 | <0,0001 |
| yll | Male | Bosnia and Herzegovina | -0,014 | -0,001 | 0,013 | -1,427 | -0,137 | 1,291 | 0,89 | <0,0001 |
| yll | Male | Bulgaria | -0,009 | -0,008 | 0,001 | -0,917 | -0,803 | 0,114 | 0,92 | 0,595 |
| yll | Male | Croatia | -0,038 | -0,011 | 0,027 | -3,748 | -1,113 | 2,635 | 0,96 | <0,0001 |
| yll | Male | Cyprus | -0,047 | -0,052 | -0,005 | -4,596 | -5,059 | -0,463 | 0,99 | 0,244 |
| yll | Male | Czechia | -0,061 | -0,052 | 0,009 | -5,895 | -5,051 | 0,844 | 0,98 | 0,144 |
| yll | Male | Denmark | -0,039 | -0,026 | 0,013 | -3,793 | -2,572 | 1,221 | 0,97 | 0,018 |
| yll | Male | Estonia | -0,061 | -0,055 | 0,005 | -5,897 | -5,391 | 0,506 | 0,92 | 0,688 |
| yll | Male | Finland | -0,042 | -0,061 | -0,019 | -4,096 | -5,916 | -1,820 | 0,98 | <0,0001 |
| yll | Male | France | -0,037 | -0,052 | -0,014 | -3,660 | -5,022 | -1,361 | 0,98 | <0,0001 |
| yll | Male | Germany | -0,044 | -0,085 | -0,041 | -4,330 | -8,172 | -3,842 | 0,93 | <0,0001 |
| yll | Male | Greece | -0,047 | -0,050 | -0,003 | -4,572 | -4,859 | -0,287 | 0,97 | 0,635 |
| yll | Male | Hungary | -0,038 | -0,040 | -0,002 | -3,702 | -3,883 | -0,181 | 0,97 | 0,690 |
| yll | Male | Iceland | -0,045 | -0,043 | 0,001 | -4,362 | -4,226 | 0,136 | 0,98 | 0,700 |
| yll | Male | Ireland | -0,057 | -0,058 | -0,001 | -5,530 | -5,595 | -0,065 | 0,99 | 0,890 |
| yll | Male | Israel | -0,047 | -0,061 | -0,015 | -4,556 | -5,942 | -1,386 | 0,97 | 0,014 |
| yll | Male | Italy | -0,047 | -0,073 | -0,026 | -4,596 | -7,025 | -2,429 | 0,97 | <0,0001 |
| yll | Male | Latvia | -0,021 | -0,018 | 0,003 | -2,078 | -1,748 | 0,330 | 0,91 | 0,516 |
| yll | Male | Lithuania | -0,013 | 0,010 | 0,023 | -1,317 | 0,990 | 2,307 | 0,76 | <0,0001 |
| yll | Male | Luxembourg | -0,059 | -0,074 | -0,015 | -5,754 | -7,150 | -1,396 | 0,99 | <0,0001 |
| yll | Male | Malta | -0,048 | -0,038 | 0,010 | -4,725 | -3,755 | 0,970 | 0,99 | 0,001 |
| yll | Male | Monaco | -0,055 | 0,004 | 0,059 | -5,324 | 0,382 | 5,706 | 0,92 | <0,0001 |
| yll | Male | Montenegro | -0,020 | 0,010 | 0,030 | -2,000 | 1,016 | 3,016 | 0,84 | <0,0001 |
| yll | Male | Netherlands | -0,038 | -0,048 | -0,010 | -3,728 | -4,693 | -0,966 | 0,95 | 0,113 |
| yll | Male | North Macedonia | -0,019 | 0,005 | 0,023 | -1,849 | 0,470 | 2,319 | 0,89 | <0,0001 |
| yll | Male | Norway | -0,053 | -0,045 | 0,008 | -5,155 | -4,419 | 0,736 | 0,99 | 0,038 |
| yll | Male | Poland | -0,036 | -0,037 | -0,001 | -3,573 | -3,644 | -0,071 | 0,99 | 0,759 |
| yll | Male | Portugal | -0,062 | -0,080 | -0,018 | -6,012 | -7,708 | -1,697 | 0,98 | 0,002 |
| yll | Male | Republic of Moldova | -0,008 | 0,029 | 0,037 | -0,765 | 2,985 | 3,750 | 0,22 | <0,0001 |
| yll | Male | Romania | -0,022 | 0,002 | 0,025 | -2,217 | 0,230 | 2,447 | 0,89 | <0,0001 |
| yll | Male | Russian Federation | -0,029 | 0,011 | 0,040 | -2,856 | 1,131 | 3,987 | 0,81 | <0,0001 |
| yll | Male | San Marino | -0,053 | -0,059 | -0,006 | -5,152 | -5,695 | -0,543 | 0,99 | 0,230 |
| yll | Male | Serbia | -0,028 | -0,011 | 0,016 | -2,747 | -1,129 | 1,618 | 0,96 | <0,0001 |
| yll | Male | Slovakia | -0,030 | -0,013 | 0,016 | -2,947 | -1,340 | 1,607 | 0,97 | <0,0001 |
| yll | Male | Slovenia | -0,047 | -0,072 | -0,026 | -4,566 | -6,985 | -2,419 | 0,98 | <0,0001 |
| yll | Male | Spain | -0,051 | -0,084 | -0,033 | -4,967 | -8,080 | -3,114 | 0,96 | <0,0001 |
| yll | Male | Sweden | -0,043 | -0,028 | 0,015 | -4,224 | -2,743 | 1,481 | 0,98 | <0,0001 |
| yll | Male | Switzerland | -0,044 | -0,063 | -0,019 | -4,317 | -6,112 | -1,796 | 0,98 | <0,0001 |
| yll | Male | Ukraine | -0,030 | -0,022 | 0,008 | -2,979 | -2,172 | 0,807 | 0,97 | 0,050 |
| yll | Male | United Kingdom | -0,052 | -0,055 | -0,003 | -5,043 | -5,312 | -0,269 | 0,98 | 0,670 |
