# Supplementary material for: Trends in mortality and disability from ischaemic stroke in Europe, 1990-2023
Source: Eur Stroke J. 2026 Jul 21;11(7):aakag082. doi: 10.1093/esj/aakag082 (PMC13387428; doi:10.1093/esj/aakag082)
Supplement: Supplementary_material_aakag082 [file supplementary_material_aakag082.zip › Supplementary Table 4.docx]

**Supplementary Table 4.** Country‑specific Best Linear Unbiased Predictions of the Estimated Annual Percentage Change (BLUP‑EAPC) from mixed‑effects models (DALY, YLD, YLL)

| **Country** | **BLUP‑EAPC DALY (%)** | **BLUP‑EAPC YLD (%)** | **BLUP‑EAPC YLL (%)** |
| --- | --- | --- | --- |
| Albania | −45.85 | −49.86 | −11.12 |
| Andorra | −66.47 | −71.62 | −21.41 |
| Austria | −36.56 | −37.13 | −18.05 |
| Belarus | 86.63 | 92.93 | 33.25 |
| Belgium | −47.35 | −48.82 | −30.31 |
| Bosnia and Herzegovina | 64.53 | 66.66 | 43.95 |
| Bulgaria | 85.62 | 87.87 | 63.32 |
| Croatia | 43.34 | 48.19 | 9.02 |
| Cyprus | −11.57 | −7.04 | −43.04 |
| Czechia | 93.38 | 102.24 | 41.62 |
| Denmark | −40.27 | −43.26 | −11.61 |
| Estonia | 100.20 | 114.05 | 20.01 |
| Finland | −33.82 | −37.84 | 2.21 |
| France | −64.15 | −65.65 | −46.26 |
| Germany | −36.41 | −39.25 | −3.98 |
| Greece | 7.76 | 10.61 | −7.45 |
| Hungary | 61.78 | 62.74 | 55.15 |
| Iceland | −53.43 | −56.21 | −25.60 |
| Ireland | −35.65 | −35.05 | −31.35 |
| Israel | −54.79 | −60.56 | −2.13 |
| Italy | −42.02 | −41.24 | −43.82 |
| Latvia | 85.91 | 91.26 | 37.51 |
| Lithuania | 18.44 | 17.84 | 21.75 |
| Luxembourg | −16.66 | −14.77 | −22.77 |
| Malta | −32.21 | −31.39 | −31.34 |
| Monaco | 9.80 | 11.16 | 11.88 |
| Montenegro | 65.19 | 70.10 | 21.13 |
| Netherlands | −46.16 | −49.61 | −14.50 |
| North Macedonia | 149.21 | 158.90 | 63.42 |
| Norway | −35.52 | −36.90 | −10.86 |
| Poland | 40.01 | 48.46 | −20.40 |
| Portugal | 56.41 | 59.96 | 37.61 |
| Republic of Moldova | 3.29 | 3.97 | −3.23 |
| Romania | 93.94 | 100.21 | 38.38 |
| Russian Federation | 181.17 | 200.35 | 20.58 |
| San Marino | −35.72 | −38.39 | −4.49 |
| Serbia | 188.16 | 205.25 | 37.91 |
| Slovakia | 30.42 | 26.11 | 66.97 |
| Slovenia | −4.30 | −2.15 | −18.41 |
| Spain | −41.93 | −43.01 | −22.36 |
| Sweden | −44.13 | −45.80 | −16.53 |
| Switzerland | −59.89 | −61.85 | −39.92 |
| Ukraine | 122.17 | 130.69 | 51.16 |
| United Kingdom | −35.10 | −36.12 | −17.63 |

**Legend.**

Country‑specific Best Linear Unbiased Predictions (BLUPs) of the Estimated Annual Percentage Change (EAPC) in age‑standardized DALY, YLD, and YLL rates for ischemic stroke, derived from mixed‑effects models with random intercepts and random slopes (Both sexes, 1990–2023). Positive values indicate slower‑than‑average declines or relative increases compared with the continental trend, whereas negative values indicate faster‑than‑average reductions. BLUP‑EAPCs represent country‑level deviations from the fixed‑effect European slope.
