# Supplementary material for: Trends in mortality and disability from ischaemic stroke in Europe, 1990-2023
Source: Eur Stroke J. 2026 Jul 21;11(7):aakag082. doi: 10.1093/esj/aakag082 (PMC13387428; doi:10.1093/esj/aakag082)
Supplement: Supplementary_material_aakag082 [file supplementary_material_aakag082.zip › Supplementary Table 5.docx]

**Supplementary Table 5.** Country‑ and sex‑specific linear trends in Years of Life Lost (YLL) and Years Lived with Disability (YLD) for ischemic stroke, 1990–2023 (β coefficients, Standard error, p‑values)

*The table reports annual log‑linear coefficients (β), standard errors (SE), and p‑values from separate regressions of ln(YLL) and ln(YLD) on calendar year. Negative β values indicate declining trends, while positive values indicate increasing trends. All models use robust standard errors.*

| **Country** | **Sex** | **Beta_YLL** | **SE_YLL** | **p_YLL** | **Beta_YLD** | **SE_YLD** | **p_YLD** |
| --- | --- | --- | --- | --- | --- | --- | --- |
| Albania | Both | -0,0108 | 0,0010 | <0,0001 | 0,0108 | 0,0010 | <0,0001 |
| Albania | Female | -0,0143 | 0,0010 | <0,0001 | 0,0143 | 0,0010 | <0,0001 |
| Albania | Male | -0,0066 | 0,0011 | <0,0001 | 0,0066 | 0,0011 | <0,0001 |
| Andorra | Both | -0,0184 | 0,0010 | <0,0001 | 0,0184 | 0,0010 | <0,0001 |
| Andorra | Female | -0,0195 | 0,0012 | <0,0001 | 0,0195 | 0,0012 | <0,0001 |
| Andorra | Male | -0,0177 | 0,0008 | <0,0001 | 0,0177 | 0,0008 | <0,0001 |
| Austria | Both | -0,0544 | 0,0031 | <0,0001 | 0,0544 | 0,0031 | <0,0001 |
| Austria | Female | -0,0570 | 0,0029 | <0,0001 | 0,0570 | 0,0029 | <0,0001 |
| Austria | Male | -0,0513 | 0,0033 | <0,0001 | 0,0513 | 0,0033 | <0,0001 |
| Belarus | Both | -0,0155 | 0,0014 | <0,0001 | 0,0155 | 0,0014 | <0,0001 |
| Belarus | Female | -0,0166 | 0,0014 | <0,0001 | 0,0166 | 0,0014 | <0,0001 |
| Belarus | Male | -0,0139 | 0,0013 | <0,0001 | 0,0139 | 0,0013 | <0,0001 |
| Belgium | Both | -0,0306 | 0,0008 | <0,0001 | 0,0306 | 0,0008 | <0,0001 |
| Belgium | Female | -0,0306 | 0,0009 | <0,0001 | 0,0306 | 0,0009 | <0,0001 |
| Belgium | Male | -0,0311 | 0,0008 | <0,0001 | 0,0311 | 0,0008 | <0,0001 |
| Bosnia and Herzegovina | Both | -0,0060 | 0,0006 | <0,0001 | 0,0060 | 0,0006 | <0,0001 |
| Bosnia and Herzegovina | Female | -0,0084 | 0,0004 | <0,0001 | 0,0084 | 0,0004 | <0,0001 |
| Bosnia and Herzegovina | Male | -0,0032 | 0,0008 | <0,0001 | 0,0032 | 0,0008 | <0,0001 |
| Bulgaria | Both | -0,0045 | 0,0004 | <0,0001 | 0,0045 | 0,0004 | <0,0001 |
| Bulgaria | Female | -0,0071 | 0,0004 | <0,0001 | 0,0071 | 0,0004 | <0,0001 |
| Bulgaria | Male | -0,0022 | 0,0005 | <0,0001 | 0,0022 | 0,0005 | <0,0001 |
| Croatia | Both | -0,0261 | 0,0010 | <0,0001 | 0,0261 | 0,0010 | <0,0001 |
| Croatia | Female | -0,0254 | 0,0010 | <0,0001 | 0,0254 | 0,0010 | <0,0001 |
| Croatia | Male | -0,0270 | 0,0010 | <0,0001 | 0,0270 | 0,0010 | <0,0001 |
| Cyprus | Both | -0,0351 | 0,0006 | <0,0001 | 0,0351 | 0,0006 | <0,0001 |
| Cyprus | Female | -0,0392 | 0,0006 | <0,0001 | 0,0392 | 0,0006 | <0,0001 |
| Cyprus | Male | -0,0313 | 0,0007 | <0,0001 | 0,0313 | 0,0007 | <0,0001 |
| Czechia | Both | -0,0412 | 0,0015 | <0,0001 | 0,0412 | 0,0015 | <0,0001 |
| Czechia | Female | -0,0435 | 0,0016 | <0,0001 | 0,0435 | 0,0016 | <0,0001 |
| Czechia | Male | -0,0395 | 0,0015 | <0,0001 | 0,0395 | 0,0015 | <0,0001 |
| Denmark | Both | -0,0224 | 0,0008 | <0,0001 | 0,0224 | 0,0008 | <0,0001 |
| Denmark | Female | -0,0242 | 0,0008 | <0,0001 | 0,0242 | 0,0008 | <0,0001 |
| Denmark | Male | -0,0209 | 0,0009 | <0,0001 | 0,0209 | 0,0009 | <0,0001 |
| Estonia | Both | -0,0520 | 0,0029 | <0,0001 | 0,0520 | 0,0029 | <0,0001 |
| Estonia | Female | -0,0615 | 0,0030 | <0,0001 | 0,0615 | 0,0030 | <0,0001 |
| Estonia | Male | -0,0436 | 0,0026 | <0,0001 | 0,0436 | 0,0026 | <0,0001 |
| Finland | Both | -0,0277 | 0,0021 | <0,0001 | 0,0277 | 0,0021 | <0,0001 |
| Finland | Female | -0,0297 | 0,0022 | <0,0001 | 0,0297 | 0,0022 | <0,0001 |
| Finland | Male | -0,0252 | 0,0020 | <0,0001 | 0,0252 | 0,0020 | <0,0001 |
| France | Both | -0,0363 | 0,0011 | <0,0001 | 0,0363 | 0,0011 | <0,0001 |
| France | Female | -0,0357 | 0,0011 | <0,0001 | 0,0357 | 0,0011 | <0,0001 |
| France | Male | -0,0379 | 0,0011 | <0,0001 | 0,0379 | 0,0011 | <0,0001 |
| Germany | Both | -0,0399 | 0,0019 | <0,0001 | 0,0399 | 0,0019 | <0,0001 |
| Germany | Female | -0,0385 | 0,0016 | <0,0001 | 0,0385 | 0,0016 | <0,0001 |
| Germany | Male | -0,0426 | 0,0023 | <0,0001 | 0,0426 | 0,0023 | <0,0001 |
| Greece | Both | -0,0333 | 0,0014 | <0,0001 | 0,0333 | 0,0014 | <0,0001 |
| Greece | Female | -0,0358 | 0,0015 | <0,0001 | 0,0358 | 0,0015 | <0,0001 |
| Greece | Male | -0,0306 | 0,0012 | <0,0001 | 0,0306 | 0,0012 | <0,0001 |
| Hungary | Both | -0,0216 | 0,0013 | <0,0001 | 0,0216 | 0,0013 | <0,0001 |
| Hungary | Female | -0,0238 | 0,0015 | <0,0001 | 0,0238 | 0,0015 | <0,0001 |
| Hungary | Male | -0,0205 | 0,0010 | <0,0001 | 0,0205 | 0,0010 | <0,0001 |
| Iceland | Both | -0,0260 | 0,0008 | <0,0001 | 0,0260 | 0,0008 | <0,0001 |
| Iceland | Female | -0,0246 | 0,0010 | <0,0001 | 0,0246 | 0,0010 | <0,0001 |
| Iceland | Male | -0,0279 | 0,0010 | <0,0001 | 0,0279 | 0,0010 | <0,0001 |
| Ireland | Both | -0,0353 | 0,0012 | <0,0001 | 0,0353 | 0,0012 | <0,0001 |
| Ireland | Female | -0,0361 | 0,0011 | <0,0001 | 0,0361 | 0,0011 | <0,0001 |
| Ireland | Male | -0,0346 | 0,0013 | <0,0001 | 0,0346 | 0,0013 | <0,0001 |
| Israel | Both | -0,0292 | 0,0018 | <0,0001 | 0,0292 | 0,0018 | <0,0001 |
| Israel | Female | -0,0329 | 0,0013 | <0,0001 | 0,0329 | 0,0013 | <0,0001 |
| Israel | Male | -0,0268 | 0,0024 | <0,0001 | 0,0268 | 0,0024 | <0,0001 |
| Italy | Both | -0,0359 | 0,0013 | <0,0001 | 0,0359 | 0,0013 | <0,0001 |
| Italy | Female | -0,0337 | 0,0012 | <0,0001 | 0,0337 | 0,0012 | <0,0001 |
| Italy | Male | -0,0385 | 0,0013 | <0,0001 | 0,0385 | 0,0013 | <0,0001 |
| Latvia | Both | -0,0108 | 0,0010 | <0,0001 | 0,0108 | 0,0010 | <0,0001 |
| Latvia | Female | -0,0123 | 0,0011 | <0,0001 | 0,0123 | 0,0011 | <0,0001 |
| Latvia | Male | -0,0099 | 0,0011 | <0,0001 | 0,0099 | 0,0011 | <0,0001 |
| Lithuania | Both | -0,0103 | 0,0024 | <0,0001 | 0,0103 | 0,0024 | <0,0001 |
| Lithuania | Female | -0,0122 | 0,0023 | <0,0001 | 0,0122 | 0,0023 | <0,0001 |
| Lithuania | Male | -0,0101 | 0,0026 | <0,0001 | 0,0101 | 0,0026 | <0,0001 |
| Luxembourg | Both | -0,0375 | 0,0009 | <0,0001 | 0,0375 | 0,0009 | <0,0001 |
| Luxembourg | Female | -0,0388 | 0,0011 | <0,0001 | 0,0388 | 0,0011 | <0,0001 |
| Luxembourg | Male | -0,0361 | 0,0009 | <0,0001 | 0,0361 | 0,0009 | <0,0001 |
| Malta | Both | -0,0308 | 0,0016 | <0,0001 | 0,0308 | 0,0016 | <0,0001 |
| Malta | Female | -0,0349 | 0,0019 | <0,0001 | 0,0349 | 0,0019 | <0,0001 |
| Malta | Male | -0,0264 | 0,0014 | <0,0001 | 0,0264 | 0,0014 | <0,0001 |
| Monaco | Both | -0,0295 | 0,0018 | <0,0001 | 0,0295 | 0,0018 | <0,0001 |
| Monaco | Female | -0,0341 | 0,0013 | <0,0001 | 0,0341 | 0,0013 | <0,0001 |
| Monaco | Male | -0,0265 | 0,0024 | <0,0001 | 0,0265 | 0,0024 | <0,0001 |
| Montenegro | Both | -0,0084 | 0,0011 | <0,0001 | 0,0084 | 0,0011 | <0,0001 |
| Montenegro | Female | -0,0085 | 0,0011 | <0,0001 | 0,0085 | 0,0011 | <0,0001 |
| Montenegro | Male | -0,0078 | 0,0010 | <0,0001 | 0,0078 | 0,0010 | <0,0001 |
| Netherlands | Both | -0,0220 | 0,0015 | <0,0001 | 0,0220 | 0,0015 | <0,0001 |
| Netherlands | Female | -0,0216 | 0,0016 | <0,0001 | 0,0216 | 0,0016 | <0,0001 |
| Netherlands | Male | -0,0226 | 0,0015 | <0,0001 | 0,0226 | 0,0015 | <0,0001 |
| North Macedonia | Both | -0,0100 | 0,0009 | <0,0001 | 0,0100 | 0,0009 | <0,0001 |
| North Macedonia | Female | -0,0075 | 0,0009 | <0,0001 | 0,0075 | 0,0009 | <0,0001 |
| North Macedonia | Male | -0,0128 | 0,0010 | <0,0001 | 0,0128 | 0,0010 | <0,0001 |
| Norway | Both | -0,0389 | 0,0007 | <0,0001 | 0,0389 | 0,0007 | <0,0001 |
| Norway | Female | -0,0377 | 0,0008 | <0,0001 | 0,0377 | 0,0008 | <0,0001 |
| Norway | Male | -0,0396 | 0,0007 | <0,0001 | 0,0396 | 0,0007 | <0,0001 |
| Poland | Both | -0,0377 | 0,0006 | <0,0001 | 0,0377 | 0,0006 | <0,0001 |
| Poland | Female | -0,0391 | 0,0008 | <0,0001 | 0,0391 | 0,0008 | <0,0001 |
| Poland | Male | -0,0373 | 0,0006 | <0,0001 | 0,0373 | 0,0006 | <0,0001 |
| Portugal | Both | -0,0359 | 0,0014 | <0,0001 | 0,0359 | 0,0014 | <0,0001 |
| Portugal | Female | -0,0335 | 0,0017 | <0,0001 | 0,0335 | 0,0017 | <0,0001 |
| Portugal | Male | -0,0397 | 0,0010 | <0,0001 | 0,0397 | 0,0010 | <0,0001 |
| Republic of Moldova | Both | -0,0093 | 0,0026 | 0,001 | 0,0093 | 0,0026 | 0,001 |
| Republic of Moldova | Female | -0,0121 | 0,0028 | <0,0001 | 0,0121 | 0,0028 | <0,0001 |
| Republic of Moldova | Male | -0,0071 | 0,0025 | 0,008 | 0,0071 | 0,0025 | 0,008 |
| Romania | Both | -0,0104 | 0,0009 | <0,0001 | 0,0104 | 0,0009 | <0,0001 |
| Romania | Female | -0,0122 | 0,0009 | <0,0001 | 0,0122 | 0,0009 | <0,0001 |
| Romania | Male | -0,0090 | 0,0009 | <0,0001 | 0,0090 | 0,0009 | <0,0001 |
| Russian Federation | Both | -0,0242 | 0,0020 | <0,0001 | 0,0242 | 0,0020 | <0,0001 |
| Russian Federation | Female | -0,0247 | 0,0019 | <0,0001 | 0,0247 | 0,0019 | <0,0001 |
| Russian Federation | Male | -0,0258 | 0,0022 | <0,0001 | 0,0258 | 0,0022 | <0,0001 |
| San Marino | Both | -0,0329 | 0,0007 | <0,0001 | 0,0329 | 0,0007 | <0,0001 |
| San Marino | Female | -0,0328 | 0,0009 | <0,0001 | 0,0328 | 0,0009 | <0,0001 |
| San Marino | Male | -0,0341 | 0,0007 | <0,0001 | 0,0341 | 0,0007 | <0,0001 |
| Serbia | Both | -0,0128 | 0,0007 | <0,0001 | 0,0128 | 0,0007 | <0,0001 |
| Serbia | Female | -0,0141 | 0,0007 | <0,0001 | 0,0141 | 0,0007 | <0,0001 |
| Serbia | Male | -0,0118 | 0,0007 | <0,0001 | 0,0118 | 0,0007 | <0,0001 |
| Slovakia | Both | -0,0142 | 0,0010 | <0,0001 | 0,0142 | 0,0010 | <0,0001 |
| Slovakia | Female | -0,0200 | 0,0009 | <0,0001 | 0,0200 | 0,0009 | <0,0001 |
| Slovakia | Male | -0,0092 | 0,0011 | <0,0001 | 0,0092 | 0,0011 | <0,0001 |
| Slovenia | Both | -0,0314 | 0,0012 | <0,0001 | 0,0314 | 0,0012 | <0,0001 |
| Slovenia | Female | -0,0343 | 0,0010 | <0,0001 | 0,0343 | 0,0010 | <0,0001 |
| Slovenia | Male | -0,0290 | 0,0014 | <0,0001 | 0,0290 | 0,0014 | <0,0001 |
| Spain | Both | -0,0434 | 0,0015 | <0,0001 | 0,0434 | 0,0015 | <0,0001 |
| Spain | Female | -0,0398 | 0,0011 | <0,0001 | 0,0398 | 0,0011 | <0,0001 |
| Spain | Male | -0,0463 | 0,0020 | <0,0001 | 0,0463 | 0,0020 | <0,0001 |
| Sweden | Both | -0,0360 | 0,0016 | <0,0001 | 0,0360 | 0,0016 | <0,0001 |
| Sweden | Female | -0,0363 | 0,0016 | <0,0001 | 0,0363 | 0,0016 | <0,0001 |
| Sweden | Male | -0,0352 | 0,0015 | <0,0001 | 0,0352 | 0,0015 | <0,0001 |
| Switzerland | Both | -0,0297 | 0,0010 | <0,0001 | 0,0297 | 0,0010 | <0,0001 |
| Switzerland | Female | -0,0298 | 0,0009 | <0,0001 | 0,0298 | 0,0009 | <0,0001 |
| Switzerland | Male | -0,0300 | 0,0010 | <0,0001 | 0,0300 | 0,0010 | <0,0001 |
| Ukraine | Both | -0,0203 | 0,0009 | <0,0001 | 0,0203 | 0,0009 | <0,0001 |
| Ukraine | Female | -0,0236 | 0,0008 | <0,0001 | 0,0236 | 0,0008 | <0,0001 |
| Ukraine | Male | -0,0156 | 0,0009 | <0,0001 | 0,0156 | 0,0009 | <0,0001 |
| United Kingdom | Both | -0,0348 | 0,0011 | <0,0001 | 0,0348 | 0,0011 | <0,0001 |
| United Kingdom | Female | -0,0359 | 0,0011 | <0,0001 | 0,0359 | 0,0011 | <0,0001 |
| United Kingdom | Male | -0,0334 | 0,0011 | <0,0001 | 0,0334 | 0,0011 | <0,0001 |
