# Supplementary material for: Trends in mortality and disability from ischaemic stroke in Europe, 1990-2023
Source: Eur Stroke J. 2026 Jul 21;11(7):aakag082. doi: 10.1093/esj/aakag082 (PMC13387428; doi:10.1093/esj/aakag082)
Supplement: Supplementary_material_aakag082 [file supplementary_material_aakag082.zip › Supplementary Table 6.docx]

**Supplementary Table 6.** Decomposition of changes in DALYs into YLL and YLD components by country and sex, 1990–2023

*ΔDALY, ΔYLL and ΔYLD represent the absolute annual changes in age‑standardized DALY, YLL and YLD rates. contrib\_YLL and contrib\_YLD denote the proportional contribution of mortality (YLL) and disability (YLD) to the total change in DALYs, calculated as ΔYLL/ΔDALY and ΔYLD/ΔDALY, respectively. Values may exceed 1 or fall below 0 when the components move in different directions or when ΔDALY is small relative to ΔYLL and ΔYLD, and therefore the two contributions do not necessarily sum to 1.*

| **Country** | **Sex** | **ΔDALY** | **ΔYLL** | **ΔYLD** | **contrib_YLL** | **contrib_YLD** |
| --- | --- | --- | --- | --- | --- | --- |
| Albania | Both | 427,8 | 397,8 | 33,0 | 0,93 | 0,08 |
| Albania | Female | 372,4 | 352,9 | 21,5 | 0,95 | 0,06 |
| Albania | Male | 500,0 | 452,5 | 47,9 | 0,90 | 0,10 |
| Andorra | Both | 299,1 | 257,9 | 41,7 | 0,86 | 0,14 |
| Andorra | Female | 296,3 | 260,6 | 37,4 | 0,88 | 0,13 |
| Andorra | Male | 300,9 | 255,7 | 45,5 | 0,85 | 0,15 |
| Austria | Both | 758,8 | 745,6 | 30,5 | 0,98 | 0,04 |
| Austria | Female | 660,4 | 652,6 | 19,7 | 0,99 | 0,03 |
| Austria | Male | 938,9 | 910,6 | 51,4 | 0,97 | 0,05 |
| Belarus | Both | 1343,4 | 1297,3 | 53,8 | 0,97 | 0,04 |
| Belarus | Female | 1057,8 | 1018,4 | 48,2 | 0,96 | 0,05 |
| Belarus | Male | 1756,6 | 1695,4 | 66,7 | 0,97 | 0,04 |
| Belgium | Both | 620,6 | 588,1 | 33,5 | 0,95 | 0,05 |
| Belgium | Female | 557,9 | 531,0 | 31,1 | 0,95 | 0,06 |
| Belgium | Male | 724,4 | 683,3 | 41,5 | 0,94 | 0,06 |
| Bosnia and Herzegovina | Both | 999,0 | 945,6 | 53,9 | 0,95 | 0,05 |
| Bosnia and Herzegovina | Female | 1004,7 | 962,4 | 42,9 | 0,96 | 0,04 |
| Bosnia and Herzegovina | Male | 1009,9 | 937,6 | 72,7 | 0,93 | 0,07 |
| Bulgaria | Both | 896,4 | 854,6 | 44,9 | 0,95 | 0,05 |
| Bulgaria | Female | 884,1 | 848,1 | 39,7 | 0,96 | 0,04 |
| Bulgaria | Male | 957,0 | 905,6 | 52,5 | 0,95 | 0,05 |
| Croatia | Both | 1318,8 | 1267,9 | 51,2 | 0,96 | 0,04 |
| Croatia | Female | 1200,3 | 1152,2 | 48,3 | 0,96 | 0,04 |
| Croatia | Male | 1531,2 | 1469,7 | 61,8 | 0,96 | 0,04 |
| Cyprus | Both | 951,7 | 927,3 | 26,9 | 0,97 | 0,03 |
| Cyprus | Female | 943,3 | 929,0 | 17,2 | 0,98 | 0,02 |
| Cyprus | Male | 955,5 | 918,8 | 38,3 | 0,96 | 0,04 |
| Czechia | Both | 2140,9 | 2045,5 | 95,4 | 0,96 | 0,04 |
| Czechia | Female | 1869,6 | 1787,8 | 81,8 | 0,96 | 0,04 |
| Czechia | Male | 2568,9 | 2448,7 | 120,6 | 0,95 | 0,05 |
| Denmark | Both | 522,6 | 474,3 | 48,7 | 0,91 | 0,09 |
| Denmark | Female | 462,4 | 422,2 | 40,5 | 0,91 | 0,09 |
| Denmark | Male | 612,5 | 550,3 | 62,9 | 0,90 | 0,10 |
| Estonia | Both | 1970,3 | 1912,9 | 69,4 | 0,97 | 0,04 |
| Estonia | Female | 1771,5 | 1713,2 | 61,6 | 0,97 | 0,03 |
| Estonia | Male | 2316,7 | 2249,1 | 83,7 | 0,97 | 0,04 |
| Finland | Both | 703,3 | 655,3 | 49,1 | 0,93 | 0,07 |
| Finland | Female | 592,7 | 556,8 | 38,4 | 0,94 | 0,06 |
| Finland | Male | 872,1 | 798,2 | 74,9 | 0,92 | 0,09 |
| France | Both | 359,8 | 362,0 | 5,1 | 1,01 | 0,01 |
| France | Female | 300,5 | 301,2 | 5,4 | 1,00 | 0,02 |
| France | Male | 463,0 | 464,6 | 5,1 | 1,00 | 0,01 |
| Germany | Both | 701,1 | 689,2 | 14,7 | 0,98 | 0,02 |
| Germany | Female | 621,2 | 600,0 | 23,5 | 0,97 | 0,04 |
| Germany | Male | 870,1 | 862,9 | 12,4 | 0,99 | 0,01 |
| Greece | Both | 1046,8 | 993,3 | 54,0 | 0,95 | 0,05 |
| Greece | Female | 1102,7 | 1052,0 | 50,9 | 0,95 | 0,05 |
| Greece | Male | 978,7 | 920,8 | 58,4 | 0,94 | 0,06 |
| Hungary | Both | 1516,3 | 1426,1 | 90,5 | 0,94 | 0,06 |
| Hungary | Female | 1304,5 | 1225,4 | 79,8 | 0,94 | 0,06 |
| Hungary | Male | 1813,0 | 1708,2 | 105,0 | 0,94 | 0,06 |
| Iceland | Both | 430,3 | 392,7 | 38,2 | 0,91 | 0,09 |
| Iceland | Female | 338,3 | 309,8 | 28,7 | 0,92 | 0,08 |
| Iceland | Male | 558,0 | 510,6 | 52,0 | 0,92 | 0,09 |
| Ireland | Both | 727,5 | 683,7 | 45,1 | 0,94 | 0,06 |
| Ireland | Female | 645,8 | 610,9 | 36,7 | 0,95 | 0,06 |
| Ireland | Male | 824,3 | 767,1 | 58,3 | 0,93 | 0,07 |
| Israel | Both | 451,1 | 392,5 | 58,8 | 0,87 | 0,13 |
| Israel | Female | 402,7 | 364,2 | 38,7 | 0,90 | 0,10 |
| Israel | Male | 514,6 | 430,6 | 84,2 | 0,84 | 0,16 |
| Italy | Both | 667,9 | 648,3 | 19,6 | 0,97 | 0,03 |
| Italy | Female | 576,7 | 557,2 | 19,6 | 0,97 | 0,03 |
| Italy | Male | 818,9 | 796,7 | 22,6 | 0,97 | 0,03 |
| Latvia | Both | 1306,3 | 1242,5 | 64,8 | 0,95 | 0,05 |
| Latvia | Female | 1193,6 | 1132,1 | 62,4 | 0,95 | 0,05 |
| Latvia | Male | 1550,3 | 1477,4 | 73,6 | 0,95 | 0,05 |
| Lithuania | Both | 841,6 | 819,4 | 96,8 | 0,97 | 0,12 |
| Lithuania | Female | 856,6 | 817,6 | 110,2 | 0,95 | 0,13 |
| Lithuania | Male | 826,6 | 832,4 | 84,3 | 1,01 | 0,10 |
| Luxembourg | Both | 1121,9 | 1072,9 | 50,1 | 0,96 | 0,04 |
| Luxembourg | Female | 1011,6 | 968,1 | 44,7 | 0,96 | 0,04 |
| Luxembourg | Male | 1290,7 | 1230,4 | 61,2 | 0,95 | 0,05 |
| Malta | Both | 767,5 | 726,3 | 41,9 | 0,95 | 0,05 |
| Malta | Female | 729,5 | 698,4 | 32,5 | 0,96 | 0,04 |
| Malta | Male | 826,5 | 770,5 | 57,2 | 0,93 | 0,07 |
| Monaco | Both | 1097,9 | 1026,1 | 72,7 | 0,93 | 0,07 |
| Monaco | Female | 976,6 | 933,6 | 43,3 | 0,96 | 0,04 |
| Monaco | Male | 1252,8 | 1142,2 | 111,5 | 0,91 | 0,09 |
| Montenegro | Both | 1021,2 | 975,1 | 51,2 | 0,95 | 0,05 |
| Montenegro | Female | 1021,2 | 983,3 | 43,4 | 0,96 | 0,04 |
| Montenegro | Male | 1022,4 | 962,0 | 62,3 | 0,94 | 0,06 |
| Netherlands | Both | 446,2 | 403,4 | 43,3 | 0,90 | 0,10 |
| Netherlands | Female | 393,1 | 355,0 | 39,7 | 0,90 | 0,10 |
| Netherlands | Male | 533,9 | 479,5 | 54,8 | 0,90 | 0,10 |
| North Macedonia | Both | 1458,9 | 1405,8 | 53,9 | 0,96 | 0,04 |
| North Macedonia | Female | 1412,7 | 1361,9 | 60,1 | 0,96 | 0,04 |
| North Macedonia | Male | 1553,0 | 1510,4 | 43,1 | 0,97 | 0,03 |
| Norway | Both | 666,7 | 629,9 | 39,6 | 0,94 | 0,06 |
| Norway | Female | 590,2 | 559,8 | 32,9 | 0,95 | 0,06 |
| Norway | Male | 792,3 | 738,8 | 54,1 | 0,93 | 0,07 |
| Poland | Both | 1393,6 | 1386,6 | 11,9 | 1,00 | 0,01 |
| Poland | Female | 1313,4 | 1299,2 | 18,6 | 0,99 | 0,01 |
| Poland | Male | 1508,8 | 1510,6 | 7,1 | 1,00 | 0,00 |
| Portugal | Both | 1872,3 | 1770,0 | 103,0 | 0,95 | 0,06 |
| Portugal | Female | 1695,5 | 1584,9 | 111,1 | 0,93 | 0,07 |
| Portugal | Male | 2120,9 | 2030,5 | 91,8 | 0,96 | 0,04 |
| Republic of Moldova | Both | 753,1 | 739,4 | 21,7 | 0,98 | 0,03 |
| Republic of Moldova | Female | 842,4 | 817,8 | 25,3 | 0,97 | 0,03 |
| Republic of Moldova | Male | 759,0 | 751,8 | 17,6 | 0,99 | 0,02 |
| Romania | Both | 1474,4 | 1405,7 | 69,4 | 0,95 | 0,05 |
| Romania | Female | 1354,5 | 1295,8 | 59,6 | 0,96 | 0,04 |
| Romania | Male | 1612,8 | 1532,8 | 80,7 | 0,95 | 0,05 |
| Russian Federation | Both | 2498,0 | 2465,1 | 34,4 | 0,99 | 0,01 |
| Russian Federation | Female | 2269,6 | 2225,9 | 44,0 | 0,98 | 0,02 |
| Russian Federation | Male | 3021,0 | 3003,0 | 25,1 | 0,99 | 0,01 |
| San Marino | Both | 710,2 | 662,6 | 48,7 | 0,93 | 0,07 |
| San Marino | Female | 542,2 | 512,4 | 30,8 | 0,95 | 0,06 |
| San Marino | Male | 953,0 | 881,2 | 74,1 | 0,92 | 0,08 |
| Serbia | Both | 2141,3 | 2071,0 | 72,2 | 0,97 | 0,03 |
| Serbia | Female | 2070,6 | 2012,6 | 59,0 | 0,97 | 0,03 |
| Serbia | Male | 2227,3 | 2138,9 | 89,6 | 0,96 | 0,04 |
| Slovakia | Both | 1161,6 | 1064,4 | 108,5 | 0,92 | 0,09 |
| Slovakia | Female | 1042,9 | 966,0 | 87,5 | 0,93 | 0,08 |
| Slovakia | Male | 1329,0 | 1200,3 | 145,3 | 0,90 | 0,11 |
| Slovenia | Both | 1187,3 | 1142,3 | 45,4 | 0,96 | 0,04 |
| Slovenia | Female | 989,6 | 953,2 | 36,7 | 0,96 | 0,04 |
| Slovenia | Male | 1552,1 | 1487,3 | 65,0 | 0,96 | 0,04 |
| Spain | Both | 752,0 | 721,2 | 30,9 | 0,96 | 0,04 |
| Spain | Female | 717,8 | 678,4 | 39,4 | 0,95 | 0,05 |
| Spain | Male | 801,2 | 779,8 | 21,8 | 0,97 | 0,03 |
| Sweden | Both | 489,9 | 470,7 | 21,8 | 0,96 | 0,04 |
| Sweden | Female | 426,7 | 413,7 | 15,3 | 0,97 | 0,04 |
| Sweden | Male | 583,6 | 549,6 | 36,3 | 0,94 | 0,06 |
| Switzerland | Both | 458,4 | 430,2 | 28,5 | 0,94 | 0,06 |
| Switzerland | Female | 391,1 | 368,3 | 23,3 | 0,94 | 0,06 |
| Switzerland | Male | 560,9 | 522,3 | 38,8 | 0,93 | 0,07 |
| Ukraine | Both | 2022,6 | 1957,1 | 66,2 | 0,97 | 0,03 |
| Ukraine | Female | 1795,4 | 1735,8 | 60,1 | 0,97 | 0,03 |
| Ukraine | Male | 2455,1 | 2365,8 | 95,5 | 0,96 | 0,04 |
| United Kingdom | Both | 687,5 | 645,8 | 43,7 | 0,94 | 0,06 |
| United Kingdom | Female | 606,6 | 571,7 | 35,8 | 0,94 | 0,06 |
| United Kingdom | Male | 796,0 | 742,3 | 56,7 | 0,93 | 0,07 |
