# Supplementary material for: Trends in mortality and disability from ischaemic stroke in Europe, 1990-2023
Source: Eur Stroke J. 2026 Jul 21;11(7):aakag082. doi: 10.1093/esj/aakag082 (PMC13387428; doi:10.1093/esj/aakag082)
Supplement: Supplementary_material_aakag082 [file supplementary_material_aakag082.zip › Supplementary Material.docx]

**Supplementary Material**

**Supplementary Figure 1.** Estimated Annual Percentage Change (EAPC) in Years of Life Lost (YLLs) due to Ischemic Stroke Across Europe, 1990–2023

**Supplementary Figure 2.** Estimated Annual Percentage Change (EAPC) in Years Lived with Disability (YLDs) due to Ischemic Stroke Across Europe, 1990–2023

**Supplementary Figure 3.** Country‑specific deviations from the European DALY trend (BLUP‑EAPC). This map illustrates the Best Linear Unbiased Predictions (BLUP‑EAPCs) for DALY rates, quantifying each country’s deviation from the overall European trend. Negative values (blue shades) indicate faster‑than‑average reductions in disability‑adjusted life‑years, whereas positive values (red shades) reflect slower progress or relative increases.

**Supplementary Figure 4.** Country‑specific deviations from the European YLD trend (BLUP‑EAPC). This map shows the Best Linear Unbiased Predictions (BLUP‑EAPCs) for YLD rates, capturing each country’s deviation from the overall European trend in disability outcomes. Negative values (blue shades) indicate faster‑than‑average reductions in disability‑related burden, whereas positive values (red shades) reflect slower progress or relative increases.

**Supplementary Figure 5.** Country‑specific deviations from the European YLL trend (BLUP‑EAPC). This map displays the Best Linear Unbiased Predictions (BLUP‑EAPCs) for YLL rates, representing each country’s deviation from the overall European trend. Negative values (blue shades) indicate faster‑than‑average declines in premature mortality, while positive values (red shades) reflect slower progress or relative increases.

**Supplementary Figure 6.** Forest plot of the Estimated Annual Percentage Change (EAPC) in DALYs for Ischemic Stroke Across European Countries (1990–2023), Showing Fixed‑Effects (red dashed vertical line) and Random‑Effects (blue dashed vertical line) Pooled Estimates

**Supplementary Figure 7.** Forest plot of the Estimated Annual Percentage Change (EAPC) in Years of Life Lost (YLLs) for Ischemic Stroke Across European Countries (1990–2023), Showing Fixed‑Effects (red dashed vertical line) and Random‑Effects (blue dashed vertical line) Pooled Estimates

**Supplementary Figure 8.** Forest plot of the Estimated Annual Percentage Change (EAPC) in Years Lived with Disability (YLDs) for Ischemic Stroke Across European Countries (1990–2023), showing Pooled Estimates (dashed vertical line)
