# Supplementary material for: Trends in mortality and disability from ischaemic stroke in Europe, 1990-2023
Source: Eur Stroke J. 2026 Jul 21;11(7):aakag082. doi: 10.1093/esj/aakag082 (PMC13387428; doi:10.1093/esj/aakag082)
Supplement: Supplementary_material_aakag082 [file supplementary_material_aakag082.zip › Supplementary Table 1.docx]

**Supplementary Table 1.** Estimated Annual Percentage Change (EAPC), 95% Confidence Intervals for DALYs, YLLSs, and YLDs by Country and Sex, Europe, 1990–2023

*EAPCs were derived from log‑linear regressions of age‑standardized rates on calendar year. Negative values indicate declining trends, while positive values indicate increasing trends.*

| **Measure** | **Sex** | **Country** | **EAPC** | **Lower 95%CI** | **Upper 95% CI** |
| --- | --- | --- | --- | --- | --- |
| DALYs | Both | Albania | -1,84 | -1,98 | -1,70 |
| DALYs | Both | Andorra | -2,84 | -3,01 | -2,67 |
| DALYs | Both | Austria | -4,18 | -4,50 | -3,86 |
| DALYs | Both | Belarus | -2,47 | -2,88 | -2,06 |
| DALYs | Both | Belgium | -3,58 | -3,73 | -3,42 |
| DALYs | Both | Bosnia and Herzegovina | -1,52 | -1,65 | -1,40 |
| DALYs | Both | Bulgaria | -1,07 | -1,15 | -1,00 |
| DALYs | Both | Croatia | -3,43 | -3,70 | -3,17 |
| DALYs | Both | Cyprus | -4,31 | -4,50 | -4,12 |
| DALYs | Both | Czechia | -5,35 | -5,61 | -5,09 |
| DALYs | Both | Denmark | -3,31 | -3,49 | -3,13 |
| DALYs | Both | Estonia | -6,04 | -6,62 | -5,46 |
| DALYs | Both | Finland | -3,37 | -3,53 | -3,21 |
| DALYs | Both | France | -2,65 | -2,82 | -2,47 |
| DALYs | Both | Germany | -3,25 | -3,60 | -2,91 |
| DALYs | Both | Greece | -4,40 | -4,65 | -4,15 |
| DALYs | Both | Hungary | -3,59 | -3,80 | -3,38 |
| DALYs | Both | Iceland | -3,37 | -3,54 | -3,20 |
| DALYs | Both | Ireland | -4,77 | -4,93 | -4,60 |
| DALYs | Both | Israel | -3,65 | -3,82 | -3,49 |
| DALYs | Both | Italy | -3,83 | -4,12 | -3,53 |
| DALYs | Both | Latvia | -2,20 | -2,43 | -1,98 |
| DALYs | Both | Lithuania | -1,72 | -2,06 | -1,38 |
| DALYs | Both | Luxembourg | -5,20 | -5,47 | -4,94 |
| DALYs | Both | Malta | -4,40 | -4,53 | -4,27 |
| DALYs | Both | Monaco | -4,48 | -4,90 | -4,07 |
| DALYs | Both | Montenegro | -1,90 | -2,24 | -1,56 |
| DALYs | Both | Netherlands | -3,09 | -3,27 | -2,91 |
| DALYs | Both | North Macedonia | -1,75 | -2,04 | -1,47 |
| DALYs | Both | Norway | -4,10 | -4,26 | -3,94 |
| DALYs | Both | Poland | -3,49 | -3,61 | -3,37 |
| DALYs | Both | Portugal | -5,55 | -5,83 | -5,28 |
| DALYs | Both | Republic of Moldova | -1,17 | -1,64 | -0,70 |
| DALYs | Both | Romania | -2,38 | -2,69 | -2,06 |
| DALYs | Both | Russian Federation | -2,89 | -3,44 | -2,34 |
| DALYs | Both | San Marino | -3,94 | -4,12 | -3,75 |
| DALYs | Both | Serbia | -2,69 | -2,94 | -2,44 |
| DALYs | Both | Slovakia | -3,03 | -3,20 | -2,87 |
| DALYs | Both | Slovenia | -4,15 | -4,39 | -3,92 |
| DALYs | Both | Spain | -4,18 | -4,51 | -3,85 |
| DALYs | Both | Sweden | -3,21 | -3,35 | -3,07 |
| DALYs | Both | Switzerland | -3,52 | -3,73 | -3,30 |
| DALYs | Both | Ukraine | -3,03 | -3,28 | -2,77 |
| DALYs | Both | United Kingdom | -4,24 | -4,48 | -4,01 |
| DALYs | Female | Albania | -1,75 | -1,90 | -1,61 |
| DALYs | Female | Andorra | -2,86 | -3,07 | -2,65 |
| DALYs | Female | Austria | -4,25 | -4,57 | -3,92 |
| DALYs | Female | Belarus | -2,53 | -2,90 | -2,17 |
| DALYs | Female | Belgium | -3,58 | -3,74 | -3,42 |
| DALYs | Female | Bosnia and Herzegovina | -1,67 | -1,80 | -1,55 |
| DALYs | Female | Bulgaria | -1,27 | -1,35 | -1,19 |
| DALYs | Female | Croatia | -3,59 | -3,86 | -3,32 |
| DALYs | Female | Cyprus | -4,42 | -4,61 | -4,22 |
| DALYs | Female | Czechia | -5,45 | -5,75 | -5,15 |
| DALYs | Female | Denmark | -3,34 | -3,52 | -3,17 |
| DALYs | Female | Estonia | -6,68 | -7,27 | -6,08 |
| DALYs | Female | Finland | -3,31 | -3,48 | -3,13 |
| DALYs | Female | France | -2,62 | -2,79 | -2,45 |
| DALYs | Female | Germany | -3,39 | -3,70 | -3,08 |
| DALYs | Female | Greece | -4,66 | -4,92 | -4,39 |
| DALYs | Female | Hungary | -3,75 | -3,95 | -3,54 |
| DALYs | Female | Iceland | -3,08 | -3,29 | -2,87 |
| DALYs | Female | Ireland | -4,69 | -4,85 | -4,53 |
| DALYs | Female | Israel | -3,65 | -3,81 | -3,50 |
| DALYs | Female | Italy | -3,78 | -4,06 | -3,50 |
| DALYs | Female | Latvia | -2,48 | -2,71 | -2,25 |
| DALYs | Female | Lithuania | -2,22 | -2,62 | -1,81 |
| DALYs | Female | Luxembourg | -5,29 | -5,58 | -5,00 |
| DALYs | Female | Malta | -4,57 | -4,74 | -4,41 |
| DALYs | Female | Monaco | -4,26 | -4,56 | -3,95 |
| DALYs | Female | Montenegro | -1,90 | -2,24 | -1,56 |
| DALYs | Female | Netherlands | -3,06 | -3,23 | -2,89 |
| DALYs | Female | North Macedonia | -1,80 | -2,11 | -1,48 |
| DALYs | Female | Norway | -4,12 | -4,31 | -3,93 |
| DALYs | Female | Poland | -3,89 | -4,02 | -3,76 |
| DALYs | Female | Portugal | -5,66 | -5,92 | -5,41 |
| DALYs | Female | Republic of Moldova | -1,69 | -2,18 | -1,20 |
| DALYs | Female | Romania | -2,60 | -2,90 | -2,29 |
| DALYs | Female | Russian Federation | -3,19 | -3,73 | -2,65 |
| DALYs | Female | San Marino | -3,56 | -3,75 | -3,37 |
| DALYs | Female | Serbia | -2,76 | -3,02 | -2,50 |
| DALYs | Female | Slovakia | -3,33 | -3,48 | -3,18 |
| DALYs | Female | Slovenia | -4,30 | -4,49 | -4,11 |
| DALYs | Female | Spain | -4,61 | -4,93 | -4,29 |
| DALYs | Female | Sweden | -3,21 | -3,37 | -3,04 |
| DALYs | Female | Switzerland | -3,48 | -3,69 | -3,27 |
| DALYs | Female | Ukraine | -3,25 | -3,50 | -3,00 |
| DALYs | Female | United Kingdom | -4,18 | -4,40 | -3,95 |
| DALYs | Male | Albania | -1,91 | -2,06 | -1,76 |
| DALYs | Male | Andorra | -2,81 | -2,95 | -2,67 |
| DALYs | Male | Austria | -4,29 | -4,61 | -3,96 |
| DALYs | Male | Belarus | -2,39 | -2,81 | -1,97 |
| DALYs | Male | Belgium | -3,69 | -3,84 | -3,53 |
| DALYs | Male | Bosnia and Herzegovina | -1,39 | -1,53 | -1,25 |
| DALYs | Male | Bulgaria | -0,90 | -0,98 | -0,81 |
| DALYs | Male | Croatia | -3,41 | -3,69 | -3,13 |
| DALYs | Male | Cyprus | -4,20 | -4,40 | -4,00 |
| DALYs | Male | Czechia | -5,38 | -5,61 | -5,14 |
| DALYs | Male | Denmark | -3,38 | -3,58 | -3,18 |
| DALYs | Male | Estonia | -5,40 | -5,95 | -4,86 |
| DALYs | Male | Finland | -3,56 | -3,72 | -3,40 |
| DALYs | Male | France | -2,82 | -3,01 | -2,64 |
| DALYs | Male | Germany | -3,34 | -3,73 | -2,96 |
| DALYs | Male | Greece | -4,11 | -4,36 | -3,87 |
| DALYs | Male | Hungary | -3,46 | -3,67 | -3,24 |
| DALYs | Male | Iceland | -3,72 | -3,89 | -3,55 |
| DALYs | Male | Ireland | -4,91 | -5,09 | -4,73 |
| DALYs | Male | Israel | -3,70 | -3,89 | -3,52 |
| DALYs | Male | Italy | -3,99 | -4,29 | -3,70 |
| DALYs | Male | Latvia | -1,99 | -2,22 | -1,77 |
| DALYs | Male | Lithuania | -1,21 | -1,49 | -0,93 |
| DALYs | Male | Luxembourg | -5,20 | -5,46 | -4,95 |
| DALYs | Male | Malta | -4,26 | -4,39 | -4,13 |
| DALYs | Male | Monaco | -4,83 | -5,37 | -4,29 |
| DALYs | Male | Montenegro | -1,92 | -2,26 | -1,58 |
| DALYs | Male | Netherlands | -3,25 | -3,46 | -3,04 |
| DALYs | Male | North Macedonia | -1,73 | -1,99 | -1,47 |
| DALYs | Male | Norway | -4,19 | -4,34 | -4,03 |
| DALYs | Male | Poland | -3,18 | -3,30 | -3,06 |
| DALYs | Male | Portugal | -5,49 | -5,80 | -5,17 |
| DALYs | Male | Republic of Moldova | -0,68 | -1,14 | -0,22 |
| DALYs | Male | Romania | -2,13 | -2,47 | -1,80 |
| DALYs | Male | Russian Federation | -2,70 | -3,25 | -2,16 |
| DALYs | Male | San Marino | -4,39 | -4,59 | -4,19 |
| DALYs | Male | Serbia | -2,67 | -2,91 | -2,42 |
| DALYs | Male | Slovakia | -2,81 | -3,01 | -2,61 |
| DALYs | Male | Slovenia | -4,21 | -4,49 | -3,92 |
| DALYs | Male | Spain | -3,84 | -4,18 | -3,49 |
| DALYs | Male | Sweden | -3,28 | -3,42 | -3,15 |
| DALYs | Male | Switzerland | -3,67 | -3,88 | -3,46 |
| DALYs | Male | Ukraine | -2,84 | -3,08 | -2,60 |
| DALYs | Male | United Kingdom | -4,37 | -4,61 | -4,12 |
| YLDs | Both | Albania | -0,98 | -1,08 | -0,89 |
| YLDs | Both | Andorra | -1,57 | -1,68 | -1,46 |
| YLDs | Both | Austria | -0,21 | -0,43 | 0,02 |
| YLDs | Both | Belarus | -1,10 | -1,22 | -0,97 |
| YLDs | Both | Belgium | -1,20 | -1,26 | -1,13 |
| YLDs | Both | Bosnia and Herzegovina | -0,99 | -1,08 | -0,90 |
| YLDs | Both | Bulgaria | -0,67 | -0,71 | -0,63 |
| YLDs | Both | Croatia | -1,19 | -1,31 | -1,07 |
| YLDs | Both | Cyprus | -1,29 | -1,48 | -1,11 |
| YLDs | Both | Czechia | -1,94 | -2,13 | -1,75 |
| YLDs | Both | Denmark | -1,58 | -1,75 | -1,40 |
| YLDs | Both | Estonia | -1,73 | -1,90 | -1,56 |
| YLDs | Both | Finland | -1,25 | -1,47 | -1,04 |
| YLDs | Both | France | 0,07 | 0,00 | 0,13 |
| YLDs | Both | Germany | -0,31 | -0,34 | -0,28 |
| YLDs | Both | Greece | -1,64 | -1,73 | -1,54 |
| YLDs | Both | Hungary | -1,77 | -1,93 | -1,60 |
| YLDs | Both | Iceland | -1,42 | -1,51 | -1,33 |
| YLDs | Both | Ireland | -1,99 | -2,15 | -1,82 |
| YLDs | Both | Israel | -1,67 | -1,90 | -1,44 |
| YLDs | Both | Italy | -0,89 | -0,95 | -0,82 |
| YLDs | Both | Latvia | -1,24 | -1,36 | -1,12 |
| YLDs | Both | Lithuania | -0,83 | -1,20 | -0,46 |
| YLDs | Both | Luxembourg | -2,18 | -2,39 | -1,98 |
| YLDs | Both | Malta | -1,93 | -2,11 | -1,74 |
| YLDs | Both | Monaco | -2,13 | -2,32 | -1,93 |
| YLDs | Both | Montenegro | -1,15 | -1,26 | -1,04 |
| YLDs | Both | Netherlands | -1,40 | -1,51 | -1,30 |
| YLDs | Both | North Macedonia | -0,85 | -0,94 | -0,76 |
| YLDs | Both | Norway | -1,22 | -1,32 | -1,12 |
| YLDs | Both | Poland | -0,18 | -0,24 | -0,12 |
| YLDs | Both | Portugal | -2,62 | -2,82 | -2,43 |
| YLDs | Both | Republic of Moldova | -0,37 | -0,46 | -0,27 |
| YLDs | Both | Romania | -1,45 | -1,59 | -1,31 |
| YLDs | Both | Russian Federation | -0,68 | -0,78 | -0,58 |
| YLDs | Both | San Marino | -1,49 | -1,59 | -1,38 |
| YLDs | Both | Serbia | -1,52 | -1,63 | -1,41 |
| YLDs | Both | Slovakia | -1,86 | -2,17 | -1,55 |
| YLDs | Both | Slovenia | -1,52 | -1,60 | -1,43 |
| YLDs | Both | Spain | -0,95 | -1,00 | -0,90 |
| YLDs | Both | Sweden | -0,59 | -0,71 | -0,47 |
| YLDs | Both | Switzerland | -1,26 | -1,34 | -1,19 |
| YLDs | Both | Ukraine | -1,23 | -1,35 | -1,10 |
| YLDs | Both | United Kingdom | -1,58 | -1,69 | -1,46 |
| YLDs | Female | Albania | -0,64 | -0,71 | -0,58 |
| YLDs | Female | Andorra | -1,50 | -1,60 | -1,39 |
| YLDs | Female | Austria | -0,10 | -0,28 | 0,08 |
| YLDs | Female | Belarus | -1,08 | -1,18 | -0,98 |
| YLDs | Female | Belgium | -1,22 | -1,30 | -1,13 |
| YLDs | Female | Bosnia and Herzegovina | -0,92 | -1,03 | -0,82 |
| YLDs | Female | Bulgaria | -0,64 | -0,69 | -0,58 |
| YLDs | Female | Croatia | -1,40 | -1,50 | -1,30 |
| YLDs | Female | Cyprus | -0,99 | -1,15 | -0,82 |
| YLDs | Female | Czechia | -1,87 | -2,08 | -1,65 |
| YLDs | Female | Denmark | -1,48 | -1,66 | -1,31 |
| YLDs | Female | Estonia | -1,73 | -1,91 | -1,55 |
| YLDs | Female | Finland | -1,04 | -1,25 | -0,84 |
| YLDs | Female | France | -0,01 | -0,09 | 0,07 |
| YLDs | Female | Germany | -0,59 | -0,62 | -0,56 |
| YLDs | Female | Greece | -1,67 | -1,77 | -1,57 |
| YLDs | Female | Hungary | -1,77 | -1,96 | -1,58 |
| YLDs | Female | Iceland | -1,23 | -1,30 | -1,15 |
| YLDs | Female | Ireland | -1,85 | -1,99 | -1,70 |
| YLDs | Female | Israel | -1,35 | -1,52 | -1,17 |
| YLDs | Female | Italy | -1,02 | -1,09 | -0,95 |
| YLDs | Female | Latvia | -1,39 | -1,50 | -1,28 |
| YLDs | Female | Lithuania | -1,19 | -1,58 | -0,80 |
| YLDs | Female | Luxembourg | -2,18 | -2,38 | -1,98 |
| YLDs | Female | Malta | -1,73 | -1,89 | -1,58 |
| YLDs | Female | Monaco | -1,47 | -1,61 | -1,33 |
| YLDs | Female | Montenegro | -1,13 | -1,24 | -1,01 |
| YLDs | Female | Netherlands | -1,41 | -1,56 | -1,26 |
| YLDs | Female | North Macedonia | -1,11 | -1,24 | -0,98 |
| YLDs | Female | Norway | -1,28 | -1,37 | -1,18 |
| YLDs | Female | Poland | -0,48 | -0,58 | -0,38 |
| YLDs | Female | Portugal | -2,97 | -3,23 | -2,70 |
| YLDs | Female | Republic of Moldova | -0,64 | -0,74 | -0,55 |
| YLDs | Female | Romania | -1,51 | -1,65 | -1,36 |
| YLDs | Female | Russian Federation | -0,96 | -1,08 | -0,85 |
| YLDs | Female | San Marino | -1,12 | -1,20 | -1,04 |
| YLDs | Female | Serbia | -1,46 | -1,58 | -1,34 |
| YLDs | Female | Slovakia | -1,70 | -2,00 | -1,40 |
| YLDs | Female | Slovenia | -1,45 | -1,53 | -1,37 |
| YLDs | Female | Spain | -1,60 | -1,70 | -1,49 |
| YLDs | Female | Sweden | -0,51 | -0,60 | -0,41 |
| YLDs | Female | Switzerland | -1,23 | -1,30 | -1,15 |
| YLDs | Female | Ukraine | -1,19 | -1,31 | -1,07 |
| YLDs | Female | United Kingdom | -1,43 | -1,52 | -1,33 |
| YLDs | Male | Albania | -1,38 | -1,51 | -1,25 |
| YLDs | Male | Andorra | -1,61 | -1,72 | -1,50 |
| YLDs | Male | Austria | -0,54 | -0,81 | -0,26 |
| YLDs | Male | Belarus | -1,15 | -1,29 | -1,00 |
| YLDs | Male | Belgium | -1,26 | -1,32 | -1,20 |
| YLDs | Male | Bosnia and Herzegovina | -1,11 | -1,21 | -1,02 |
| YLDs | Male | Bulgaria | -0,70 | -0,74 | -0,66 |
| YLDs | Male | Croatia | -1,11 | -1,27 | -0,95 |
| YLDs | Male | Cyprus | -1,56 | -1,75 | -1,37 |
| YLDs | Male | Czechia | -2,10 | -2,26 | -1,95 |
| YLDs | Male | Denmark | -1,76 | -1,94 | -1,59 |
| YLDs | Male | Estonia | -1,71 | -1,86 | -1,56 |
| YLDs | Male | Finland | -1,65 | -1,87 | -1,42 |
| YLDs | Male | France | 0,06 | 0,01 | 0,12 |
| YLDs | Male | Germany | -0,17 | -0,21 | -0,12 |
| YLDs | Male | Greece | -1,61 | -1,70 | -1,52 |
| YLDs | Male | Hungary | -1,70 | -1,84 | -1,56 |
| YLDs | Male | Iceland | -1,66 | -1,77 | -1,54 |
| YLDs | Male | Ireland | -2,21 | -2,40 | -2,01 |
| YLDs | Male | Israel | -1,96 | -2,26 | -1,66 |
| YLDs | Male | Italy | -0,85 | -0,91 | -0,78 |
| YLDs | Male | Latvia | -1,10 | -1,26 | -0,95 |
| YLDs | Male | Lithuania | -0,32 | -0,66 | 0,03 |
| YLDs | Male | Luxembourg | -2,29 | -2,50 | -2,08 |
| YLDs | Male | Malta | -2,17 | -2,38 | -1,97 |
| YLDs | Male | Monaco | -2,78 | -3,02 | -2,54 |
| YLDs | Male | Montenegro | -1,24 | -1,37 | -1,11 |
| YLDs | Male | Netherlands | -1,53 | -1,61 | -1,45 |
| YLDs | Male | North Macedonia | -0,59 | -0,64 | -0,53 |
| YLDs | Male | Norway | -1,32 | -1,44 | -1,20 |
| YLDs | Male | Poland | 0,09 | 0,05 | 0,13 |
| YLDs | Male | Portugal | -2,20 | -2,37 | -2,04 |
| YLDs | Male | Republic of Moldova | -0,06 | -0,17 | 0,05 |
| YLDs | Male | Romania | -1,33 | -1,47 | -1,19 |
| YLDs | Male | Russian Federation | -0,31 | -0,38 | -0,25 |
| YLDs | Male | San Marino | -1,86 | -1,99 | -1,73 |
| YLDs | Male | Serbia | -1,59 | -1,70 | -1,47 |
| YLDs | Male | Slovakia | -2,05 | -2,38 | -1,72 |
| YLDs | Male | Slovenia | -1,76 | -1,86 | -1,66 |
| YLDs | Male | Spain | -0,46 | -0,53 | -0,39 |
| YLDs | Male | Sweden | -0,79 | -0,93 | -0,65 |
| YLDs | Male | Switzerland | -1,41 | -1,48 | -1,33 |
| YLDs | Male | Ukraine | -1,46 | -1,59 | -1,33 |
| YLDs | Male | United Kingdom | -1,82 | -1,95 | -1,68 |
| YLSs | Both | Albania | -2,05 | -2,24 | -1,86 |
| YLSs | Both | Andorra | -3,36 | -3,57 | -3,15 |
| YLSs | Both | Austria | -5,49 | -5,93 | -5,04 |
| YLSs | Both | Belarus | -2,61 | -3,05 | -2,17 |
| YLSs | Both | Belgium | -4,17 | -4,34 | -4,00 |
| YLSs | Both | Bosnia and Herzegovina | -1,58 | -1,72 | -1,45 |
| YLSs | Both | Bulgaria | -1,12 | -1,20 | -1,03 |
| YLSs | Both | Croatia | -3,74 | -4,04 | -3,43 |
| YLSs | Both | Cyprus | -4,70 | -4,88 | -4,51 |
| YLSs | Both | Czechia | -5,89 | -6,20 | -5,59 |
| YLSs | Both | Denmark | -3,76 | -3,96 | -3,55 |
| YLSs | Both | Estonia | -6,71 | -7,38 | -6,04 |
| YLSs | Both | Finland | -3,95 | -4,18 | -3,72 |
| YLSs | Both | France | -3,50 | -3,70 | -3,30 |
| YLSs | Both | Germany | -4,21 | -4,62 | -3,79 |
| YLSs | Both | Greece | -4,87 | -5,15 | -4,58 |
| YLSs | Both | Hungary | -3,86 | -4,09 | -3,63 |
| YLSs | Both | Iceland | -3,95 | -4,15 | -3,75 |
| YLSs | Both | Ireland | -5,39 | -5,58 | -5,20 |
| YLSs | Both | Israel | -4,51 | -4,73 | -4,28 |
| YLSs | Both | Italy | -4,38 | -4,69 | -4,07 |
| YLSs | Both | Latvia | -2,30 | -2,54 | -2,06 |
| YLSs | Both | Lithuania | -1,84 | -2,20 | -1,49 |
| YLSs | Both | Luxembourg | -5,78 | -6,05 | -5,52 |
| YLSs | Both | Malta | -4,90 | -5,06 | -4,74 |
| YLSs | Both | Monaco | -4,97 | -5,48 | -4,46 |
| YLSs | Both | Montenegro | -1,97 | -2,34 | -1,60 |
| YLSs | Both | Netherlands | -3,55 | -3,77 | -3,33 |
| YLSs | Both | North Macedonia | -1,83 | -2,14 | -1,53 |
| YLSs | Both | Norway | -4,99 | -5,19 | -4,79 |
| YLSs | Both | Poland | -3,88 | -4,01 | -3,75 |
| YLSs | Both | Portugal | -6,06 | -6,36 | -5,76 |
| YLSs | Both | Republic of Moldova | -1,28 | -1,81 | -0,75 |
| YLSs | Both | Romania | -2,47 | -2,80 | -2,13 |
| YLSs | Both | Russian Federation | -3,05 | -3,64 | -2,47 |
| YLSs | Both | San Marino | -4,68 | -4,88 | -4,47 |
| YLSs | Both | Serbia | -2,77 | -3,03 | -2,51 |
| YLSs | Both | Slovakia | -3,25 | -3,40 | -3,10 |
| YLSs | Both | Slovenia | -4,56 | -4,81 | -4,31 |
| YLSs | Both | Spain | -5,16 | -5,53 | -4,79 |
| YLSs | Both | Sweden | -4,11 | -4,34 | -3,87 |
| YLSs | Both | Switzerland | -4,16 | -4,39 | -3,92 |
| YLSs | Both | Ukraine | -3,22 | -3,49 | -2,94 |
| YLSs | Both | United Kingdom | -4,94 | -5,22 | -4,67 |
| YLSs | Female | Albania | -2,06 | -2,25 | -1,86 |
| YLSs | Female | Andorra | -3,40 | -3,67 | -3,13 |
| YLSs | Female | Austria | -5,64 | -6,06 | -5,21 |
| YLSs | Female | Belarus | -2,71 | -3,11 | -2,31 |
| YLSs | Female | Belgium | -4,19 | -4,37 | -4,01 |
| YLSs | Female | Bosnia and Herzegovina | -1,75 | -1,88 | -1,62 |
| YLSs | Female | Bulgaria | -1,34 | -1,43 | -1,26 |
| YLSs | Female | Croatia | -3,87 | -4,18 | -3,57 |
| YLSs | Female | Cyprus | -4,80 | -4,98 | -4,61 |
| YLSs | Female | Czechia | -6,04 | -6,39 | -5,70 |
| YLSs | Female | Denmark | -3,84 | -4,04 | -3,64 |
| YLSs | Female | Estonia | -7,59 | -8,29 | -6,89 |
| YLSs | Female | Finland | -3,93 | -4,19 | -3,68 |
| YLSs | Female | France | -3,52 | -3,73 | -3,32 |
| YLSs | Female | Germany | -4,35 | -4,72 | -3,98 |
| YLSs | Female | Greece | -5,13 | -5,43 | -4,83 |
| YLSs | Female | Hungary | -4,08 | -4,31 | -3,85 |
| YLSs | Female | Iceland | -3,63 | -3,88 | -3,37 |
| YLSs | Female | Ireland | -5,33 | -5,53 | -5,13 |
| YLSs | Female | Israel | -4,54 | -4,74 | -4,34 |
| YLSs | Female | Italy | -4,30 | -4,60 | -4,00 |
| YLSs | Female | Latvia | -2,59 | -2,84 | -2,34 |
| YLSs | Female | Lithuania | -2,39 | -2,81 | -1,96 |
| YLSs | Female | Luxembourg | -5,90 | -6,20 | -5,60 |
| YLSs | Female | Malta | -5,10 | -5,32 | -4,89 |
| YLSs | Female | Monaco | -4,77 | -5,15 | -4,39 |
| YLSs | Female | Montenegro | -1,97 | -2,32 | -1,61 |
| YLSs | Female | Netherlands | -3,51 | -3,72 | -3,31 |
| YLSs | Female | North Macedonia | -1,85 | -2,18 | -1,52 |
| YLSs | Female | Norway | -4,93 | -5,17 | -4,69 |
| YLSs | Female | Poland | -4,30 | -4,44 | -4,15 |
| YLSs | Female | Portugal | -6,16 | -6,44 | -5,88 |
| YLSs | Female | Republic of Moldova | -1,84 | -2,39 | -1,28 |
| YLSs | Female | Romania | -2,70 | -3,02 | -2,38 |
| YLSs | Female | Russian Federation | -3,38 | -3,95 | -2,80 |
| YLSs | Female | San Marino | -4,31 | -4,53 | -4,10 |
| YLSs | Female | Serbia | -2,84 | -3,10 | -2,57 |
| YLSs | Female | Slovakia | -3,65 | -3,79 | -3,51 |
| YLSs | Female | Slovenia | -4,77 | -4,96 | -4,58 |
| YLSs | Female | Spain | -5,44 | -5,77 | -5,10 |
| YLSs | Female | Sweden | -4,06 | -4,33 | -3,79 |
| YLSs | Female | Switzerland | -4,12 | -4,35 | -3,90 |
| YLSs | Female | Ukraine | -3,49 | -3,76 | -3,22 |
| YLSs | Female | United Kingdom | -4,91 | -5,18 | -4,64 |
| YLSs | Male | Albania | -2,02 | -2,22 | -1,83 |
| YLSs | Male | Andorra | -3,34 | -3,50 | -3,18 |
| YLSs | Male | Austria | -5,52 | -5,98 | -5,05 |
| YLSs | Male | Belarus | -2,51 | -2,96 | -2,06 |
| YLSs | Male | Belgium | -4,29 | -4,45 | -4,13 |
| YLSs | Male | Bosnia and Herzegovina | -1,43 | -1,58 | -1,28 |
| YLSs | Male | Bulgaria | -0,92 | -1,01 | -0,82 |
| YLSs | Male | Croatia | -3,75 | -4,07 | -3,43 |
| YLSs | Male | Cyprus | -4,60 | -4,79 | -4,40 |
| YLSs | Male | Czechia | -5,90 | -6,17 | -5,62 |
| YLSs | Male | Denmark | -3,79 | -4,02 | -3,57 |
| YLSs | Male | Estonia | -5,90 | -6,51 | -5,28 |
| YLSs | Male | Finland | -4,10 | -4,33 | -3,87 |
| YLSs | Male | France | -3,66 | -3,87 | -3,45 |
| YLSs | Male | Germany | -4,33 | -4,81 | -3,85 |
| YLSs | Male | Greece | -4,57 | -4,85 | -4,30 |
| YLSs | Male | Hungary | -3,70 | -3,94 | -3,47 |
| YLSs | Male | Iceland | -4,36 | -4,57 | -4,15 |
| YLSs | Male | Ireland | -5,53 | -5,73 | -5,33 |
| YLSs | Male | Israel | -4,56 | -4,82 | -4,29 |
| YLSs | Male | Italy | -4,60 | -4,91 | -4,28 |
| YLSs | Male | Latvia | -2,08 | -2,32 | -1,84 |
| YLSs | Male | Lithuania | -1,32 | -1,62 | -1,01 |
| YLSs | Male | Luxembourg | -5,75 | -6,00 | -5,51 |
| YLSs | Male | Malta | -4,72 | -4,88 | -4,57 |
| YLSs | Male | Monaco | -5,32 | -6,01 | -4,64 |
| YLSs | Male | Montenegro | -2,00 | -2,36 | -1,63 |
| YLSs | Male | Netherlands | -3,73 | -3,97 | -3,48 |
| YLSs | Male | North Macedonia | -1,85 | -2,13 | -1,56 |
| YLSs | Male | Norway | -5,16 | -5,35 | -4,96 |
| YLSs | Male | Poland | -3,57 | -3,70 | -3,44 |
| YLSs | Male | Portugal | -6,01 | -6,33 | -5,69 |
| YLSs | Male | Republic of Moldova | -0,76 | -1,27 | -0,25 |
| YLSs | Male | Romania | -2,22 | -2,57 | -1,86 |
| YLSs | Male | Russian Federation | -2,86 | -3,43 | -2,28 |
| YLSs | Male | San Marino | -5,15 | -5,37 | -4,93 |
| YLSs | Male | Serbia | -2,75 | -3,00 | -2,49 |
| YLSs | Male | Slovakia | -2,95 | -3,13 | -2,76 |
| YLSs | Male | Slovenia | -4,57 | -4,88 | -4,26 |
| YLSs | Male | Spain | -4,97 | -5,38 | -4,56 |
| YLSs | Male | Sweden | -4,22 | -4,44 | -4,00 |
| YLSs | Male | Switzerland | -4,32 | -4,55 | -4,08 |
| YLSs | Male | Ukraine | -2,98 | -3,24 | -2,72 |
| YLSs | Male | United Kingdom | -5,04 | -5,33 | -4,75 |
