# Supplementary material for: Trends in mortality and disability from ischaemic stroke in Europe, 1990-2023
Source: Eur Stroke J. 2026 Jul 21;11(7):aakag082. doi: 10.1093/esj/aakag082 (PMC13387428; doi:10.1093/esj/aakag082)
Supplement: Supplementary_material_aakag082 [file supplementary_material_aakag082.zip › Supplementary Table 2.docx]

**Supplementary Table 2.** Baseline (1990) and end‑period (2023) absolute rates of DALYs, YLLs, and YLDs per 100,000 population, and absolute and percentage changes by country and sex (F=female; M=male)

| **Country** | **Sex** | **DALY 1990** | **DALY 2023** | **Δ DALY (abs)** | **Δ DALY (%)** | **YLL 1990** | **YLL 2023** | **Δ YLL (abs)** | **Δ YLL (%)** | **YLD 1990** | **YLD 2023** | **Δ YLD (abs)** | **Δ YLD (%)** |
| --- | --- | --- | --- | --- | --- | --- | --- | --- | --- | --- | --- | --- | --- |
| Albania | Both | 858 | 432 | -426 | -49,6 | 725 | 331 | -394 | -54,3 | 133 | 101 | -32 | -24,1 |
| Albania | F | 764 | 405 | -359 | -47,0 | 638 | 300 | -338 | -53,0 | 125 | 105 | -21 | -16,5 |
| Albania | M | 966 | 466 | -500 | -51,8 | 820 | 367 | -453 | -55,2 | 146 | 98 | -48 | -32,6 |
| Andorra | Both | 520 | 221 | -299 | -57,5 | 401 | 143 | -258 | -64,3 | 118 | 77 | -41 | -34,8 |
| Andorra | F | 512 | 217 | -296 | -57,7 | 402 | 143 | -259 | -64,3 | 110 | 73 | -37 | -33,6 |
| Andorra | M | 525 | 224 | -301 | -57,3 | 398 | 142 | -256 | -64,3 | 127 | 82 | -45 | -35,6 |
| Austria | Both | 1043 | 285 | -759 | -72,7 | 922 | 176 | -746 | -80,9 | 122 | 108 | -13 | -10,9 |
| Austria | F | 902 | 242 | -660 | -73,2 | 798 | 146 | -651 | -81,7 | 105 | 96 | -9 | -8,5 |
| Austria | M | 1276 | 337 | -939 | -73,6 | 1123 | 212 | -911 | -81,1 | 153 | 125 | -28 | -18,5 |
| Belarus | Both | 2208 | 1250 | -959 | -43,4 | 2010 | 1105 | -905 | -45,0 | 198 | 145 | -53 | -26,9 |
| Belarus | F | 1806 | 991 | -814 | -45,1 | 1627 | 860 | -767 | -47,1 | 178 | 131 | -47 | -26,4 |
| Belarus | M | 2865 | 1647 | -1218 | -42,5 | 2628 | 1476 | -1151 | -43,8 | 237 | 170 | -67 | -28,1 |
| Belgium | Both | 882 | 262 | -621 | -70,3 | 775 | 187 | -588 | -75,8 | 107 | 74 | -33 | -30,4 |
| Belgium | F | 791 | 234 | -556 | -70,4 | 694 | 167 | -527 | -76,0 | 97 | 68 | -29 | -30,0 |
| Belgium | M | 1016 | 292 | -724 | -71,3 | 892 | 209 | -683 | -76,6 | 124 | 83 | -41 | -33,1 |
| Bosnia and Herzegovina | Both | 2436 | 1437 | -999 | -41,0 | 2219 | 1274 | -946 | -42,6 | 217 | 163 | -53 | -24,6 |
| Bosnia and Herzegovina | F | 2333 | 1328 | -1005 | -43,1 | 2151 | 1189 | -962 | -44,7 | 181 | 139 | -42 | -23,3 |
| Bosnia and Herzegovina | M | 2556 | 1546 | -1010 | -39,5 | 2287 | 1350 | -938 | -41,0 | 269 | 196 | -72 | -26,9 |
| Bulgaria | Both | 2791 | 1901 | -890 | -31,9 | 2537 | 1690 | -847 | -33,4 | 254 | 212 | -43 | -16,8 |
| Bulgaria | F | 2449 | 1565 | -884 | -36,1 | 2225 | 1377 | -848 | -38,1 | 224 | 188 | -36 | -16,0 |
| Bulgaria | M | 3222 | 2316 | -907 | -28,1 | 2928 | 2073 | -855 | -29,2 | 295 | 243 | -52 | -17,5 |
| Croatia | Both | 1968 | 649 | -1319 | -67,0 | 1805 | 537 | -1268 | -70,3 | 163 | 112 | -51 | -31,2 |
| Croatia | F | 1755 | 555 | -1200 | -68,4 | 1616 | 464 | -1152 | -71,3 | 139 | 91 | -48 | -34,6 |
| Croatia | M | 2288 | 757 | -1531 | -66,9 | 2084 | 614 | -1470 | -70,5 | 204 | 143 | -61 | -30,1 |
| Cyprus | Both | 1298 | 346 | -952 | -73,3 | 1208 | 281 | -927 | -76,7 | 90 | 65 | -24 | -27,2 |
| Cyprus | F | 1268 | 324 | -943 | -74,4 | 1197 | 268 | -929 | -77,6 | 71 | 56 | -14 | -20,3 |
| Cyprus | M | 1328 | 373 | -955 | -71,9 | 1216 | 297 | -919 | -75,6 | 112 | 76 | -37 | -32,7 |
| Czechia | Both | 2653 | 513 | -2141 | -80,7 | 2448 | 402 | -2046 | -83,6 | 206 | 111 | -95 | -46,3 |
| Czechia | F | 2302 | 432 | -1870 | -81,2 | 2121 | 334 | -1788 | -84,3 | 180 | 99 | -82 | -45,4 |
| Czechia | M | 3174 | 606 | -2569 | -80,9 | 2927 | 478 | -2449 | -83,7 | 248 | 127 | -120 | -48,5 |
| Denmark | Both | 812 | 313 | -499 | -61,5 | 684 | 232 | -452 | -66,1 | 127 | 80 | -47 | -36,8 |
| Denmark | F | 700 | 264 | -436 | -62,3 | 590 | 192 | -397 | -67,4 | 110 | 71 | -39 | -35,2 |
| Denmark | M | 958 | 365 | -592 | -61,9 | 806 | 274 | -531 | -66,0 | 152 | 91 | -61 | -40,2 |
| Estonia | Both | 2325 | 492 | -1833 | -78,8 | 2151 | 387 | -1764 | -82,0 | 174 | 105 | -69 | -39,6 |
| Estonia | F | 2009 | 348 | -1661 | -82,7 | 1853 | 253 | -1600 | -86,3 | 156 | 95 | -61 | -39,3 |
| Estonia | M | 2857 | 720 | -2137 | -74,8 | 2650 | 596 | -2054 | -77,5 | 207 | 124 | -83 | -40,1 |
| Finland | Both | 1059 | 364 | -695 | -65,6 | 912 | 265 | -647 | -71,0 | 147 | 99 | -48 | -32,6 |
| Finland | F | 902 | 316 | -586 | -65,0 | 779 | 228 | -551 | -70,7 | 124 | 88 | -36 | -28,9 |
| Finland | M | 1280 | 416 | -863 | -67,5 | 1092 | 303 | -789 | -72,3 | 187 | 113 | -74 | -39,5 |
| France | Both | 601 | 251 | -349 | -58,2 | 518 | 167 | -351 | -67,8 | 83 | 84 | 1 | 1,6 |
| France | F | 499 | 206 | -293 | -58,7 | 425 | 133 | -293 | -68,8 | 74 | 74 | 0 | -0,5 |
| France | M | 754 | 305 | -449 | -59,6 | 657 | 207 | -450 | -68,5 | 97 | 98 | 1 | 0,8 |
| Germany | Both | 1090 | 397 | -692 | -63,5 | 939 | 262 | -678 | -72,2 | 150 | 136 | -15 | -9,6 |
| Germany | F | 941 | 325 | -616 | -65,5 | 802 | 209 | -593 | -73,9 | 139 | 116 | -23 | -16,9 |
| Germany | M | 1339 | 481 | -857 | -64,0 | 1167 | 320 | -847 | -72,6 | 172 | 161 | -10 | -5,9 |
| Greece | Both | 1465 | 419 | -1046 | -71,4 | 1325 | 332 | -993 | -74,9 | 141 | 87 | -54 | -38,1 |
| Greece | F | 1492 | 394 | -1098 | -73,6 | 1361 | 313 | -1048 | -77,0 | 131 | 81 | -51 | -38,5 |
| Greece | M | 1424 | 445 | -979 | -68,7 | 1271 | 350 | -921 | -72,4 | 153 | 95 | -58 | -37,9 |
| Hungary | Both | 2333 | 817 | -1516 | -65,0 | 2108 | 682 | -1426 | -67,7 | 225 | 135 | -90 | -40,0 |
| Hungary | F | 1947 | 642 | -1305 | -67,0 | 1748 | 522 | -1225 | -70,1 | 199 | 120 | -79 | -39,7 |
| Hungary | M | 2865 | 1052 | -1813 | -63,3 | 2598 | 890 | -1708 | -65,7 | 266 | 161 | -105 | -39,4 |
| Iceland | Both | 674 | 244 | -430 | -63,8 | 562 | 170 | -393 | -69,8 | 112 | 74 | -38 | -33,6 |
| Iceland | F | 568 | 230 | -338 | -59,6 | 471 | 161 | -310 | -65,8 | 97 | 68 | -28 | -29,4 |
| Iceland | M | 809 | 256 | -552 | -68,3 | 677 | 175 | -502 | -74,1 | 132 | 81 | -51 | -38,5 |
| Ireland | Both | 937 | 220 | -717 | -76,5 | 832 | 159 | -673 | -80,9 | 105 | 61 | -44 | -42,1 |
| Ireland | F | 833 | 199 | -634 | -76,1 | 743 | 144 | -599 | -80,6 | 91 | 55 | -35 | -38,8 |
| Ireland | M | 1057 | 240 | -816 | -77,2 | 931 | 173 | -758 | -81,4 | 126 | 68 | -58 | -46,2 |
| Israel | Both | 626 | 210 | -417 | -66,5 | 486 | 128 | -358 | -73,6 | 140 | 81 | -59 | -41,9 |
| Israel | F | 565 | 188 | -377 | -66,7 | 455 | 117 | -338 | -74,3 | 109 | 71 | -38 | -35,0 |
| Israel | M | 701 | 233 | -468 | -66,8 | 522 | 138 | -384 | -73,5 | 178 | 94 | -84 | -47,1 |
| Italy | Both | 956 | 288 | -668 | -69,9 | 868 | 219 | -648 | -74,7 | 88 | 69 | -20 | -22,2 |
| Italy | F | 828 | 251 | -577 | -69,7 | 750 | 193 | -557 | -74,3 | 77 | 58 | -20 | -25,3 |
| Italy | M | 1151 | 332 | -819 | -71,1 | 1046 | 250 | -797 | -76,1 | 105 | 82 | -22 | -21,3 |
| Latvia | Both | 2494 | 1414 | -1080 | -43,3 | 2289 | 1273 | -1016 | -44,4 | 205 | 141 | -63 | -30,9 |
| Latvia | F | 2183 | 1123 | -1061 | -48,6 | 1999 | 1000 | -999 | -50,0 | 184 | 123 | -61 | -33,3 |
| Latvia | M | 3030 | 1854 | -1176 | -38,8 | 2785 | 1680 | -1105 | -39,7 | 245 | 174 | -72 | -29,2 |
| Lithuania | Both | 1490 | 909 | -581 | -39,0 | 1322 | 764 | -558 | -42,2 | 168 | 145 | -23 | -13,5 |
| Lithuania | F | 1319 | 683 | -636 | -48,2 | 1150 | 554 | -596 | -51,8 | 169 | 129 | -40 | -23,7 |
| Lithuania | M | 1747 | 1238 | -508 | -29,1 | 1579 | 1065 | -514 | -32,5 | 168 | 174 | 5 | 3,2 |
| Luxembourg | Both | 1378 | 270 | -1108 | -80,4 | 1263 | 204 | -1060 | -83,9 | 115 | 66 | -49 | -42,5 |
| Luxembourg | F | 1231 | 239 | -992 | -80,6 | 1128 | 180 | -948 | -84,0 | 102 | 59 | -43 | -42,4 |
| Luxembourg | M | 1594 | 310 | -1284 | -80,5 | 1458 | 235 | -1224 | -83,9 | 135 | 75 | -60 | -44,4 |
| Malta | Both | 1016 | 248 | -768 | -75,6 | 911 | 184 | -726 | -79,7 | 105 | 64 | -41 | -39,3 |
| Malta | F | 942 | 220 | -722 | -76,6 | 857 | 166 | -690 | -80,6 | 86 | 54 | -32 | -37,1 |
| Malta | M | 1105 | 279 | -826 | -74,8 | 974 | 204 | -771 | -79,1 | 131 | 75 | -56 | -42,8 |
| Monaco | Both | 1404 | 306 | -1098 | -78,2 | 1245 | 219 | -1026 | -82,4 | 159 | 87 | -72 | -45,1 |
| Monaco | F | 1292 | 316 | -977 | -75,6 | 1166 | 232 | -934 | -80,1 | 126 | 83 | -43 | -34,0 |
| Monaco | M | 1536 | 283 | -1253 | -81,6 | 1332 | 190 | -1142 | -85,7 | 203 | 93 | -111 | -54,4 |
| Montenegro | Both | 2116 | 1265 | -851 | -40,2 | 1935 | 1134 | -801 | -41,4 | 181 | 131 | -50 | -27,8 |
| Montenegro | F | 2146 | 1287 | -859 | -40,0 | 1986 | 1171 | -816 | -41,1 | 159 | 116 | -43 | -27,1 |
| Montenegro | M | 2053 | 1201 | -852 | -41,5 | 1842 | 1052 | -790 | -42,9 | 211 | 149 | -62 | -29,3 |
| Netherlands | Both | 764 | 318 | -446 | -58,4 | 636 | 233 | -403 | -63,4 | 128 | 85 | -43 | -33,5 |
| Netherlands | F | 674 | 281 | -393 | -58,3 | 561 | 206 | -355 | -63,3 | 113 | 75 | -38 | -33,5 |
| Netherlands | M | 889 | 355 | -534 | -60,0 | 739 | 259 | -480 | -64,9 | 151 | 96 | -54 | -36,1 |
| North Macedonia | Both | 3316 | 1971 | -1345 | -40,6 | 3068 | 1777 | -1291 | -42,1 | 248 | 194 | -54 | -21,7 |
| North Macedonia | F | 3142 | 1865 | -1277 | -40,6 | 2926 | 1709 | -1217 | -41,6 | 216 | 156 | -60 | -27,8 |
| North Macedonia | M | 3518 | 2080 | -1438 | -40,9 | 3232 | 1836 | -1395 | -43,2 | 287 | 244 | -43 | -15,0 |
| Norway | Both | 941 | 274 | -667 | -70,8 | 807 | 179 | -628 | -77,8 | 134 | 95 | -39 | -29,1 |
| Norway | F | 818 | 240 | -578 | -70,6 | 710 | 164 | -546 | -76,8 | 109 | 76 | -33 | -30,0 |
| Norway | M | 1101 | 309 | -792 | -72,0 | 930 | 191 | -739 | -79,4 | 171 | 117 | -53 | -31,4 |
| Poland | Both | 2040 | 668 | -1372 | -67,2 | 1913 | 549 | -1365 | -71,3 | 127 | 120 | -7 | -5,5 |
| Poland | F | 1820 | 525 | -1296 | -71,2 | 1707 | 426 | -1282 | -75,1 | 113 | 99 | -14 | -12,5 |
| Poland | M | 2324 | 844 | -1481 | -63,7 | 2175 | 692 | -1482 | -68,2 | 149 | 151 | 2 | 1,2 |
| Portugal | Both | 2307 | 434 | -1872 | -81,2 | 2110 | 340 | -1770 | -83,9 | 197 | 95 | -102 | -52,0 |
| Portugal | F | 2068 | 372 | -1695 | -82,0 | 1874 | 289 | -1585 | -84,6 | 193 | 83 | -111 | -57,2 |
| Portugal | M | 2631 | 510 | -2121 | -80,6 | 2429 | 399 | -2030 | -83,6 | 201 | 111 | -90 | -44,9 |
| Republic of Moldova | Both | 1452 | 884 | -567 | -39,1 | 1304 | 754 | -551 | -42,2 | 147 | 131 | -17 | -11,3 |
| Republic of Moldova | F | 1386 | 693 | -694 | -50,0 | 1249 | 579 | -670 | -53,6 | 138 | 114 | -24 | -17,4 |
| Republic of Moldova | M | 1553 | 1164 | -390 | -25,1 | 1388 | 1004 | -384 | -27,6 | 165 | 159 | -6 | -3,7 |
| Romania | Both | 2426 | 1237 | -1190 | -49,0 | 2224 | 1102 | -1122 | -50,5 | 202 | 135 | -67 | -33,3 |
| Romania | F | 2215 | 1027 | -1187 | -53,6 | 2045 | 916 | -1129 | -55,2 | 170 | 112 | -58 | -34,2 |
| Romania | M | 2694 | 1514 | -1180 | -43,8 | 2447 | 1343 | -1104 | -45,1 | 247 | 171 | -77 | -30,9 |
| Russian Federation | Both | 3092 | 1533 | -1560 | -50,4 | 2912 | 1382 | -1531 | -52,6 | 180 | 151 | -29 | -16,1 |
| Russian Federation | F | 2780 | 1227 | -1553 | -55,9 | 2602 | 1088 | -1514 | -58,2 | 178 | 139 | -39 | -21,8 |
| Russian Federation | M | 3692 | 1955 | -1738 | -47,1 | 3505 | 1785 | -1720 | -49,1 | 187 | 170 | -17 | -9,3 |
| San Marino | Both | 999 | 308 | -691 | -69,1 | 854 | 211 | -642 | -75,2 | 145 | 97 | -49 | -33,4 |
| San Marino | F | 797 | 269 | -528 | -66,2 | 681 | 184 | -498 | -73,1 | 116 | 85 | -30 | -26,2 |
| San Marino | M | 1276 | 349 | -928 | -72,7 | 1092 | 239 | -854 | -78,2 | 184 | 110 | -74 | -40,1 |
| Serbia | Both | 3815 | 1778 | -2036 | -53,4 | 3609 | 1644 | -1965 | -54,5 | 206 | 134 | -71 | -34,6 |
| Serbia | F | 3633 | 1675 | -1959 | -53,9 | 3459 | 1559 | -1900 | -54,9 | 174 | 116 | -58 | -33,5 |
| Serbia | M | 4000 | 1866 | -2135 | -53,4 | 3754 | 1707 | -2047 | -54,5 | 246 | 159 | -88 | -35,6 |
| Slovakia | Both | 1861 | 699 | -1162 | -62,4 | 1631 | 567 | -1064 | -65,2 | 229 | 132 | -97 | -42,4 |
| Slovakia | F | 1591 | 548 | -1043 | -65,6 | 1401 | 435 | -966 | -69,0 | 190 | 113 | -77 | -40,4 |
| Slovakia | M | 2223 | 894 | -1329 | -59,8 | 1933 | 733 | -1200 | -62,1 | 289 | 161 | -129 | -44,5 |
| Slovenia | Both | 1587 | 400 | -1187 | -74,8 | 1463 | 321 | -1142 | -78,1 | 124 | 79 | -45 | -36,3 |
| Slovenia | F | 1307 | 317 | -990 | -75,7 | 1201 | 248 | -953 | -79,4 | 106 | 69 | -36 | -34,4 |
| Slovenia | M | 2054 | 502 | -1552 | -75,6 | 1897 | 410 | -1487 | -78,4 | 156 | 92 | -65 | -41,3 |
| Spain | Both | 1011 | 259 | -752 | -74,4 | 890 | 168 | -721 | -81,1 | 121 | 91 | -31 | -25,4 |
| Spain | F | 923 | 205 | -718 | -77,7 | 818 | 139 | -678 | -83,0 | 106 | 66 | -39 | -37,3 |
| Spain | M | 1124 | 323 | -801 | -71,3 | 981 | 201 | -780 | -79,5 | 143 | 122 | -21 | -15,0 |
| Sweden | Both | 793 | 303 | -490 | -61,8 | 658 | 187 | -471 | -71,6 | 135 | 116 | -19 | -14,2 |
| Sweden | F | 685 | 258 | -427 | -62,3 | 578 | 164 | -414 | -71,6 | 107 | 95 | -13 | -12,0 |
| Sweden | M | 926 | 350 | -576 | -62,2 | 751 | 209 | -542 | -72,2 | 175 | 141 | -34 | -19,4 |
| Switzerland | Both | 667 | 208 | -458 | -68,8 | 574 | 144 | -430 | -74,9 | 92 | 64 | -28 | -30,6 |
| Switzerland | F | 571 | 179 | -391 | -68,6 | 492 | 123 | -368 | -74,9 | 79 | 56 | -23 | -29,0 |
| Switzerland | M | 802 | 242 | -561 | -69,9 | 690 | 168 | -522 | -75,7 | 112 | 74 | -39 | -34,4 |
| Ukraine | Both | 2867 | 1172 | -1696 | -59,1 | 2643 | 1014 | -1630 | -61,7 | 224 | 158 | -66 | -29,4 |
| Ukraine | F | 2487 | 918 | -1568 | -63,1 | 2285 | 777 | -1508 | -66,0 | 201 | 141 | -60 | -29,8 |
| Ukraine | M | 3545 | 1571 | -1974 | -55,7 | 3265 | 1383 | -1882 | -57,6 | 280 | 188 | -92 | -32,9 |
| United Kingdom | Both | 957 | 282 | -675 | -70,5 | 834 | 202 | -632 | -75,7 | 123 | 80 | -43 | -34,9 |
| United Kingdom | F | 851 | 256 | -595 | -69,9 | 741 | 182 | -559 | -75,5 | 110 | 74 | -36 | -32,5 |
| United Kingdom | M | 1090 | 309 | -782 | -71,7 | 948 | 222 | -726 | -76,6 | 142 | 87 | -55 | -39,0 |
