# Supplementary figures and images for: Trends in mortality and disability from ischaemic stroke in Europe, 1990-2023
Source: Eur Stroke J. 2026 Jul 21;11(7):aakag082. doi: 10.1093/esj/aakag082 (PMC13387428; doi:10.1093/esj/aakag082)

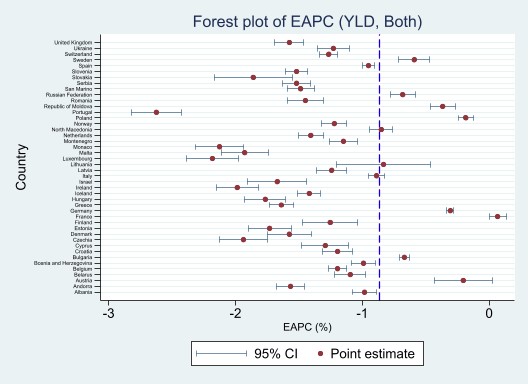

Supplement: Supplementary_material_aakag082 [file supplementary_material_aakag082.zip › Supplementary Figure 8.jpg]

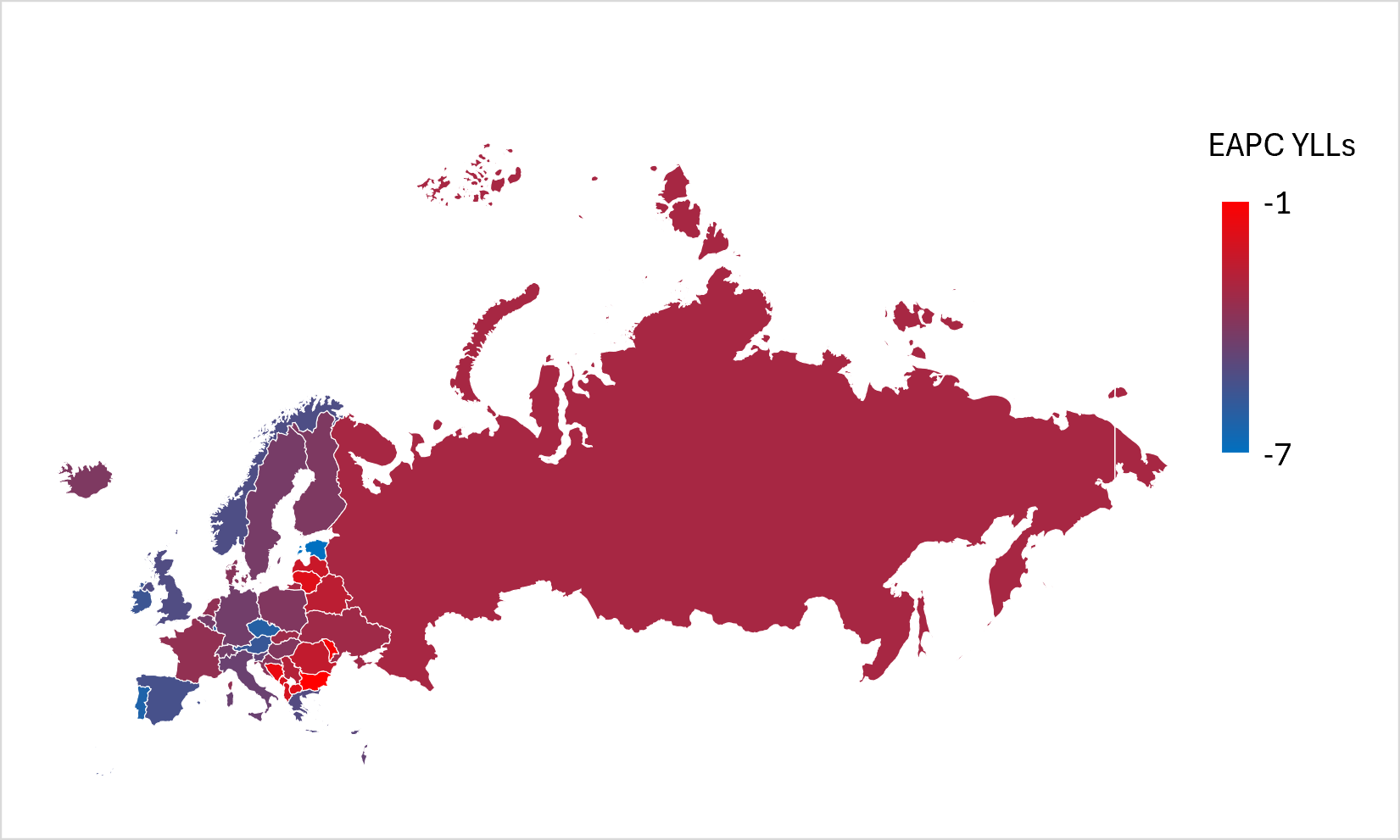

Supplement: Supplementary_material_aakag082 [file supplementary_material_aakag082.zip › Supplementary Figure 1.png]

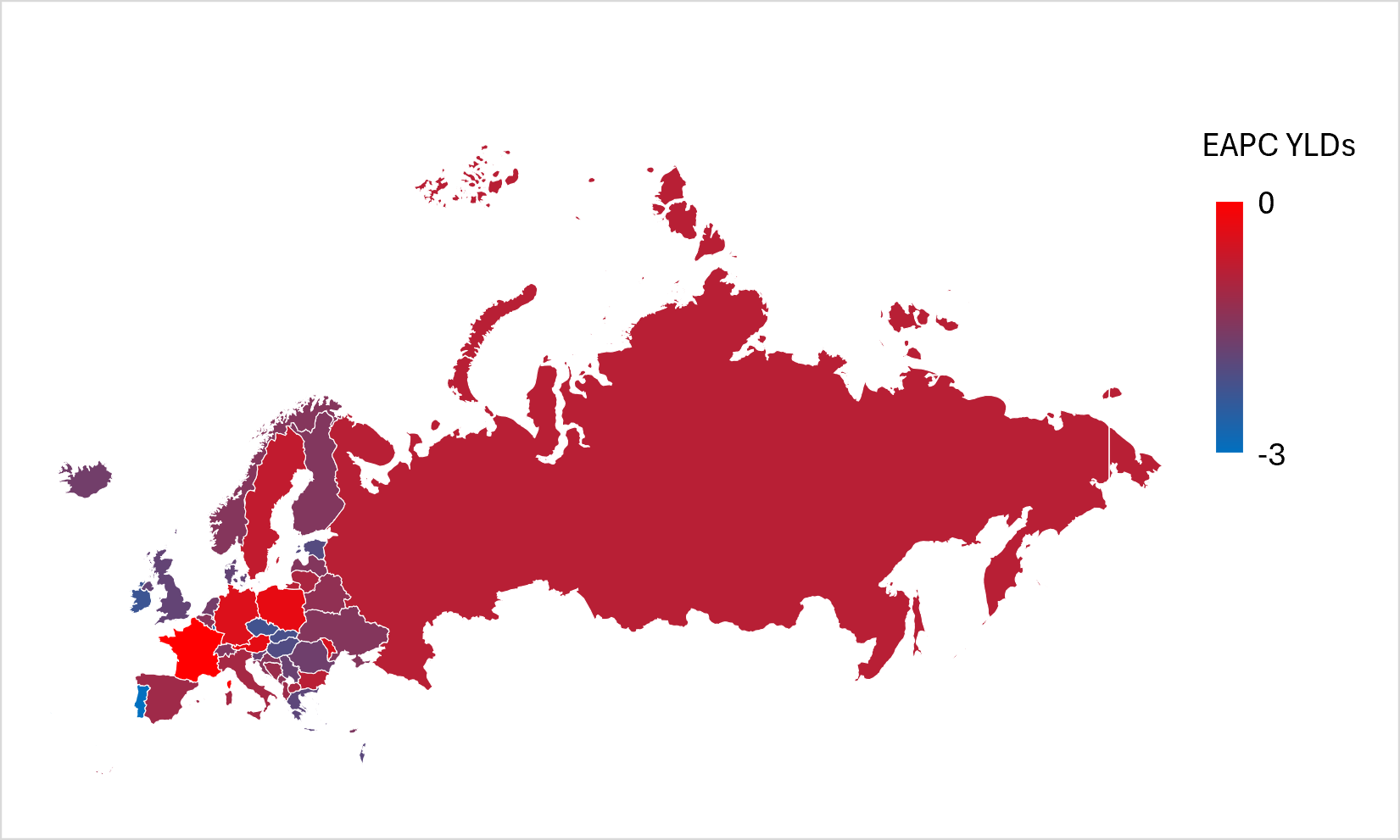

Supplement: Supplementary_material_aakag082 [file supplementary_material_aakag082.zip › Supplementary Figure 2.png]

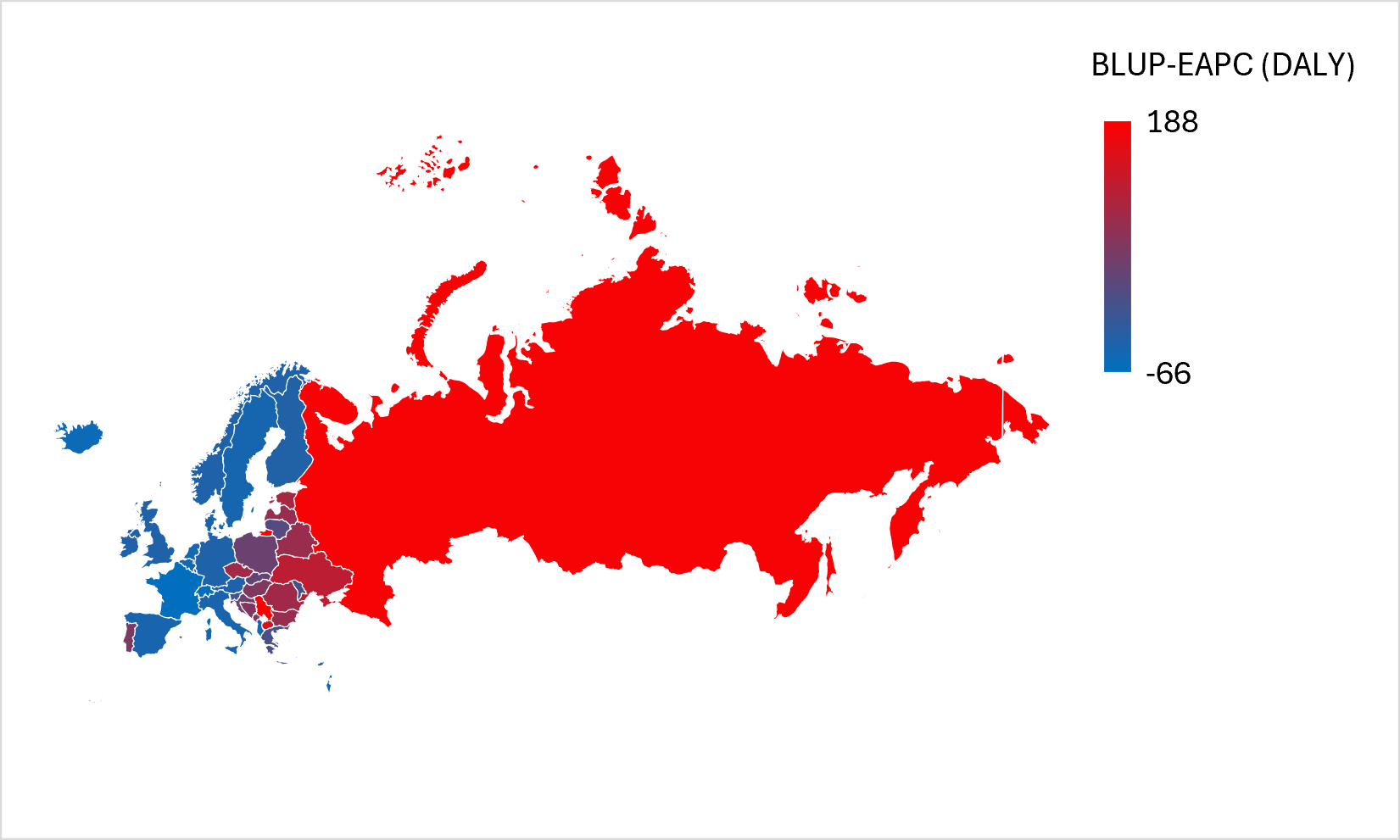

Supplement: Supplementary_material_aakag082 [file supplementary_material_aakag082.zip › Supplementary Figure 3.png]

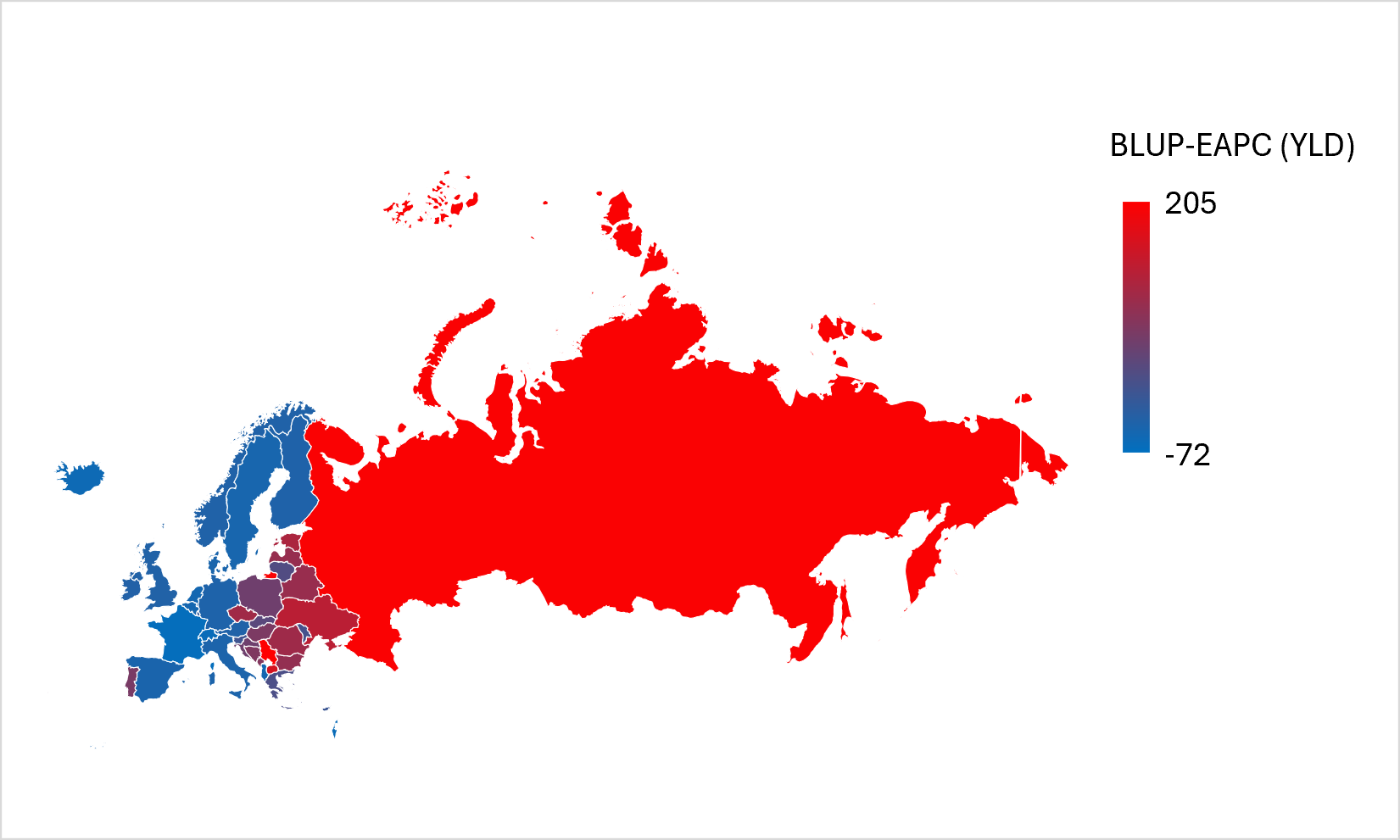

Supplement: Supplementary_material_aakag082 [file supplementary_material_aakag082.zip › Supplementary Figure 4.png]

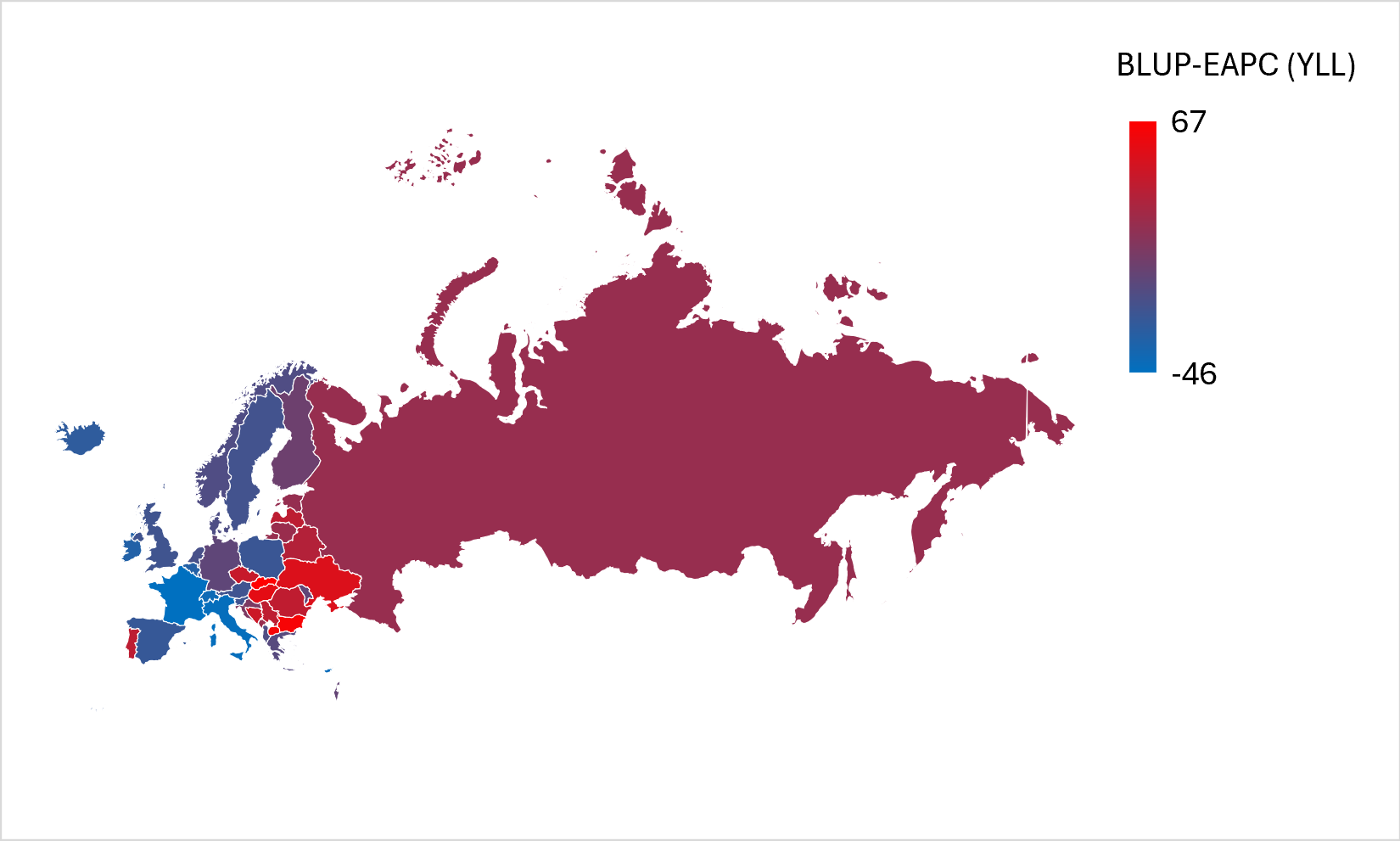

Supplement: Supplementary_material_aakag082 [file supplementary_material_aakag082.zip › Supplementary Figure 5.png]

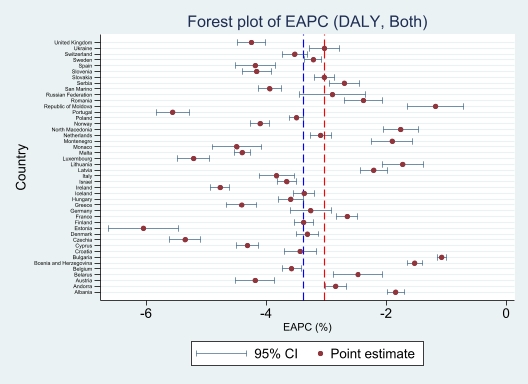

Supplement: Supplementary_material_aakag082 [file supplementary_material_aakag082.zip › Supplementary Figure 6.jpg]

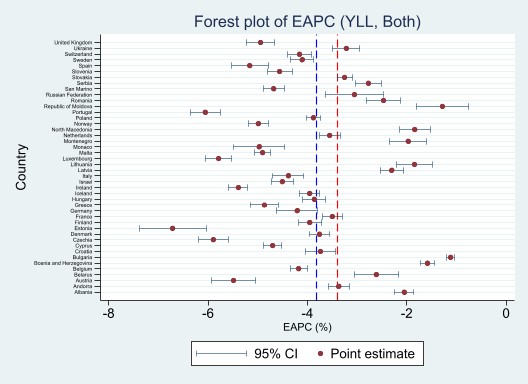

Supplement: Supplementary_material_aakag082 [file supplementary_material_aakag082.zip › Supplementary Figure 7.jpg]
